# Cryptic diversity of limestone karst inhabiting land snails (*Cyclophorus* spp.) in northern Vietnam, their evolutionary history and the description of four new species

**DOI:** 10.1371/journal.pone.0222163

**Published:** 2019-10-23

**Authors:** Katharina C. M. von Oheimb, Parm Viktor von Oheimb, Takahiro Hirano, Tu Van Do, Jonathan Ablett, Hao Van Luong, Sang Van Pham, Fred Naggs

**Affiliations:** 1 Life Sciences Department, The Natural History Museum, London, England, United Kingdom; 2 Museum für Naturkunde – Leibniz Institute for Evolution and Biodiversity Science, Berlin, Germany; 3 Department of Biological Sciences, University of Idaho, Moscow, Idaho, United States of America; 4 Institute of Ecology and Biological Resources, Vietnam Academy of Science and Technology, Ha Noi, Vietnam; 5 Graduate University of Science and Technology, Vietnam Academy of Science and Technology, Ha Noi, Vietnam; 6 Centre for Rescue and Conservation of Organisms, Hoang Lien National Park, Sa Pa, Vietnam; 7 Department of Specimen Preparation and Exhibitive Design, Vietnam National Museum of Nature, Vietnam Academy of Science and Technology, Ha Noi, Vietnam; National Cheng Kung University, TAIWAN

## Abstract

Limestone karsts can form terrestrial habitat islands for calcium-dependent organisms. In Vietnam, many karst habitats are threatened, while their rich biodiversity is still far from being thoroughly explored. Given that conservation of karst biota strongly relies on correct species identification, the presence of undetected cryptic species can pose severe problems. The present study focuses on cryptic diversity among karst-inhabiting land snails of the genus *Cyclophorus* in northern Vietnam, where specimens with a similar shell morphology have been reported from various regions. In order to examine the diversity and evolutionary history of this “widespread morphotype”, we generated a Bayesian phylogeny based on DNA sequence data. Automatic Barcode Gap Discovery (ABGD) and the Bayesian implementation of the Poisson tree processes model (bPTP) contributed to species delimitation and analyses of shell shape and size aided the morphological characterisation of individual species. We found that the examined specimens of the widespread morphotype did not form a single monophyletic group in the phylogeny but clustered into several different clades. We delimited nine different species that develop the widespread morphotype and described four of them as new. Processes of convergent evolution were probably involved in the origin of the delimited species, while their generally allopatric distribution could result from interspecific competition. Our findings indicate ongoing processes of speciation and a potential case of morphological character displacement. The high degree of morphological overlap found among the species underlines the importance of DNA sequence data for species delimitation and description in the genus *Cyclophorus*. Given the findings of the present study and the high potential that as yet undiscovered cryptic taxa have also evolved in other groups of karst-inhabiting organisms, we argue for a systematic and efficient detection and description of Vietnam’s karst biodiversity to provide a solid basis for future conservation planning.

## Introduction

Tropical and subtropical limestone karsts, such as those in Southeast Asia, can form terrestrial habitat islands for plant and animal taxa that depend on a calcium-rich environment. Such limestone-associated organisms can occur in high abundances on karsts, while the often acidic soil in the surroundings can isolate individual populations [1–3]. Karst habitats in Southeast Asia harbour a rich biodiversity, which includes various site-endemic taxa [4], and individual species communities on different karsts in the same overall region can differ from each other considerably [1,5,6].

Various human activities threaten karst environments throughout Southeast Asia. Logging and agriculture can lead to dramatic changes in these habitats by increasing solar radiation influx and desiccation [4,7]. Such changes can alter species communities, for example by causing the decline of less drought tolerant taxa [7]. The extraction of limestone for the production of construction materials, such as concrete, is particularly destructive and poses a major threat to many Southeast Asian karst habitats [4]. In Vietnam, for instance, where approximately one fifth of the territory is covered by limestone karst [8], the cement industry is continuously growing and more than doubled its yearly production between 2005 and 2015 to 67,427 tons [9,10]. The destruction of entire karst habitats can result in the loss of unique species communities and in the extinction of site-endemic taxa [7,11].

Compared to other Southeast Asian countries, Vietnam still remains among the least studied. Even though various researchers have examined the country’s rich and unique biodiversity throughout the nineteenth and early twentieth century, only in the 1990s, after several decades of neglect due to war and political isolation, did Vietnam become the focus of intensive research efforts again [12,13]. During the last few decades, numerous new taxa have been found in Vietnam, among them species of plants, fungi, arthropods, molluscs, amphibians, squamates and birds (e.g. [14–20]), and even larger mammals, such as the saola (*Pseudoryx nghetinhensis*) and the Truong Son muntjac (*Muntiacus truongsonensis*) [21,22]. Many new species are still expected to be discovered. The species diversity of vascular plants in Vietnam, for example, is estimated to be 13,000, while only 8,000 species had been identified until the mid-2000s [12]. In order to preserve the country’s rich biodiversity, a number of protected areas, most importantly national parks and nature reserves, have been established across Vietnam [13]. Although species inventories of certain plant and animal groups have been made for many of these areas (e.g. [23–27]), their biodiversity is still far from being thoroughly explored.

Species richness and endemism often form the basis for planning and managing protected areas [28,29]. Such species-focused conservation approaches, however, strongly rely on correct species identification and the presence of undetected cryptic species can be highly problematic (here, we follow the definition of cryptic species by Bickford et al. [29], who consider “two or more species to be ‘cryptic’ if they are, or have been, classified as a single nominal species because they are at least superficially morphologically indistinguishable”). If cryptic species are not recognised, the species richness of areas can be underestimated, species distribution ranges overestimated and endemic species overlooked. A lack of knowledge about cryptic species and their distribution can lead to wrong decisions when planning the extent of protected areas and to inadequate conservation measures. Rescue attempts can have harmful consequences when heterospecific individuals are unwittingly transferred to another area in order to increase the local population density of a species (see e.g. [30]). In captive breeding programs, unrecognised cryptic taxa can result in unwanted hybridisations between closely related species [30,31]. If knowledge about species’ biology is based on a mixture of different species, their actual requirements remain unknown, which can be problematic, for example, when applying targeted conservation strategies (e.g. [32]).

Cryptic species can evolve if selection pressures promote morphological stasis or lead to convergent evolution of a certain morphological character set [29,33]. Insular habitats, such as oceanic islands, springs, caves or lakes, are assumed to be prone to the allopatric evolution of cryptic species. Prime examples for cryptic species as a result of morphological stasis include hydrobiid snails (*Pyrgulopsis*) in western North American springs, where molecular diversification but little morphological differentiation has been observed [34], and the morphologically similar lineages of *Tropheus* cichlids in Lake Tanganyika, which presumably evolved into different species due to past lake level fluctuations [35]. Cryptic species resulting from convergent evolution have been found among Greater Antilles *Anolis* lizards, which have developed certain morphological characters independently on different islands [36], and African rift lake cichlids, where only distantly related lineages from Lake Tanganyika and Malawi resemble each other in morphology [37]. Due to their insular character, Vietnam’s limestone karsts represent an environment with a high potential to promote the evolution of cryptic species. Knowledge on cryptic diversity among Vietnam’s limestone-associated biota, however, remains limited.

Although the detection rate of cryptic species has increased rapidly over the last few decades due to more efficient molecular tools, thorough species delimitation and formal description of new species are often omitted [38]. These are, however, crucial steps to make cryptic taxa visible and protectable for most conservation programs. Species conservation in quickly developing and highly biodiverse countries, such as Vietnam, is a “race against time”. Effective conservation planning thus requires efficient methods to discover and describe biodiversity [39]. The use of molecular phylogenetics based on DNA sequence data from a number of standard gene fragments has been proven to provide important evidence for the detection and further examination of previously overlooked, cryptic diversity [29,40–42]. In addition, DNA-based species delimitation methods and geometric morphometrics can represent efficient tools for unravelling cryptic taxa [43,44].

The present study focuses on a potential case of cryptic diversity in limestone-associated organisms in Vietnam. Among the country’s highly diverse fauna of karst-inhabiting land snails [45], the genus *Cyclophorus* (Caenogastropoda, Cyclophoridae) belongs to the most conspicuous groups. *Cyclophorus* spp. typically occur in high abundances in limestone habitats and are, in keeping with probably the majority of tropical land snails that inhabit limestone karst [46], also present in adjacent non-limestone habitats, although in much lower abundances (K.C.M. and P.V. von Oheimb, pers. observation; see also [1] for the Malay Peninsula). Many *Cyclophorus* species differ considerably from each other in shell morphology and have relatively small distribution ranges [6,47]. However, representatives of this genus with a very similar shell morphology, to which we refer as the “widespread morphotype” in the present study, have been reported from large parts of Vietnam (including a number of national parks and nature reserves). The shell of the widespread morphotype is of medium size, generally rounded in shape and has a circular aperture. Even though researchers from the late nineteenth and early twentieth century described several *Cyclophorus* species and subspecies with such a shell morphology from Vietnam (see [48,49]), most of these names are rarely used, presumably because the few characters that they have been based on (primarily size, shape and shell colouration), are not sufficient for an unambiguous identification. Instead, snails of the widespread morphotype typically appear in regional faunistic studies and checklists as *Cyclophorus fulguratus* (Pfeiffer, 1854) [50] or *Cyclophorus volvulus* (Müller, 1774) [51] (e.g. [25,52–54]). The names *C*. *fulguratus* and *C*. *volvulus* are also in use for morphologically similar members of the genus from other Asian countries [55–59]. The molecular phylogeny of Oheimb et al. [6], however, recently provided evidence that snails of the widespread morphotype from different limestone karst areas in northern Vietnam belong to several distantly related *Cyclophorus* clades with none of them being closely related to *C*. *fulguratus* (*sensu* [55]) and *C*. *volvulus* (*sensu* [59]) from Thailand.

Given the potential of Vietnam’s insular limestone karsts to promote the evolution of cryptic species and the need for a clarification of species boundaries among representatives of the genus *Cyclophorus* of the widespread morphotype, the two major objectives of the present study were (A) to examine the diversity and evolutionary history of the widespread morphotype in Vietnam based on molecular phylogenetics, and (B) to identify and characterise individual species sharing this morphotype and to formally describe new species found.

## Materials and methods

### Material studied

In this study, a total of 182 *Cyclophorus* samples from Vietnam were examined (S1 Table). This material is part of the land snail collections of the Natural History Museum in London, which currently holds one of the largest scientific collections of Vietnamese *Cyclophorus* spp. [6]. The *Cyclophorus* material in the Natural History Museum’s collections comprises both, ethanol-preserved specimens and dry shells, partly with separate ethanol-preserved frozen tissue samples and/or viable cell preparations. The studied material was obtained during a number of field trips to Vietnam in the 2000s and 2010s, some of them conducted as part of a Darwin Initiative project [60]. All material examined in this study was from georeferenced sampling localities with the exception of one group of samples, for which such data were not available. These specimens, which were obtained from a local market in the Vietnamese province of Hoa Binh, had been collected in the surroundings of the market according to the merchant; their exact sampling locality, however, remained unknown (see S1 Table for details).

All specimens with a generally rounded shell, a circular aperture, a medium high conical spire, a shell height ≥23 and ≤31 mm, and a shell breadth ≥28 and ≤35 mm were classified as the “widespread morphotype” (S1 Table; shell height and breadth follow [61]). Specimens that differed from the widespread morphotype only moderately in terms of shell shape and/or shell size (i.e. shell height ≥21 and <23 mm, or >31 and ≤33 mm, and/or shell breadth ≥26 and <28 mm, or >35 and ≤37 mm) were classified as “slightly deviating”. All specimens that were clearly different from the widespread morphotype in terms of shell shape and/or size (i.e. shell height <21 or >33 mm, and/or shell breadth <26 or >37 mm) were classified as “deviating”.

### Molecular phylogenetic analysis

For the molecular dataset new sequence data from 49 Vietnamese *Cyclophorus* specimens, including 30 individuals of the widespread morphotype as well as 8 with a slightly deviating and 11 with a deviating shell morphology, were generated (S1 Table). DNA extraction [62] was carried out using ethanol-preserved foot tissue of individual snails. A 658-bp fragment of the mitochondrial cytochrome *c* oxidase subunit I (COI) gene was amplified using either LCO1490 [63], PF372 [64] or Cyc-F01 (5’-CTTCGACGAATCATAAAGATATTGG-3’) as forward primer and HCO2198 [63] as reverse primer. For the amplification of a 505–510-bp fragment of the mitochondrial 16S rRNA gene, the forward primer 16Sar-L and the reverse primer 16Sbr-H (both [65]) were used. Two different fragments of the nuclear 28S rRNA gene were amplified; either a 585–589-bp fragment using the forward primer 28SF4 and the reverse primer 28SR5 (both [66]), or a 459–462-bp fragment using 28SF4 and the reverse primer LSU-4 [67]. Details about the primer combinations used for individual samples are given in S1 Table. PCR conditions for COI were: 120 s at 94°C, 36 cycles (30 s at 94°C, 120 s at 42°C, 120 s at 72°C), and 300 s at 72°C [47]. PCR conditions for 16S and 28S were: 300 s at 94°C, 29 cycles (30 s at 95°C, 30 s at 52°C, 30 s at 72°C), and 300 s at 72°C [6]. Bi-directional DNA sequencing was carried out with an AB 3730XL DNA Analyzer (Applied Biosystems, Waltham, USA) using the BigDye Terminator v3.1 Cycle Sequencing Kit (Applied Biosystems, Waltham, USA). Heterozygous insertions or deletions in the 28S dataset were resolved using Mixed Sequence Reader [68]. All heterozygous sites in the 28S dataset (<0.1% of all positions) were regarded as missing data. All new sequences have been deposited at GenBank (S1 Table).

Sequence data from further samples, 82 *Cyclophorus* specimens and three specimens of outgroup taxa, were taken from the literature [6,47,55] (S1 and S2 Tables). The Vietnamese *Cyclophorus* specimens among these samples included 8 of the widespread morphotype as well as 5 with a slightly deviating and 39 with a deviating shell morphology (the two Vietnamese samples from Nantarat et al. [47] were classified as deviating based on the images in their paper). The protein-coding COI sequences were aligned using CLUSTAL W [69] implemented in BioEdit version 7.2.6 [70]. For the alignment of the non-coding 16S and 28S sequences, the Q-INS-i strategy [71] in MAFFT version 7 [72], which considers the secondary structure of RNA, was used with default settings. Sequence data from the three genes were combined and identical sequences were removed from the primary alignment of 134 sequences, yielding a total of 114 unique haplotypes. To test for substitution saturation of the COI (codon positions 1/2 and 3 separately), 16S and 28S datasets, the entropy-based method of Xia et al. [73] implemented in DAMBE 7.0.1 [74,75] was used. The test revealed little saturation for all datasets under the assumption of a symmetrical tree. Under the assumption of an asymmetrical tree, COI codon position 3 was rated as useless, whereas COI codon positions 1/2 as well as 16S and 28S showed little saturation.

For a phylogenetic reconstruction based on the three sequenced gene fragments, MrBayes 3.2.6 [76] was used on the CIPRES Science Gateway V. 3.3 [77]. In order to find optimal substitution models, the corrected Akaike information criterion in jModelTest 2.1.9 [78] was applied. The suggested models were GTR+I+G for COI, HKY+I+G for 16S and TIM1+I+G for 28S. As TIM1+I+G is not implemented in MrBayes, GTR+I+G was used instead as the closest over-parameterised model [79]. Two Markov chain Monte Carlo (MCMC) simulations with one cold and three heated chains were executed in parallel for 5,000,000 generations (50,000 trees). The runs were checked with Tracer v1.7.1 [80] and showed high effective sample size values (> 300 for all parameters) and smooth frequency plots. A consensus tree was calculated from the sampled trees, while discarding the first 10% as burn-in.

### Species delimitation

In order to delimit putative species-level clades of *Cyclophorus* from Vietnam that include specimens of the widespread morphotype, two different approaches were used, Automatic Barcode Gap Discovery (ABGD) [81], which is based on pairwise genetic distances, and the tree-based Bayesian implementation of the Poisson tree processes model (bPTP) [82]. For ABGD, the same COI dataset that has been used for the Bayesian phylogeny was analysed. The respective COI fragment corresponds to that suggested by Hebert et al. [83] for DNA barcoding. Default settings were used in the ABGD web-interface except of the relative gap width (*X*), which was set to 1. For bPTP, a single-locus molecular phylogeny is required [82]. Therefore, an additional phylogenetic tree based only on the mitochondrial COI and 16S datasets from the main analysis was generated in MrBayes using the same methodology as described above (S1 Fig). The bPTP analysis was run on the bPTP server for 500,000 MCMC generations with 10% burn-in. All outgroup taxa were removed for the bPTP analysis.

To gain shell morphological data, all specimens were examined under a stereo microscope and measured with a dial caliper or based on a standardised digital photograph (all specimens individually described in the taxonomic section were caliper-measured). Soft body and radula characters were not investigated given their uncertain diagnostic value within the genus (see [84] for comparisons of the reproductive system and [55, 59] for comparisons of the radula among *Cyclophorus* species).

Based on the results of AGBD, bPTP, the topology of the phylogenetic tree, as well as on distributional and shell morphological criteria, all Vietnamese *Cyclophorus* clades that included specimens of the widespread morphotype were grouped into species. The delimited species were then compared with already described taxa based on original literature and type material; where it was not possible to match a species with any known taxon, we described it as a new species (with one exception; see Results). All *Cyclophorus* specimens from Vietnam that did not belong to the herein delimited species were referred to as “*Cyclophorus* sp.” as a revision of the respective taxa was beyond the scope of the present study.

### Morphological characterisation of delimited *Cyclophorus* species

In order to further morphologically characterise the delimited *Cyclophorus* species, geometric morphometrics was used. The morphological dataset included all individuals, which were sequenced in this study or in Oheimb et al. [6] and which belonged to the herein delimited species (see Results). In addition, assumed conspecifics of these individuals, which were collected at the same place or up to 5 km from that place, while the respective sampling locality belonged to the same karst region and was not geographically separated by potential dispersal impediments, such as rivers, were included in the dataset. Karst regions and potential dispersal impediments were determined based on Dang [85] and on satellite imagery available via Google Earth Pro 7.3.1.4507 (Google Inc., Mountain View, USA). Altogether, 139 specimens of both sexes (5 to 20 individuals per species, ethanol-preserved or dry material), were used (i.e. all specimens of the delimited species in S1 Table). The samples included 92 individuals of the widespread morphotype as well as 26 with a slightly deviating and 21 with a deviating shell morphology. All specimens were fully-grown (determined by their thickened lip). We primarily used individuals with an intact shell; only in some specimens, pieces of the aperture were broken off and in very few specimens, pieces of the early whorls, which include the protoconch, were missing.

Individual shells were digitally photographed in standardised orientation (apertural view, the apex-umbilicus axis parallel to the ground). Shell outlines were traced using Corel Draw X7 (Corel Corporation, Ottawa, Canada) and resulting images were compiled using tpsUtil version 1.70 [86]. Subsequently, 200 semi-landmarks along each outline (following [87]) were generated in tpsDig2 version 2.30 [88]. The first landmark marked the widest point of the aperture on the right side of an outline. In cases where pieces of the shell were missing, the outlines were interpolated. To examine differences in shell shape among the delimited *Cyclophorus* species, a Procrustes transformation, which removes variation in size, orientation and position, and a subsequent canonical variate analysis (CVA) were performed in MorphoJ version 1.06d [89]. To examine differences in shell size among the delimited *Cyclophorus* species (as well as among selected sub-groups; see Results), the area of the polygon spanned by all semi-landmarks was calculated for each specimen using R version 3.5.0 [90] with the package geometry 0.3–6 [91]. We chose this area as it is more sensitive to overall changes in shell dimensions than shell height or breadth alone.

### Nomenclatural acts

The electronic edition of this article conforms to the requirements of the amended International Code of Zoological Nomenclature, and hence the new names contained herein are available under that Code from the electronic edition of this article. This published work and the nomenclatural acts it contains have been registered in ZooBank, the online registration system for the ICZN. The ZooBank LSIDs (Life Science Identifiers) can be resolved and the associated information viewed through any standard web browser by appending the LSID to the prefix “http://zoobank.org/”. The LSID for this publication is: urn:lsid:zoobank.org:pub:A3453015-CC61-404A-BEBD-03B2A1DB3510. The electronic edition of this work was published in a journal with an ISSN, and has been archived and is available from the following digital repositories: PubMed Central, LOCKSS.

## Results

**Fig 1:**
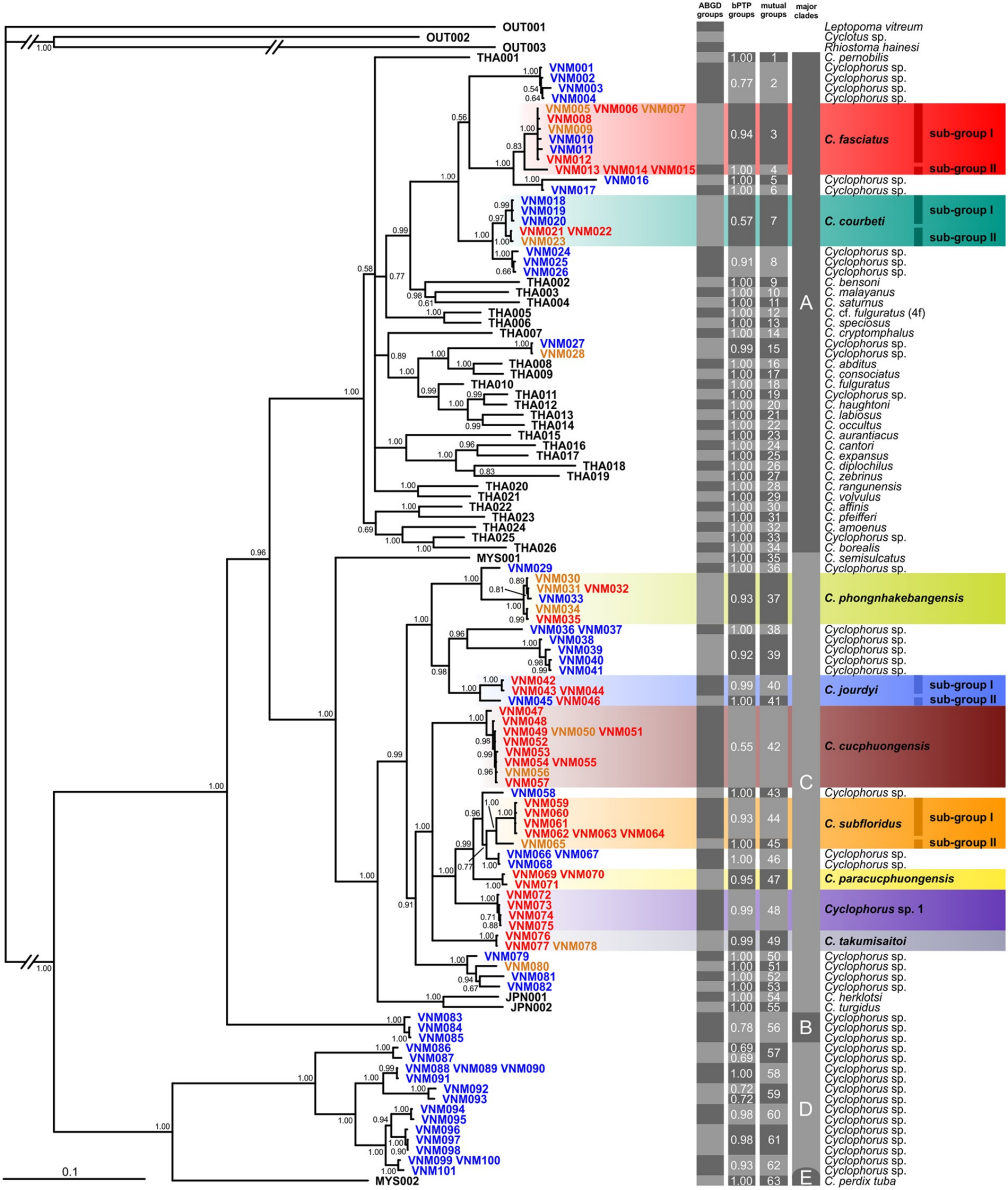
Consensus Bayesian phylogeny of *Cyclophorus* spp. including results of Automatic Barcode Gap Discovery (ABGD) and the Bayesian implementation of the Poisson tree processes model (bPTP). The phylogeny is based on sequence data from the COI, 16S and 28S genes. Bayesian posterior probabilities are provided at the respective nodes. The scale bar indicates the number of substitutions per site according to the applied model of sequence evolution. Specimen codes (S1 and S2 Tables) refer to outgroups (“OUT”) and respective *Cyclophorus* sampling localities (“VNM”: Vietnam, “THA”: Thailand, “MYS”: Malaysia, “JPN”: Japan). The tree was rooted with the outgroup *Leptopoma vitreum* (OUT001). Specimen codes of individuals with identical sequence data are given at the same branch tip. The specimen codes of Vietnamese *Cyclophorus* specimens are colour-coded according to shell morphology: specimens classified as the widespread morphotype are marked in red, specimens classified as slightly deviating in orange and specimens classified as deviating in blue. Putative species-level clades delimited via ABGD (“ABGD groups”) and bPTP (“bPTP groups”; support values are given for each clade), and clades confirmed by both approaches (“mutual groups”; individually numbered) are shown. Major clades were named following Oheimb et al. [6]. The nine species that include Vietnamese specimens of the widespread morphotype (and respective sub-groups) are highlighted. Species names for Vietnamese *Cyclophorus* samples follow this study; species names for other samples were taken from the literature [47,55, 59] (except in cases of conflict, see taxonomic section).

The Bayesian phylogeny (Fig 1) revealed five major clades of *Cyclophorus* spp. (A–E; named following [6]). These clades were supported with Bayesian posterior probabilities (BPP) of 1.00, except of major clade E, which comprised only a single haplotype. The Vietnamese *Cyclophorus* specimens of the widespread morphotype did not form a single monophyletic group but clustered into several different clades within major clade A and C.

In the ABGD analysis, the number of groups for the initial and recursive partitions matched at a prior limit to intraspecific divergence (*P*) of 0.00464. For this value, the analysis revealed 63 putative species-level clades of *Cyclophorus* (and three further outgroup clades; Fig 1). The bPTP analysis resulted in 65 putative species-level clades of *Cyclophorus* in the most supported partition; most of them with good bPTP support values (Fig 1). A total of 63 putative species-level clades of *Cyclophorus* were “mutual groups”, i.e. they were confirmed by both approaches (Fig 1). The ABGD and bPTP analyses consistently delimited eleven putative species-level clades (mutual groups 3, 4, 7, 37, 40, 41, 42, 44, 47, 48 and 49; BPP ≥ 0.97 or single haplotypes), which included specimens of the widespread morphotype, and three clades (mutual groups 15, 45 and 51; BPP = 1.00 or single haplotypes), which included specimens classified as slightly deviating but none of the widespread morphotype. Two of the latter clades (mutual groups 15 and 51) were not included in the following steps, as we had no evidence for the presence of specimens of the widespread morphotype in these groups. The third one (mutual group 45), however, was regarded as a clade that develops the widespread morphotype because shells of assumed conspecifics of the sequenced specimen with this morphology were found at the same sampling locality (see S1 Table). In three cases, ABGD/bPTP-delimited clades that develop the widespread morphotype were relatively closely related sister groups (mutual groups 3 and 4 (BPP = 0.83), 40 and 41 (BPP = 1.00), and 44 and 45 (BPP = 1.00); see Fig 1) with neighbouring distribution ranges and a similar shell morphology. We therefore regarded each of these sister group pairs as a single species (each with two sub-groups; see taxonomic section for details). In one case, two morphologically clearly distinct sub-clades formed one clade delimited by ABGD and bPTP (mutual group 7), with specimens of the widespread morphotype being present in only one of them (sub-group II) but not in the other (sub-group I), which included significantly larger individuals (see taxonomic section for details). Given the results of ABGD and bPTP, their very close phylogenetic relationship, and the neighbouring distribution ranges, these sub-clades were regarded as a single species. Thus, a total of nine different *Cyclophorus* species, which included specimens of the widespread morphotype, were delimited. Four of these species could be matched with already described taxa based on shell morphology and geographic distribution (*Cyclophorus courbeti* Ancey, 1888 [92]; *Cyclophorus fasciatus* Kobelt, 1908 [48]; *Cyclophorus jourdyi* Morlet, 1886 [93]; *Cyclophorus subfloridus* Ancey, 1888 [92]), four were described as new species (*Cyclophorus cucphuongensis* Oheimb, sp. nov.; *Cyclophorus paracucphuongensis* Oheimb, sp. nov.; *Cyclophorus phongnhakebangensis* Oheimb, sp. nov.; *Cyclophorus takumisaitoi* Hirano, sp. nov.), and one was left undescribed as it was only found in the material from the market and the exact sampling locality remained unknown (*Cyclophorus* sp. 1) (see taxonomic section). Two of the delimited species, *C*. *courbeti* and *C*. *fasciatus*, were part of major clade A, and seven of them, *C*. *jourdyi*, *C*. *subfloridus*, *C*. *cucphuongensis*, *C*. *paracucphuongensis*, *C*. *phongnhakebangensis*, *C*. *takumisaitoi* and *Cyclophorus* sp. 1, were part of major clade C. The respective sister groups of all species that included specimens of the widespread morphotype contained ABGD/bPTP-delimited clades that only included specimens with a deviating shell morphology.

**Fig 2:**
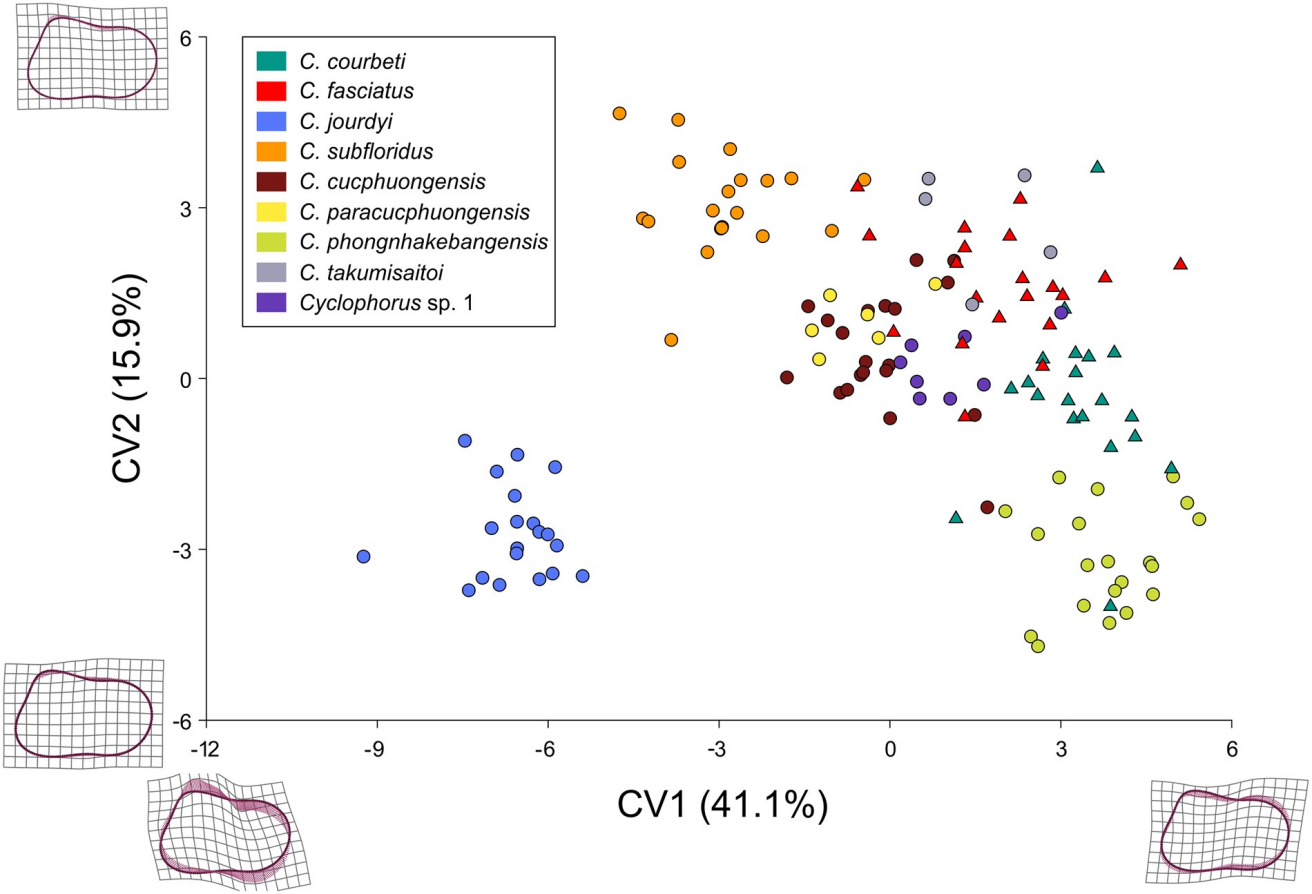
Canonical variate analysis (CVA) of shell shape data. The delimited *Cyclophorus* species are colour-coded. Data points of individuals belonging to major clade A are shown as triangles, data points of individuals belonging to major clade C are shown as circles. The figures at the axes represent the transformation from the overall mean shape at -12 and 6 (CV1), and at -6 and 6 (CV2).

The first two axes of the CVA of the nine delimited species’ shell shape explained 41.1% (CV1) and 15.9% (CV2) of the total variance (Fig 2). Lower CV1 values were associated with a less compressed and higher values with a more compressed shell. Changes in CV2 reflected more subtle differences and were associated with changes in the height of the spire and the angle between the aperture and the body whorl. Even though the CVA separated some species along CV1 and/or CV2 to a certain extent, almost all species were either partly or completely overlapping in morphospace with at least one other species; and species belonging to major clade A were largely overlapping with species from major clade C. *Cyclophorus jourdyi* was the only species in the analysis, which did not overlap in morphospace with any other species.

**Fig 3:**
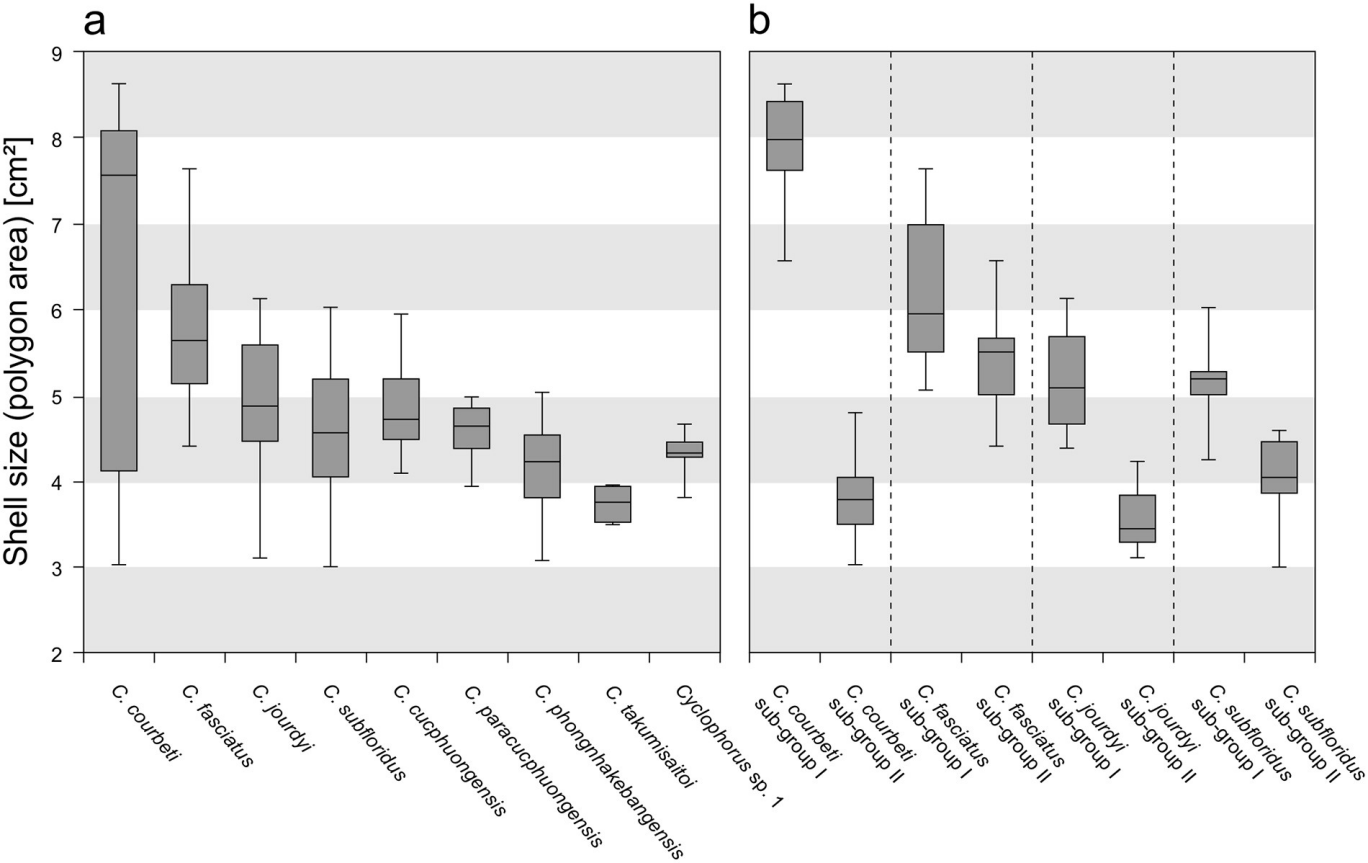
Boxplot diagram of shell size. (a) Shell size (defined as the area of the polygon spanned by all generated semi-landmarks) of delimited *Cyclophorus* species and (b) of sub-groups. Boxes encompass the second and the third quartiles. Horizontal lines within each box represent the median. Top and bottom whiskers show the largest and smallest data point.

The examination of shell size (defined as the area of the polygon spanned by all generated semi-landmarks) showed that, while medians and ranges differed between species, almost all of them showed an overlap with at least some individuals from all other species (Fig 3a). The only exception was *C*. *takumisaitoi*, where no individuals overlapped in size with individuals of *C*. *fasciatus* and *C*. *cucphuongensis* (but note that *C*. *takumisaitoi* had the lowest number of specimens in our dataset; n = 5). The range in shell size in *C*. *courbeti* was considerably wider than in all other species.

The examination of the sub-groups within species revealed some differences in shell size, while the most pronounced difference was found between the two sub-groups of *C*. *courbeti*, with sub-group I having considerably larger shells than sub-group II (Fig 3b).

**Fig 4:**
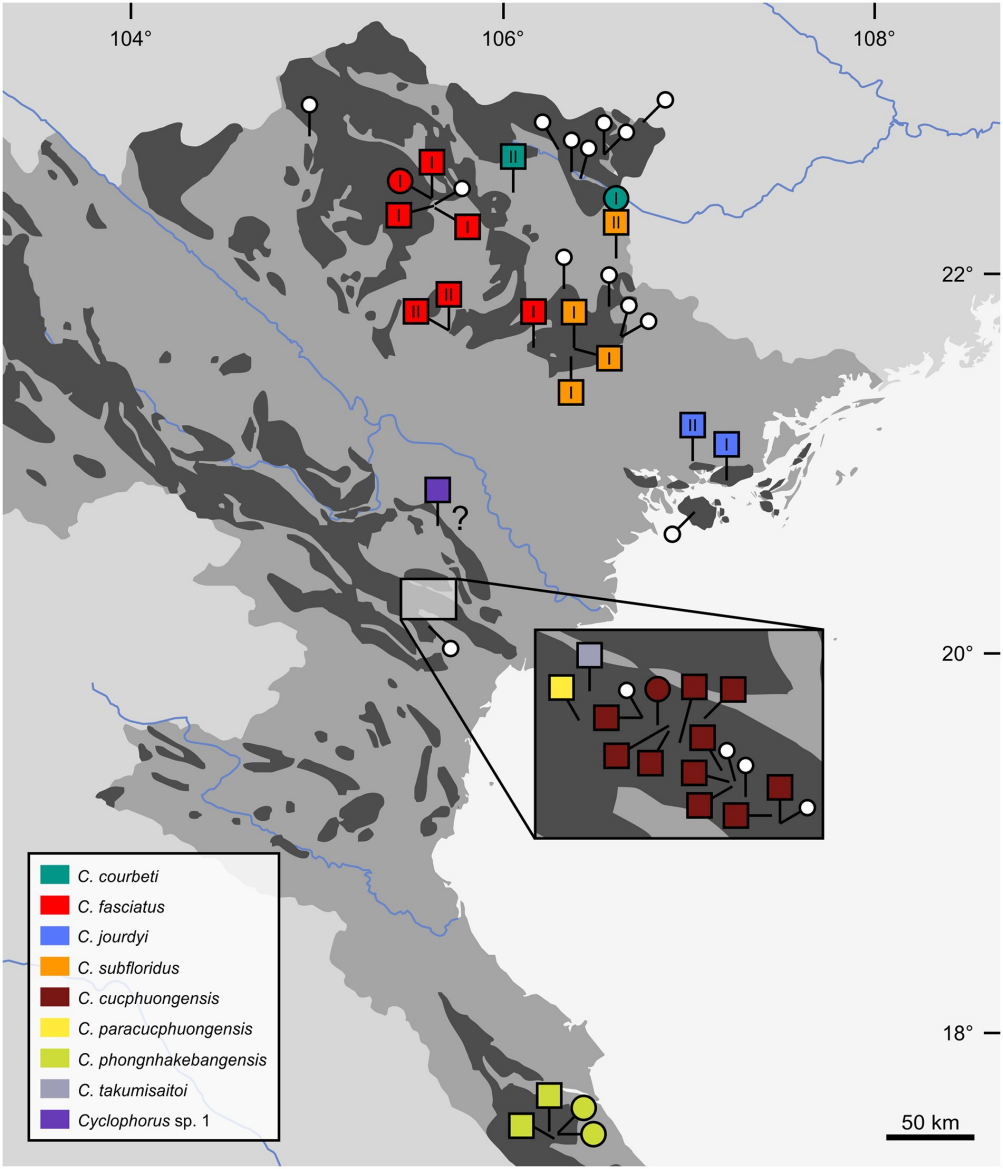
Sampling localities of *Cyclophorus* spp. in northern Vietnam. Markers with large symbols refer to sampling localities of *Cyclophorus* species that develop the widespread morphotype (colour-coded according to species); squares indicate that individuals of the widespread morphotype were present in the studied material from the respective localities and circles indicate that such individuals were not present. Roman numerals refer to sub-groups of individual species. The question mark indicates that the exact sampling locality is unknown (the respective marker shows the location of the market stall where the material of *Cyclophorus* sp. 1 was obtained). Markers with small white circles refer to localities, from where only *Cyclophorus* species that have not been found to develop the widespread morphotype were studied. The map shows all sampling localities of *Cyclophorus* specimens from Vietnam from S1 and S2 Tables, with the exception of three localities that are outside the map section (all of them only with species that have not been found to develop the widespread morphotype). Sampling localities for samples from Nantarat et al. [47] were derived from the map in their paper. The distribution of limestone karst (according to [85]) is shown in dark grey.

The geographical distribution of the delimited species was typically allopatric (Fig 4). This pattern was sometimes associated with interjacent geographical features, such as non-karst areas. Respective features, however, were not always present, such as for *C*. *cucphuongensis* and *C*. *takumisaitoi*, which were found to occur in relatively close proximity in Cuc Phuong National Park (see also S1 Table). The studied specimens of the widespread morphotype from one locality belonged always to only one species. At a single locality, two of the delimited species that develop the widespread morphotype, *C*. *courbeti* and *C*. *subfloridus*, were found to occur sympatrically. At this locality, the studied specimens of *C*. *courbeti* (sub-group I) were significantly larger than the widespread morphotype (because of their larger size, all specimens were classified as deviating with the exception of one smaller specimen that was classified as slightly deviating) and most studied individuals of the co-occurring *C*. *subfloridus* (belonging to sub-group II of this species) had a laterally more compressed shell and a higher spire than the widespread morphotype (they were thus classified as slightly deviating).

## Discussion

The detection of nine different *Cyclophorus* species in northern Vietnam that develop the widespread morphotype, several of them previously unknown to science, revealed a remarkable cryptic diversity. Given that these morphologically similar taxa clustered into two different major clades that also contained various morphologically deviating lineages, processes of convergent evolution were probably involved in their origin. Morphological stasis, however, could also have played a role, particularly in cases of relatively closely related species. The delimited species evolved presumably in allopatry under similar selection pressures among the region’s scattered limestone karsts. Allopatric evolution among isolated karst habitats has also been suggested for limestone-associated taxa from southern China, such as the gesneriad plant species *Primulina eburnea*, where a strong isolation between karst populations was found [3], and cave spiders of the genus *Nesticella*, where multiple independent colonisations of isolated karst caves have been assumed [94].

The strictly allopatric distribution of the widespread morphotype could result from interspecific competition, which prevents the co-occurrence of more than one species with this morphology, as a more similar shell morphology can be associated with a more similar ecology in land snails (e.g. [95,96]). Even though the ecology of *Cyclophorus* spp. is generally not well known and the extent of niche overlap between individual species remains unclear, the importance of interspecific competition for the assembly of *Cyclophorus* communities in Vietnam was already stressed by Oheimb et al. [6], who found phylogenetic and morphological overdispersion to be common among communities from different karst areas.

The level of variation in terms of shell size, shape and other examined shell characters was relatively high within the delimited *Cyclophorus* species compared to the differences among them, where a high degree of morphological overlap was found (see also taxonomic section). This could explain why they were often regarded as conspecific. The actual level of intraspecific morphological variation and the degree of interspecific overlap are probably even higher, as some taxa in our study were represented by only relatively low individual numbers and as the geographical distribution of the species was likely not fully covered. The only species that did not show an overlap with individuals from any of the other species in the CVA was *C*. *jourdyi*. This species, which was found at the coastal margin of northeastern Vietnam, differed from the other delimited species mainly by its comparatively higher shell. Its shell shape may reflect slightly deviating selective pressures in its distribution range.

Even though three of the delimited species, *C*. *fasciatus*, *C*. *jourdyi* and *C*. *subfloridus*, were further separated by ABGD and bPTP, each into two putative species-level sub-groups, we believe that the current evidence does not justify a further splitting of these taxa. However, given the phylogenetic distance between the sub-groups and the slight sub-group-specific differences in shell size, shape and other shell characters, ongoing processes of speciation, presumably due to geographic isolation and differing selection pressures could be involved. *Cyclophorus courbeti* was rated as a single putative species-level clade by ABGD and bPTP. Here, two phylogenetically closely related sub-groups, each of them found at a single locality (approx. 68 km apart), were morphologically clearly distinct. While sub-group II of *C*. *courbeti* included specimens of the widespread morphotype, specimens of sub-group I were significantly larger. The latter sub-group was found sympatrically with *C*. *subfloridus*, which was the only case in our study, where two *Cyclophorus* species that develop the widespread morphotype were co-occurring. At the respective locality, both species differed considerably from each other in shell morphology, which might represent a case of character displacement, where a shift in morphology has led to a decrease in competition between the two species and hence allows their co-occurrence. It remains unclear whether the observed morphological differences between the closely related sub-groups of *C*. *courbeti* are genetically fixed or result from phenotypic plasticity. Future studies should thus focus on the ability and performance of *Cyclophorus* spp. to morphologically respond to different environments.

Given the presence of multiple *Cyclophorus* species with a highly similar shell morphology in Vietnam (a pattern also found in other regions of Southeast Asia [55, 59]) and the comparatively high level of intraspecific variation within certain species, we emphasise that it is typically not sufficient to describe new recent species in this genus based solely on shell characters. We believe that such descriptions, which are still practiced today, can even hinder and delay future taxonomic work. The present study highlights the usefulness of DNA sequence data as a basis for the delimitation and description of *Cyclophorus* species. In addition to molecular data, the exact geographic location can be very informative for identifying morphologically similar, but typically allopatrically distributed species. Both data should be the minimum information provided in future species descriptions.

Even though the studied *Cyclophorus* morphotype is widespread across Vietnam, the individual species appear to be restricted to relatively small geographical areas. None of the species that develop the widespread morphotype was found in more than one of the examined national parks and nature reserves (see S1 Table). While the diversity of medium-sized *Cyclophorus* species in the studied parts of northern Vietnam is remarkably high, morphologically similar representatives of the genus have also been reported from other areas of the country [49,53,97]. We thus expect the total number of *Cyclophorus* species in Vietnam that develop the widespread morphotype to be even higher. Given the island-like character of Vietnam’s limestone karsts and their potential to generate cryptic diversity, it is likely that so far undiscovered morphologically cryptic taxa with relatively small distribution ranges have evolved in other groups of limestone-associated organisms as well. This might especially apply to those taxa, for which only few morphological characters for distinguishing species have been established or which include species that are assumed to have wide distribution ranges. Given that *Cyclophorus* spp. also occur in non-limestone habitats, a strict dependency on limestone is probably not a requirement for the evolution of cryptic diversity among limestone-associated taxa. Undetected cryptic species of karst organisms can not only result in an underestimation of an area’s total biodiversity, but also of the biotic uniqueness of individual karst regions. Notably, cryptic species inhabiting neighbouring regions might be prone to be misidentified as being conspecific.

Given the findings of the present study and the potential that Vietnam’s threatened karst habitats contain many more cryptic species from various groups of organisms, we argue for a systematic detection and description of this diversity. This is needed to assess the degree of biotic uniqueness and endemism, which should guide decisions about limestone conservation and extraction.

## Taxonomic section

This section includes characterisations of all nine morphologically similar *Cyclophorus* species, which have been delimited in this study (see Results). In an effort to match these species with previously described taxa, we carried out a comprehensive overview of all the potentially available names. This overview included all formally described *Cyclophorus* taxa with type localities adjacent to the distribution ranges of the delimited species (i.e. Vietnam, Laos and adjacent regions of China) based on Kobelt [48,98] and Do & Do [49], references therein, and on a survey of the Zoological Record database and additional online resources (Global Biodiversity Information Facility, Worldwide Mollusc Species Data Base, MolluscaBase, Google Scholar). We then excluded all taxa that were clearly different in shell size and shape from the nine delimited species based on relevant type material and/or original descriptions. We also excluded all taxa that have been described from regions that lie well outside the distribution ranges of the delimited species. For all remaining taxa, we carefully compared type material (if available; see below for details) and original descriptions with our specimens. Where it was not possible to match a species with any previously described taxon, we described it as a new species (with the exception of *Cyclophorus* sp. 1; see below).

The species characterisations provided here are based on geographical, shell morphological and DNA sequence data, and these elements vary in their diagnostic value. Geographic information is important for discriminating between the different characterised *Cyclophorus* species. However, it has to be kept in mind that the exact extent of the species’ distribution ranges is not known and that morphologically similar individuals of different species can occur in close proximity. While co-occurring *Cyclophorus* taxa have been found to differ considerably in shell size and/or shape ([6]; see therein for *Cyclophorus* communities that include *C*. *fasciatus*, *C*. *jourdyi*, *C*. *cucphuongensis* and *C*. *phongnhakebangensis*), the shell morphology of the species characterised here is, on its own, often not sufficient for reliable species identification. The main reason for this is the broad interspecific overlap and the comparatively high level of intraspecific variation in shell characters (see also individual conchological descriptions below); for instance, not a single examined shell character that was common to all studied individuals of one species was unique to this species. Although we provide several morphological characters that may aid discrimination between some of the species, the most reliable mode of identification is via DNA sequence data. While ideally the full dataset of DNA sequences used in this study should be utilised for species identification, a set of diagnostic nucleotide substitutions of the COI gene was identified and included in the diagnosis of each species characterised here (see also [99]). Each species-specific set of COI nucleotide substitutions includes characters that were found in all studied individuals of the relevant species, but were absent from the other herein characterised species. Where multiple characters are given for one position, all of them were exclusively found in individuals of the respective species. In cases where herein characterised species were found to have geographically adjacent ranges or to occur sympatrically, we sought to provide morphological characters that enable such species to be differentiated (see remarks for individual species below; note, however, that the number of sampled populations differed among species, see also Fig 4 and S1 Table).

Conchological descriptions were based on all examined material of the respective species (S1 Table). The studied shells of the nine species characterised here had heights ranging from 20.5 to 34.6 mm, breadths ranging from 23.9 to 43.1 mm, and 4.75 to 5.5 whorls (shell height, breadth and number of whorls (rounded to the nearest quarter) follow [61]; the whorl number was estimated in cases where pieces of the early whorls were missing, these estimates are given for individual specimens but have not been included in the conchological descriptions). In all species characterised here, the protoconch (see [100] for observations on embryonic shells of *Cyclophorus jerdoni* (Benson, 1851) [101]) was without sculpture in the beginning, followed by a part with transverse linear elevations (also called striations [100] or ridges [55, 59]). Usually, the space between these elevations slowly increased in growth direction. The teleoconch was, beside more or less pronounced irregular striae, relatively smooth. The shape of the body whorl varied in degree of compression and roundness, while a sub-angular shape was usually accompanied by the presence of a keel along the whorl’s periphery. The shells of most individuals showed a peripheral band that was approx. 2 mm broad and usually dark brown. Directly above the peripheral band, a typically finer, very light brown to white, often disrupted band was positioned. Apart from these bands, shells were variable in colour and pattern. Shell colour ranged from white to different shades of brown, with the darkest shade of brown appearing almost black. Shells were generally darker above the position of the peripheral band and lighter below it. The opercula were flat to slightly concave, thin and typically spiral (irregular opercula, which were sometimes present, could result from damages), and they appeared transparent, dull, slightly reflective or coated with a thin layer of soil (opercula examined under dry conditions). The sex of individuals was determined by the presence or absence of a penis on the right side of the head.

The below mentioned material is housed in the following collections (indicated by the respective registration number prefix): Natural History Museum, London (NHMUK), Senckenberg Forschungsinstitut und Naturmuseum, Frankfurt am Main (SMF), Muséum National d’Histoire Naturelle, Paris (MNHN) and Royal Belgian Institute of Natural Sciences, Brussels (RBINS). Classification follows Egorov & Greke [102] (consistent with [103]). As the limits between the different subgenera described within *Cyclophorus* [102] are generally not well understood [104] and a revision of them was beyond the scope of the present study, we did not classify the species into subgenera.

### Systematics

Family **Cyclophoridae** Gray, 1847 [105]

Subfamily **Cyclophorinae** Gray, 1847 [105]

Genus ***Cyclophorus*** Montfort, 1810 [106]

#### Type species

*Helix volvulus* Müller, 1774 [51] (by original designation)

***Cyclophorus courbeti*** Ancey, 1888 [92]

#### Conchological description

Medium to large-sized *Cyclophorus* species, overall shell shape rounded, aperture typically circular. Shell height 21.1 to 34.6 mm (mean = 30.0 mm), shell breadth 25.8 to 43.1 mm (mean = 36.1 mm) (sub-group I: shell height 31.6 to 34.6 mm (mean = 33.2 mm), shell breadth 36.9 to 43.1 mm (mean = 40.5 mm); sub-group II: shell height 21.1 to 26.7 mm (mean = 24.1 mm), shell breadth 25.8 to 31.4 mm (mean = 28.1 mm)). Number of whorls 4.75 to 5.5 (sub-group I: 5.25 to 5.5; sub-group II: 4.75 to 5.25). Early whorls without bluish colouration. Shape of body whorl usually slightly compressed or uncompressed; usually rounded or sub-angular (sub-group I: usually slightly compressed or uncompressed; usually rounded; sub-group II: slightly compressed or uncompressed; rounded or sub-angular). Lip usually reflected and simple (sub-group I: reflected and simple; sub-group II: reflected and simple, or unreflected and duplicated). Lip colour ivory, white, orange, peach or yellow (sub-group I: ivory, orange, peach, white or yellow; sub-group II: ivory or white). Wing-shaped projection at columellar margin absent. Peripheral band usually dark brown (sub-group I: usually dark brown; sub-group II: dark brown). Colour pattern generally variable. Typical elements of pattern above position of peripheral band: zig-zag stripes, spiral lines and spiral bands in different shades of brown or in white (sub-group I: zig-zag stripes, spiral bands and spiral lines in different shades of brown or in white; sub-group II: zig-zag stripes and spiral lines in different shades of brown). Typical elements of pattern below position of peripheral band: spiral lines, spiral bands and flames in different shades of brown (sub-group I: spiral lines, spiral bands and flames in darker shades of brown; sub-group II: spiral lines, spiral bands and flames in different shades of brown).

#### Displayed specimens

**Fig 5:**
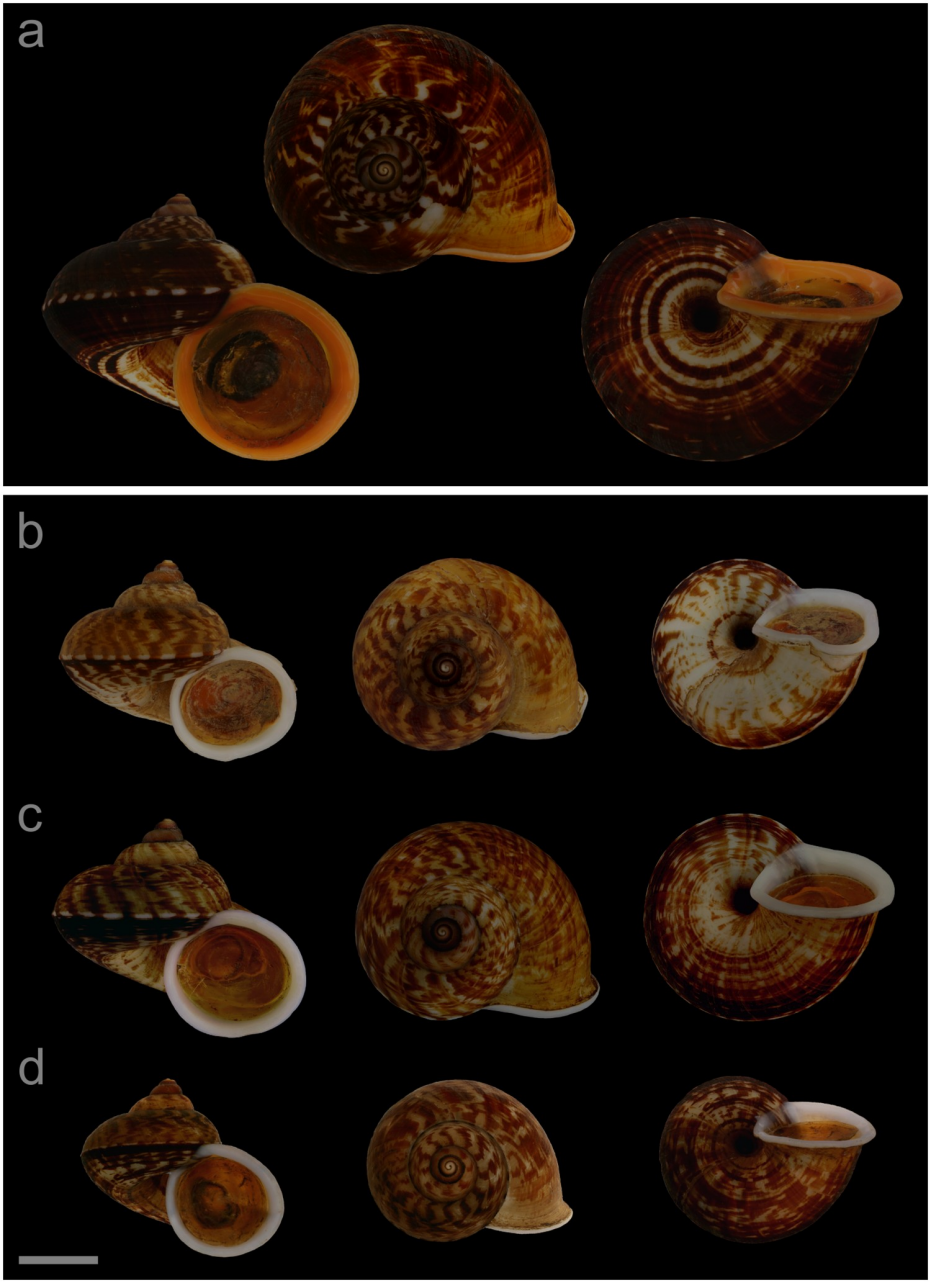
Specimens of *Cyclophorus courbeti* Ancey, 1888 [92]. Front, top and bottom view of (a) VNM019 (sub-group I), (b) VNM022 (sub-group II), (c) VNM021 (sub-group II) and (d) VNM023 (sub-group II). Scale bar: 10 mm.

**VNM019** (Fig 5a; sub-group I), specimen in ethanol; NHMUK 20110591 (ethanol-preserved frozen tissue samples NHMUK Barcode 014041333, NHMUK Barcode 014041350); GenBank accession numbers MN153306 (COI), MN153355 (16S), MN153403 (28S); sex of individual not determined (parts of soft body damaged); shell height 32.2 mm, shell breadth 39.0 mm, number of whorls 5.25. **VNM022** (Fig 5b; sub-group II), specimen in ethanol; NHMUK 20110540/2; GenBank accession numbers MN153309 (COI), MN153358 (16S); female individual; shell height 24.8 mm, shell breadth 29.0 mm, number of whorls 5.25. **VNM021** (Fig 5c; sub-group II), specimen in ethanol; NHMUK 20110540/1 (ethanol-preserved frozen tissue samples NHMUK Barcode 014041331, NHMUK Barcode 014041348); GenBank accession numbers MN153308 (COI), MN153357 (16S), MN153405 (28S); sex of individual not determined (parts of soft body damaged); shell height 26.7 mm, shell breadth 31.4 mm, number of whorls 5.25. **VNM023** (Fig 5d; sub-group II), specimen in ethanol; NHMUK 20110541 (ethanol-preserved frozen tissue samples NHMUK Barcode 014041332, NHMUK Barcode 014041349); GenBank accession numbers MN153310 (COI), MN153359 (16S), MN153406 (28S); sex of individual not determined (parts of soft body damaged); shell height 22.6 mm, shell breadth 26.3 mm, number of whorls 4.75.

#### Locality and sampling data

**VNM019**: Vietnam, Lang Son, Trung Quan commune (22.13137°, 106.58715°); 23 May 2011; leg. Hao Van Luong, Fred Naggs & Sang Van Pham. **VNM022**, **VNM021**, **VNM023**: Vietnam, Bac Kan, Bang Van commune (22.47878°, 106.04597°); 19 May 2011; leg. Jonathan Ablett, Hao Van Luong, Fred Naggs & Sang Van Pham.

#### Diagnosis

*Cyclophorus courbeti* can be differentiated from all herein characterised *Cyclophorus* species based on DNA sequence data. Diagnostic nucleotide substitutions of the cytochrome *c* oxidase subunit I (COI) gene: 10:C, 25:C, 40:G, 127:A, 184:G, 214:A, 292:C, 294:C, 436:A, 493:T, 508:C, 514:G, 604:A, 638:A, 652:C, 653:C (numbers indicate the position in the studied COI fragment).

#### Remarks

Despite extensive effort, we were unable to locate type material of this species (see also [107]). Our determination was thus based on the description and figure in Ancey [92]. The description and the depicted shell matched sub-group I in size, shape (but the studied material of sub-group I had a less pronounced keel, which was, however, present in sub-group II) and colouration. The type locality given in Ancey [92] is “upper Tonkin, in the region of Lang Son and Bac Ninh” (original: “Haut-Tonkin, dans la région de Lang-son et de Bac-Ninh”), and our studied samples of sub-group I were collected from a locality in this area. We regard the material from eastern Thailand, which has been determined as *C*. *courbeti* by Nantarat et al. [47] (also included in the phylogenies of [6] and this study, herein THA011), as belonging to a different species. The studied material of *C*. *courbeti* has been found in northern Lang Son province (sub-group I) and in northeastern Bac Kan province (sub-group II) (Fig 4). Another herein characterised species, *C*. *subfloridus* (sub-group II), occurs sympatrically with *C*. *courbeti* (sub-group I) in northern Lang Son province. The shell shape of *C*. *subfloridus* is generally less compressed than that of *C*. *courbeti* (Fig 2). The shell of *C*. *subfloridus* sub-group II is smaller than that of *C*. *courbeti* sub-group I (Fig 3b) and the early whorls show a bluish colouration, while those of *C*. *courbeti* sub-group I lack a bluish colouration. The lip of *C*. *subfloridus* sub-group II tends to have a more whitish colour than that of *C*. *courbeti* sub-group I, which tends to be more yellowish or reddish.

***Cyclophorus fasciatus*** Kobelt, 1908 [48]

#### Conchological description

Medium-sized *Cyclophorus* species, overall shell shape rounded, aperture typically circular. Shell height 23.8 to 33.3 mm (mean = 29.0 mm), shell breadth 29.6 to 39.4 mm (mean = 34.2 mm) (sub-group I: shell height 26.2 to 33.3 mm (mean = 29.9 mm), shell breadth 32.5 to 39.4 mm (mean = 35.8 mm); sub-group II: shell height 23.8 to 32.2 mm (mean = 28.1 mm), shell breadth 29.6 to 36.0 mm (mean = 32.5 mm)). Number of whorls 4.75 to 5.25 (sub-group I: 4.75 to 5.25; sub-group II: 5 to 5.25). Early whorls usually without bluish colouration (sub-group I: usually without bluish colouration; sub-group II: without bluish colouration). Shape of body whorl usually uncompressed or slightly compressed; rounded, sub-angular or well-rounded (sub-group I: usually uncompressed or slightly compressed; rounded or sub-angular; sub-group II: usually uncompressed; rounded or well-rounded). Lip reflected and simple. Lip colour red, yellow, ivory, orange or white (sub-group I: yellow, ivory, orange or white; sub-group II: red). Wing-shaped projection at columellar margin usually absent (sub-group I: absent, or present and not pronounced; sub-group II: absent). Peripheral band usually dark brown (sub-group I: dark brown; sub-group II: usually dark brown). Colour pattern generally variable. Typical elements of pattern above position of peripheral band: zig-zag stripes, spiral bands, spiral lines and flames in different shades of brown or in white (sub-group I: zig-zag stripes, spiral bands, spiral lines and flames in different shades of brown or in white; sub-group II: zig-zag stripes, spiral bands, spiral lines and flames in different shades of brown). Typical elements of pattern below position of peripheral band: spiral lines, spiral bands and flames in different shades of brown (sub-group I: spiral lines, spiral bands and flames in darker shades of brown; sub-group II: spiral lines, spiral bands and flames in different shades of brown).

#### Displayed specimens

**Fig 6:**
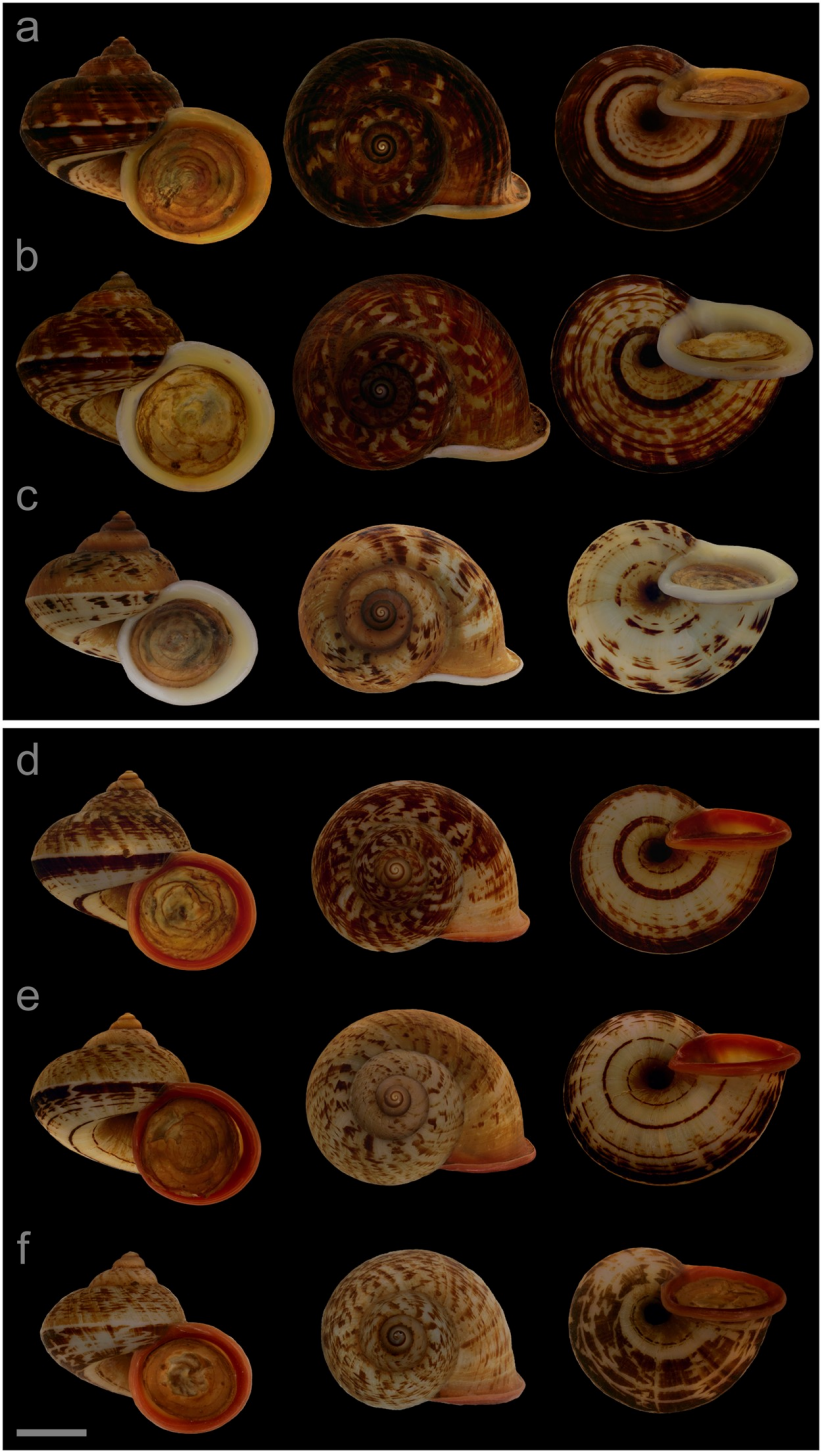
Specimens of *Cyclophorus fasciatus* Kobelt, 1908 [48]. Front, top and bottom view of (a) VNM005 (sub-group I), (b) VNM007 (sub-group I), (c) VNM008 (sub-group I), (d) VNM015 (sub-group II), (e) VNM013 (sub-group II) and (f) VNM014 (sub-group II). Scale bar: 10 mm.

**VNM005** (Fig 6a; sub-group I), specimen in ethanol; NHMUK 20110513/3; GenBank accession numbers MG720873 (COI), MG720923 (16S), MG720973 (28S); female individual; shell height 30.0 mm, shell breadth 35.5 mm, number of whorls 5.25. **VNM007** (Fig 6b; sub-group I), specimen in ethanol; NHMUK 20110513/2 (ethanol-preserved frozen tissue samples NHMUK Barcode 014041330, NHMUK Barcode 014041347); GenBank accession numbers MN153301 (COI), MN153350 (16S), MN153399 (28S); female individual; shell height 31.3 mm, shell breadth 36.1 mm, number of whorls 5.25. **VNM008** (Fig 6c; sub-group I), specimen in ethanol; NHMUK 20110513/4; GenBank accession numbers MG720874 (COI), MG720924 (16S), MG720974 (28S); male individual; shell height 28.0 mm, shell breadth 32.5 mm, number of whorls 5.25. **VNM015** (Fig 6d; sub-group II), specimen in ethanol; NHMUK 20110476/9; GenBank accession numbers MG720865 (COI), MG720915 (16S), MG720965 (28S); male individual; shell height 28.4 mm, shell breadth 31.6 mm, number of whorls 5.25. **VNM013** (Fig 6e; sub-group II), specimen in ethanol; NHMUK 20110476/7; GenBank accession numbers MG720863 (COI), MG720913 (16S), MG720963 (28S); female individual; shell height 27.9 mm, shell breadth 33.2 mm, number of whorls 5. **VNM014** (Fig 6f; sub-group II), specimen in ethanol; NHMUK 20110476/8; GenBank accession numbers MG720864 (COI), MG720914 (16S), MG720964 (28S); male individual; shell height 26.3 mm, shell breadth 30.0 mm, number of whorls 5.

#### Locality and sampling data

**VNM005**, **VNM007**, **VNM008**: Vietnam, Bac Kan, Ba Be National Park (22.40923°, 105.62855°); 18 May 2011; leg. Jonathan Ablett, Hao Van Luong, Fred Naggs & Sang Van Pham. **VNM015**, **VNM013**, **VNM014**: Vietnam, Thai Nguyen, Dong Dat commune (21.75523°, 105.70745°); 16 May 2011; leg. Jonathan Ablett, Hao Van Luong, Fred Naggs & Sang Van Pham.

#### Diagnosis

*Cyclophorus fasciatus* can be differentiated from all herein characterised *Cyclophorus* species based on DNA sequence data. Diagnostic nucleotide substitutions of the cytochrome *c* oxidase subunit I (COI) gene: 127:G, 184:A, 214:G/C, 232:C, 271:C/T, 313:A, 436:G, 493:C, 571:C, 604:G (numbers indicate the position in the studied COI fragment).

#### Remarks

This species was originally described as *Cyclophorus dodrans fasciatus*. The holotype (SMF 34732) resembled sub-group I in size (but the holotype was slightly larger), shape and colouration (but not in the exact colour pattern). The type locality given in Kobelt [48], “Residence in Annam” (original: “Aufenthalt in Annam”), is most likely incorrect as Zilch [108] states that “inside the shell, a locality label was found: Tuyen Quang, Tonkin. Fruhstorfer” (original: “im Inneren des Gehäuses fand sich ein Fundortzettel: Tuyen-Quang, Tonkin. Fruhstorfer”). Our studied samples of sub-group I were partly collected very close to northeastern Tuyen Quang. Further material from Tuyen Quang should be studied in future to confirm our determination. This species corresponds to lineage 03 in Oheimb et al. [6]. The studied material of *C*. *fasciatus* has been found in Ba Be National Park in northwestern Bac Kan province (sub-group I), in eastern Thai Nguyen province (sub-group I) and in central Thai Nguyen province (sub-group II) (Fig 4). None of the other herein characterised species is known to occur sympatrically with this species. *Cyclophorus subfloridus* (sub-group I) occurs in southwestern Lang Son province, approx. 20 km from where *C*. *fasciatus* (sub-group I) was found, in eastern Thai Nguyen province. The shell shape of *C*. *subfloridus* is generally slightly less compressed than that of *C*. *fasciatus* (Fig 2). The shell of *C*. *subfloridus* sub-group I is slightly smaller than that of *C*. *fasciatus* sub-group I (Fig 3b) and the early whorls show a bluish colouration, while those of *C*. *fasciatus* sub-group I usually lack a bluish colouration. The lip of *C*. *subfloridus* sub-group I tends to have a more whitish colour than that of *C*. *fasciatus* sub-group I, which tends to be more yellowish or reddish.

***Cyclophorus jourdyi*** Morlet, 1886 [93]

#### Conchological description

Medium-sized *Cyclophorus* species, overall shell shape rounded, aperture typically circular. Shell height 21.8 to 31.1 mm (mean = 27.6 mm), shell breadth 25.1 to 34.0 mm (mean = 30.6 mm) (sub-group I: shell height 26.0 to 31.1 mm (mean = 28.4 mm), shell breadth 28.0 to 34.0 mm (mean = 31.3 mm); sub-group II: shell height 21.8 to 24.8 mm (mean = 23.0 mm), shell breadth 25.1 to 29.1 mm (mean = 26.8 mm)). Number of whorls 5 to 5.5 (sub-group I: 5.25 to 5.5; sub-group II: 5). Early whorls with or without bluish colouration (sub-group I: with or without bluish colouration; sub-group II: with bluish colouration). Shape of body whorl usually uncompressed or slightly compressed; well-rounded (sub-group I: uncompressed or slightly compressed; well-rounded; sub-group II: uncompressed, slightly compressed or compressed; well-rounded). Lip usually reflected and simple (sub-group I: usually reflected and simple; sub-group II: reflected and simple). Lip colour white or ivory (sub-group I: ivory or white; sub-group II: white). Wing-shaped projection at columellar margin present and pronounced. Peripheral band dark brown. Colour pattern generally variable. Typical elements of pattern above position of peripheral band: flames, zig-zag stripes and spots in different shades of brown or in white (sub-group I: flames, zig-zag stripes and spots in different shades of brown or in white; sub-group II: flames and zig-zag stripes in darker shades of brown). Typical elements of pattern below position of peripheral band: spiral lines, spiral bands and flames in darker shades of brown (sub-group I: spiral lines, spiral bands and flames in darker shades of brown; sub-group II: spiral lines and flames in darker shades of brown).

#### Displayed specimens

**Fig 7:**
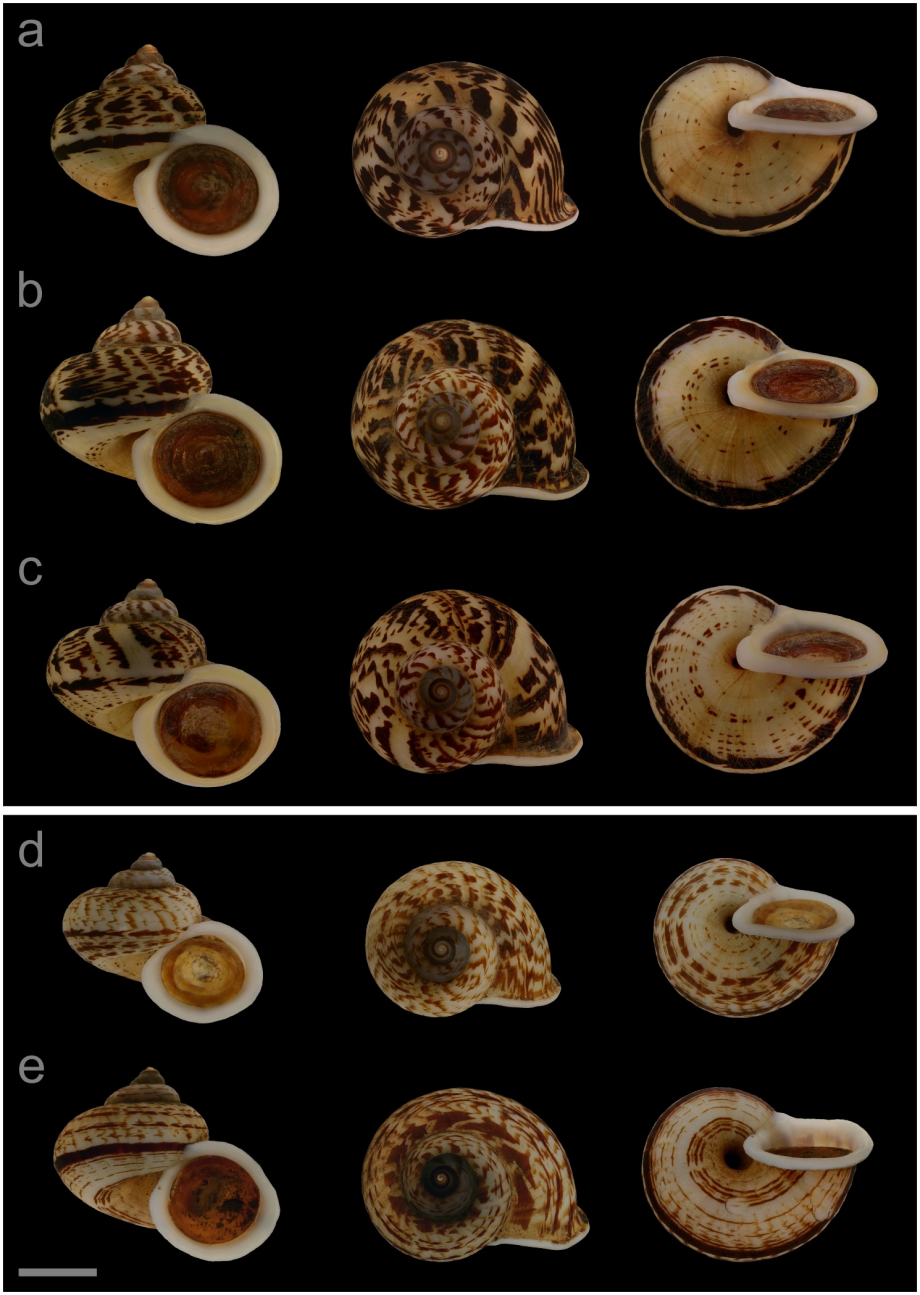
Specimens of *Cyclophorus jourdyi* Morlet, 1886 [93]. Front, top and bottom view of (a) VNM042 (sub-group I), (b) VNM043 (sub-group I), (c) VNM044 (sub-group I), (d) VNM045 (sub-group II) and (e) VNM046 (sub-group II). Scale bar: 10 mm.

**VNM042** (Fig 7a; sub-group I), specimen in ethanol; NHMUK 20110608/3; GenBank accession numbers MG720878 (COI), MG720928 (16S), MG720978 (28S); male individual; shell height 27.0 mm, shell breadth 29.8 mm, number of whorls 5.25. **VNM043** (Fig 7b; sub-group I), specimen in ethanol; NHMUK 20110608/4; GenBank accession numbers MN153319 (COI), MN153368 (16S), MN153415 (28S); female individual; shell height 28.1 mm, shell breadth 29.7 mm, number of whorls 5.5. **VNM044** (Fig 7c; sub-group I), specimen in ethanol; NHMUK 20110608/2 (ethanol-preserved frozen tissue sample NHMUK Barcode 014041336); GenBank accession numbers MG720877 (COI), MG720927 (16S), MG720977 (28S); female individual; shell height 26.5 mm, shell breadth 29.6 mm, number of whorls 5.25. **VNM045** (Fig 7d; sub-group II), specimen in ethanol; NHMUK 20110603/2; GenBank accession numbers MG720876 (COI), MG720926 (16S), MG720976 (28S); male individual; shell height 21.8 mm, shell breadth 25.1 mm, number of whorls 5. **VNM046** (Fig 7e; sub-group II), specimen in ethanol; NHMUK 20110603/1 (ethanol-preserved frozen tissue sample NHMUK Barcode 014041335); GenBank accession numbers MN153320 (COI), MN153369 (16S), MN153416 (28S); sex of individual not determined (parts of soft body damaged); shell height 24.8 mm, shell breadth 29.1 mm, number of whorls 5.

#### Locality and sampling data

**VNM042**, **VNM043**, **VNM044**: Vietnam, Quang Ninh, Ha Long, Ha Phong ward (20.96638°, 107.16610°); 25 May 2011; leg. Hao Van Luong, Fred Naggs & Sang Van Pham. **VNM045**, **VNM046**: Vietnam, Quang Ninh, Son Duong commune (21.06693°, 106.98883°); 24 May 2011; leg. Hao Van Luong.

#### Diagnosis

*Cyclophorus jourdyi* can be differentiated from all herein characterised *Cyclophorus* species based on DNA sequence data. Diagnostic nucleotide substitutions of the cytochrome *c* oxidase subunit I (COI) gene: 184:C, 325:C, 415:T, 458:G (numbers indicate the position in the studied COI fragment).

#### Remarks

The studied syntypes (MNHN-IM-2000-33830, MNHN-IM-2000-33831 and RBINS I.G.10591/MT.902, the latter labelled as paratype) resembled sub-group I and II in size, shape and colouration. No specific type locality is given in Morlet [93] besides “Tonkin”, where all taxa from the respective publication have been described from. In Morlet [109], however, the following locality is given for this species: “Tonkin: Ha Long Bay and Elephant Mountain” (original: “Tonkin: baie d’Halong et montagne de l’Éléphant”), which includes “Ha Long Bay / Tonkin” (original: “Baie d’Halong / Tonkin”) given on a label of the Brussels syntype (the Paris syntypes’ labels only state “Tonkin” (original: “Tonkin” and “Tonquin”)) and the location where our studied samples of sub-group I were collected. This species corresponds to lineage 07 in Oheimb et al. [6]. We regard the material from central Vietnam, which has been determined as *C*. *jourdyi* by Nantarat et al. [47] (also included in the phylogenies of [6] and this study, herein VNM029), as belonging to a different species. The studied material of *C*. *jourdyi* has been found in southern Quang Ninh province (sub-groups I and II) (Fig 4). None of the other herein characterised species is known to occur sympatrically with this species.

***Cyclophorus subfloridus*** Ancey, 1888 [92]

#### Conchological description

Medium-sized *Cyclophorus* species, overall shell shape rounded, aperture typically circular. Shell height 22.3 to 30.4 mm (mean = 26.7 mm), shell breadth 23.9 to 33.7 mm (mean = 29.6 mm) (sub-group I: shell height 25.2 to 30.4 mm (mean = 28.2 mm), shell breadth 29.0 to 33.7 mm (mean = 31.4 mm); sub-group II: shell height 22.3 to 27.4 mm (mean = 25.2 mm), shell breadth 23.9 to 30.3 mm (mean = 27.7 mm)). Number of whorls 5 to 5.5 (sub-group I: 5.25 to 5.5; sub-group II: 5 to 5.25). Early whorls with bluish colouration. Shape of body whorl usually uncompressed; rounded or well-rounded (sub-group I: usually uncompressed; well-rounded or rounded; sub-group II: uncompressed; usually rounded). Lip reflected and simple, or unreflected and duplicated (sub-group I: reflected and simple; sub-group II: reflected and simple, or unreflected and duplicated). Lip colour ivory or white. Wing-shaped projection at columellar margin absent, present and not pronounced, or present and pronounced (sub-group I: present and not pronounced, present and pronounced, or absent; sub-group II: absent, or present and not pronounced). Peripheral band usually dark brown (sub-group I: dark brown; sub-group II: usually dark brown). Colour pattern generally variable. Typical elements of pattern above position of peripheral band: zig-zag stripes and flames in different shades of brown or in white (sub-group I: zig-zag stripes and flames in different shades of brown; sub-group II: zig-zag stripes and flames in different shades of brown or in white). Typical elements of pattern below position of peripheral band: spiral lines, spiral bands, zig-zag stripes and transverse lines in different shades of brown or in white (sub-group I: spiral lines, spiral bands and transverse lines in darker shades of brown; sub-group II: zig-zag stripes, spiral bands and spiral lines in different shades of brown or in white).

#### Displayed specimens

**Fig 8:**
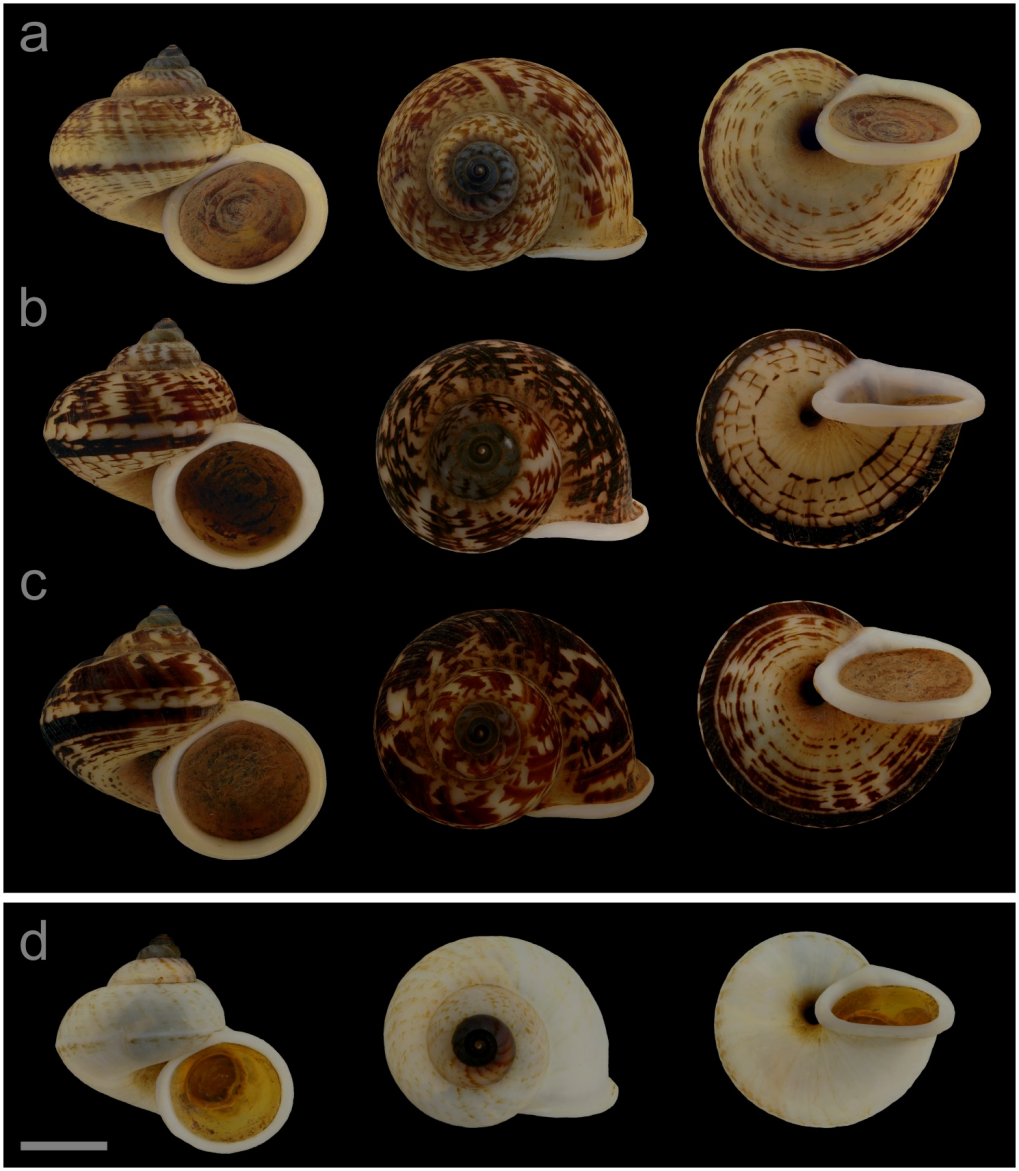
Specimens of *Cyclophorus subfloridus* Ancey, 1888 [92]. Front, top and bottom view of (a) VNM063 (sub-group I), (b) VNM059 (sub-group I), (c) VNM061 (sub-group I) and (d) VNM065 (sub-group II). Scale bar: 10 mm.

**VNM063** (Fig 8a; sub-group I), specimen in ethanol; NHMUK 20140597 (ethanol-preserved frozen tissue sample NHMUK Barcode 014041340); GenBank accession numbers MN153334 (COI), MN153383 (16S), MN153430 (28S); female individual; shell height 27.6 mm, shell breadth 31.0 mm, number of whorls 5.25. **VNM059** (Fig 8b; sub-group I), specimen in ethanol; NHMUK 20140557/2; GenBank accession numbers MN153330 (COI), MN153379 (16S), MN153426 (28S); male individual; shell height 28.2 mm, shell breadth 31.1 mm, number of whorls 5.25. **VNM061** (Fig 8c; sub-group I), specimen in ethanol; NHMUK 20140585/1 (ethanol-preserved frozen tissue sample NHMUK Barcode 014041339); GenBank accession numbers MN153332 (COI), MN153381 (16S), MN153428 (28S); female individual; shell height 28.4 mm, shell breadth 31.5 mm, number of whorls 5.25. **VNM065** (Fig 8d; sub-group II), specimen in ethanol; NHMUK 20110594 (ethanol-preserved frozen tissue samples NHMUK Barcode 014041334, NHMUK Barcode 014041351); GenBank accession numbers MN153336 (COI), MN153385 (16S), MN153432 (28S); sex of individual not determined (parts of soft body damaged); shell height 24.7 mm, shell breadth 26.8 mm, number of whorls 5.25.

#### Locality and sampling data

**VNM063**: Vietnam, Lang Son, Yen Thinh commune (21.61862°, 106.34760°); 19 September 2013; leg. Jonathan Ablett, Hao Van Luong, Fred Naggs & Sang Van Pham. **VNM059**: Vietnam, Lang Son, Huu Lien commune, Huu Lien Nature Reserve (21.66235°–21.65998°, 106.36633°–106.36462°); 17 September 2013; leg. Jonathan Ablett, Hao Van Luong, Fred Naggs & Sang Van Pham. **VNM061**: Vietnam, Lang Son, Yen Thinh commune (21.65948°, 106.36535°); 18 September 2013; leg. Jonathan Ablett, Hao Van Luong, Fred Naggs & Sang Van Pham. **VNM065**: Vietnam, Lang Son, Trung Quan commune (22.13137°, 106.58715°); 23 May 2011; leg. Hao Van Luong, Fred Naggs & Sang Van Pham.

#### Diagnosis

*Cyclophorus subfloridus* can be differentiated from all herein characterised *Cyclophorus* species based on DNA sequence data. Diagnostic nucleotide substitutions of the cytochrome *c* oxidase subunit I (COI) gene: 217:A (number indicates the position in the studied COI fragment).

#### Remarks

This species was originally described as *Cyclophorus fulguratus* var. *subflorida*. Despite extensive effort, we were unable to locate type material of this species (see also [107]). Our determination was thus based on the description and figure in Ancey [92]. The description and the depicted shell matched sub-group I in size, shape and colouration. The material of *C*. *fulguratus* studied in Ancey [92], which includes the therein newly described *C*. *fulguratus* var. *subflorida* (a specific type locality for this taxon is not given), is “from Lang Son to Bac Ninh” (original: “De Lang-son a Bac-Ninh”), and our studied samples of sub-group I were collected from localities in this area. We regard *Cyclophorus fulguratus* var. *barniana* Ancey, 1888 [92] as a synonym of this species given that the type material of both taxa was presumably collected in the same region [92] and that the described morphological differences between them are insignificant (note that no type material of *C*. *fulguratus* var. *barniana* could be located; see also [107]). *Cyclophorus jourdyi* var. *longsonensis* Morlet, 1891 [110] is regarded as a further synonym of *C*. *subfloridus* given that its type locality is “Lang Son” (original: “Long-son”) and that the studied type material (RBINS I.G.10591/MT.903, labelled as paratype, and MNHN-IM-2000-34483 and MNHN-IM-2000-34484, both potential syntypes) resembled sub-group I of *C*. *subfloridus* in size, shape and colouration (only in MNHN-IM-2000-34483 the early whorls lacked a bluish colouration). We regard the material from northern Thailand, which has been determined as *C*. *subfloridus* by Nantarat et al. [47] (also included in the phylogenies of [6] and this study, herein THA025), as belonging to a different species. The studied material of *C*. *subfloridus* has been found in southwestern Lang Son province, partly in Huu Lien Nature Reserve, (sub-group I) and in northern Lang Son province (sub-group II) (Fig 4). Another herein characterised species, *C*. *courbeti* (sub-group I), occurs sympatrically with *C*. *subfloridus* (sub-group II) in northern Lang Son province. The shell shape of *C*. *courbeti* is generally more compressed than that of *C*. *subfloridus* (Fig 2). The shell of *C*. *courbeti* sub-group I is larger than that of *C*. *subfloridus* sub-group II (Fig 3b) and the early whorls lack a bluish colouration, while those of *C*. *subfloridus* sub-group II show a bluish colouration. The lip of *C*. *courbeti* sub-group I tends to have a more yellowish or reddish colour than that of *C*. *subfloridus* sub-group II, which tends to be more whitish. Furthermore, *C*. *fasciatus* (sub-group I) occurs in eastern Thai Nguyen province, approx. 20 km from where *C*. *subfloridus* (sub-group I) was found, in southwestern Lang Son province. The shell shape of *C*. *fasciatus* is generally slightly more compressed than that of *C*. *subfloridus* (Fig 2). The shell of *C*. *fasciatus* sub-group I is slightly larger than that of *C*. *subfloridus* sub-group I (Fig 3b) and the early whorls usually lack a bluish colouration, while those of *C*. *subfloridus* sub-group I show a bluish colouration. The lip of *C*. *fasciatus* sub-group I tends to have a more yellowish or reddish colour than that of *C*. *subfloridus* sub-group I, which tends to be more whitish.

***Cyclophorus cucphuongensis*** K. C. M. von Oheimb, sp. nov.

urn:lsid:zoobank.org:act:0E86BA4B-769A-4D8D-AD00-ACA4EAEC82D6

#### Etymology

The name refers to the type locality of this species in Cuc Phuong National Park.

#### Conchological description

Medium-sized *Cyclophorus* species, overall shell shape rounded, aperture typically circular. Shell height 23.3 to 29.9 mm (mean = 26.5 mm), shell breadth 28.7 to 35.4 mm (mean = 31.4 mm). Number of whorls 5 to 5.25. Early whorls without or with bluish colouration. Shape of body whorl usually uncompressed or slightly compressed; rounded or well-rounded. Lip reflected and simple. Lip colour yellow or ivory. Wing-shaped projection at columellar margin present and pronounced, or present and not pronounced. Peripheral band usually dark brown, medium brown, or not distinguishable from background colouration. Colour pattern generally variable. Typical elements of pattern above position of peripheral band: flames and zig-zag stripes in different shades of brown. Typical elements of pattern below position of peripheral band: spiral lines and spiral bands in different shades of brown.

#### Type specimens

**Fig 9:**
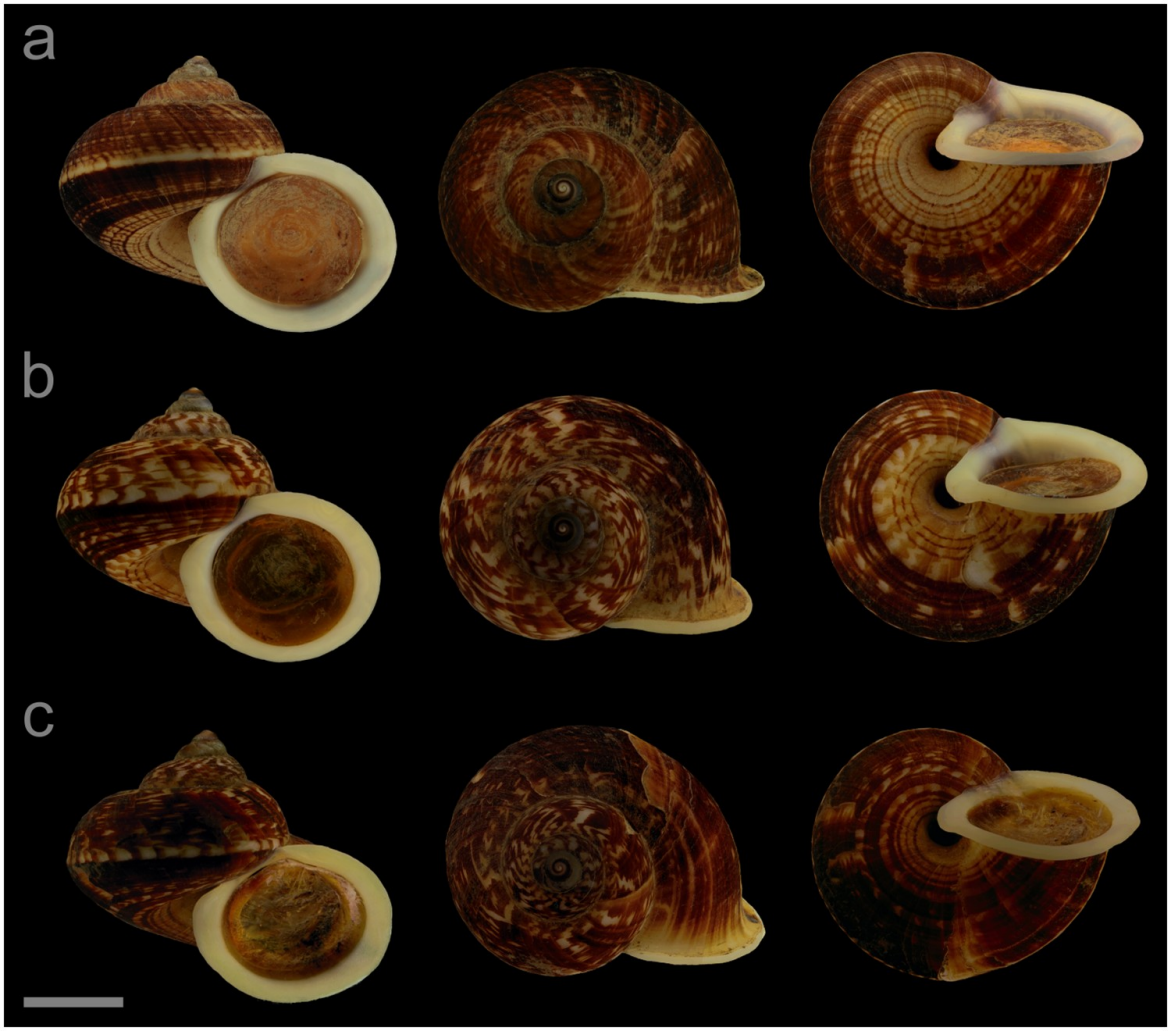
Specimens of *Cyclophorus cucphuongensis* Oheimb, sp. nov. Front, top and bottom view of (a) VNM052 (holotype), (b) VNM053 (paratype) and (c) VNM054 (paratype). Scale bar: 10 mm.

**VNM052** (Fig 9a; holotype), specimen in ethanol; NHMUK 20140276/4; GenBank accession numbers MG720887 (COI), MG720937 (16S), MG720987 (28S); female individual; shell height 27.2 mm, shell breadth 32.7 mm, number of whorls 5.25. **VNM053** (Fig 9b; paratype), specimen in ethanol; NHMUK 20140287/2 (ethanol-preserved frozen tissue sample NHMUK Barcode 014041337); GenBank accession numbers MN153325 (COI), MN153374 (16S), MN153421 (28S); male individual; shell height 27.0 mm, shell breadth 31.3 mm, number of whorls 5.25. **VNM054** (Fig 9c; paratype), specimen in ethanol; NHMUK 20140287/3 (ethanol-preserved frozen tissue sample NHMUK Barcode 014041338; viable cell preparation NHMUK Barcode 014041352); GenBank accession numbers MN153326 (COI), MN153375 (16S), MN153422 (28S); male individual; shell height 27.0 mm, shell breadth 31.1 mm, number of whorls 5.25.

#### Locality and sampling data

**VNM052**: Vietnam, Ninh Binh, Cuc Phuong National Park (20.28845°, 105.66767°); 07 September 2013; leg. Hao Van Luong & team. **VNM053**, **VNM054**: Vietnam, Ninh Binh, Cuc Phuong National Park (20.35548°, 105.63342°); 08 September 2013; leg. Hao Van Luong & team.

#### Diagnosis

*Cyclophorus cucphuongensis* sp. nov. can be differentiated from all herein characterised *Cyclophorus* species based on DNA sequence data. Diagnostic nucleotide substitutions of the cytochrome *c* oxidase subunit I (COI) gene: 89:T, 91:C, 208:T, 289:T, 343:C, 401:C, 487:C, 556:G, 574:G/C (numbers indicate the position in the studied COI fragment).

#### Remarks

This species corresponds to lineage 10 in Oheimb et al. [6]. The studied material of *C*. *cucphuongensis* has been found in central and eastern Cuc Phuong National Park in both western Ninh Binh province and southeastern Hoa Binh province (Fig 4). None of the other herein characterised species is known to occur sympatrically with this species. *Cyclophorus takumisaitoi* occurs in northwestern Cuc Phuong National Park. The shell shape of *C*. *takumisaitoi* is similar to that of *C*. *cucphuongensis* (Fig 2). The shell of *C*. *takumisaitoi* is slightly smaller than that of *C*. *cucphuongensis* (Fig 3a). The lip of *C*. *takumisaitoi* tends to have a more reddish colour than that of *C*. *cucphuongensis*, which tends to be more yellowish. The wing-shaped projection at the columellar margin is absent in *C*. *takumisaitoi*, while being present in *C*. *cucphuongensis*, and the shell colouration of *C*. *takumisaitoi* is more uniform with a less prominent peripheral band. Furthermore, *C*. *paracucphuongensis* occurs in western Cuc Phuong National Park. The shell shape of *C*. *paracucphuongensis* is similar to that of *C*. *cucphuongensis* (Fig 2). The shell of *C*. *paracucphuongensis* is of similar size as that of *C*. *cucphuongensis* (Fig 3a). On the basis of currently available shell morphological data, these two species cannot be differentiated.

***Cyclophorus paracucphuongensis*** K. C. M. von Oheimb, sp. nov.

urn:lsid:zoobank.org:act:803C12C9-D401-49C7-835E-3922EF9873B4

#### Etymology

The name refers to *C*. *cucphuongensis*, which is morphologically similar to this species.

#### Conchological description

Medium-sized *Cyclophorus* species, overall shell shape rounded, aperture typically circular. Shell height 24.4 to 26.7 mm (mean = 25.8 mm), shell breadth 28.2 to 30.9 mm (mean = 29.8 mm). Number of whorls 5 to 5.25. Early whorls without bluish colouration. Shape of body whorl uncompressed; rounded or sub-angular. Lip reflected and simple. Lip colour yellow or ivory. Wing-shaped projection at columellar margin absent, present and not pronounced, or present and pronounced. Peripheral band dark brown. Colour pattern generally variable. Typical elements of pattern above position of peripheral band: zig-zag stripes and flames in different shades of brown. Typical elements of pattern below position of peripheral band: spiral bands and spiral lines in darker shades of brown.

#### Type specimens

**Fig 10:**
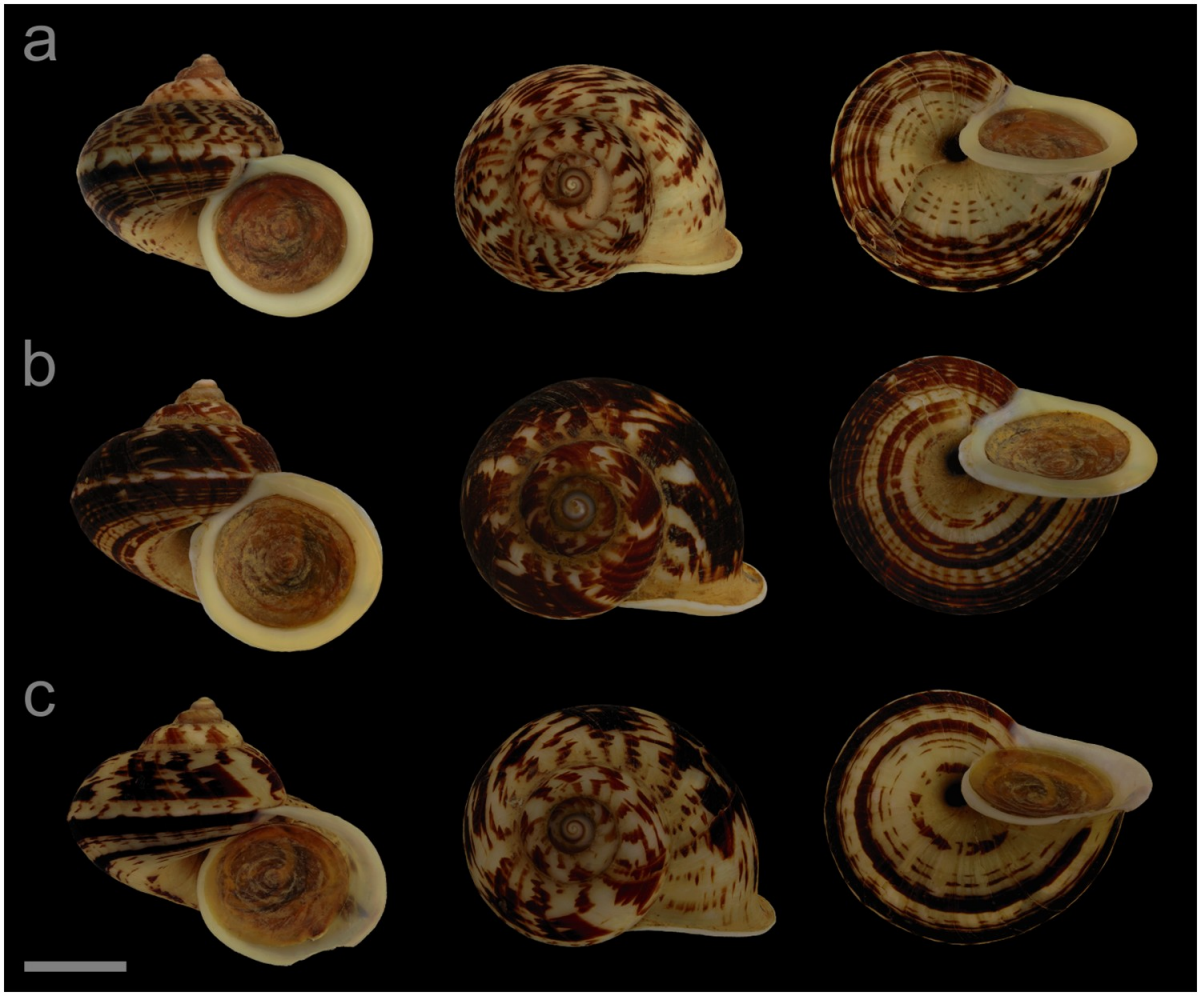
Specimens of *Cyclophorus paracucphuongensis* Oheimb, sp. nov. Front, top and bottom view of (a) VNM069 (holotype), (b) VNM071 (paratype) and (c) VNM070 (paratype). Scale bar: 10 mm.

**VNM069** (Fig 10a; holotype), specimen in ethanol; NHMUK 20180432; GenBank accession numbers MN153337 (COI), MN153386 (16S), MN153433 (28S); male individual; shell height 25.6 mm, shell breadth 28.6 mm, number of whorls 5.25. **VNM071** (Fig 10b; paratype), specimen in ethanol; NHMUK 20180433; GenBank accession numbers MN153339 (COI), MN153388 (16S), MN153435 (28S); male individual; shell height 26.4 mm, shell breadth 30.3 mm, number of whorls approx. 5 (pieces of first whorl missing). **VNM070** (Fig 10c; paratype), specimen in ethanol; NHMUK 20180434; GenBank accession numbers MN153338 (COI), MN153387 (16S), MN153434 (28S); female individual; shell height 26.1 mm, shell breadth 30.9 mm, number of whorls 5.25.

#### Locality and sampling data

**VNM069**, **VNM071**, **VNM070**: Vietnam, Thanh Hoa, Cuc Phuong National Park (20.35583°, 105.51088°); 02 May 2009; leg. Hao Van Luong.

#### Diagnosis

*Cyclophorus paracucphuongensis* sp. nov. can be differentiated from all herein characterised *Cyclophorus* species based on DNA sequence data. Diagnostic nucleotide substitutions of the cytochrome *c* oxidase subunit I (COI) gene: 38:A, 485:C (numbers indicate the position in the studied COI fragment).

#### Remarks

The studied material of *C*. *paracucphuongensis* has been found in western Cuc Phuong National Park in northern Thanh Hoa province (Fig 4). None of the other herein characterised species is known to occur sympatrically with this species. *Cyclophorus takumisaitoi* occurs in northwestern Cuc Phuong National Park. The shell shape of *C*. *takumisaitoi* is similar to that of *C*. *paracucphuongensis* (Fig 2). The shell of *C*. *takumisaitoi* is slightly smaller than that of *C*. *paracucphuongensis* (Fig 3a). The lip of *C*. *takumisaitoi* tends to have a more reddish colour than that of *C*. *paracucphuongensis*, which tends to be more yellowish. The shell colouration of *C*. *takumisaitoi* is more uniform with a less prominent peripheral band. Furthermore, *C*. *cucphuongensis* occurs in central and eastern Cuc Phuong National Park. The shell shape of *C*. *cucphuongensis* is similar to that of *C*. *paracucphuongensis* (Fig 2). The shell of *C*. *cucphuongensis* is of similar size as that of *C*. *paracucphuongensis* (Fig 3a). On the basis of currently available shell morphological data, these two species cannot be differentiated.

***Cyclophorus phongnhakebangensis*** P. V. von Oheimb, sp. nov.

urn:lsid:zoobank.org:act:FD4FE74D-974A-4C6F-B968-15A09D2B1BB0

#### Etymology

The name refers to the type locality of this species in Phong Nha-Ke Bang National Park.

#### Conchological description

Medium-sized *Cyclophorus* species, overall shell shape rounded, aperture typically circular. Shell height 20.5 to 26.2 mm (mean = 24.1 mm), shell breadth 26.0 to 32.8 mm (mean = 29.8 mm). Number of whorls 4.75 to 5.25. Early whorls without or with bluish colouration. Shape of body whorl slightly compressed or compressed; usually rounded. Lip reflected and simple. Lip colour ivory, white or peach. Wing-shaped projection at columellar margin absent, or present and not pronounced. Peripheral band usually dark brown. Colour pattern generally variable. Typical elements of pattern above position of peripheral band: zig-zag stripes, spiral lines and spiral bands in different shades of brown. Typical elements of pattern below position of peripheral band: spiral lines, spiral bands and transverse lines in darker shades of brown.

#### Type specimens

**Fig 11:**
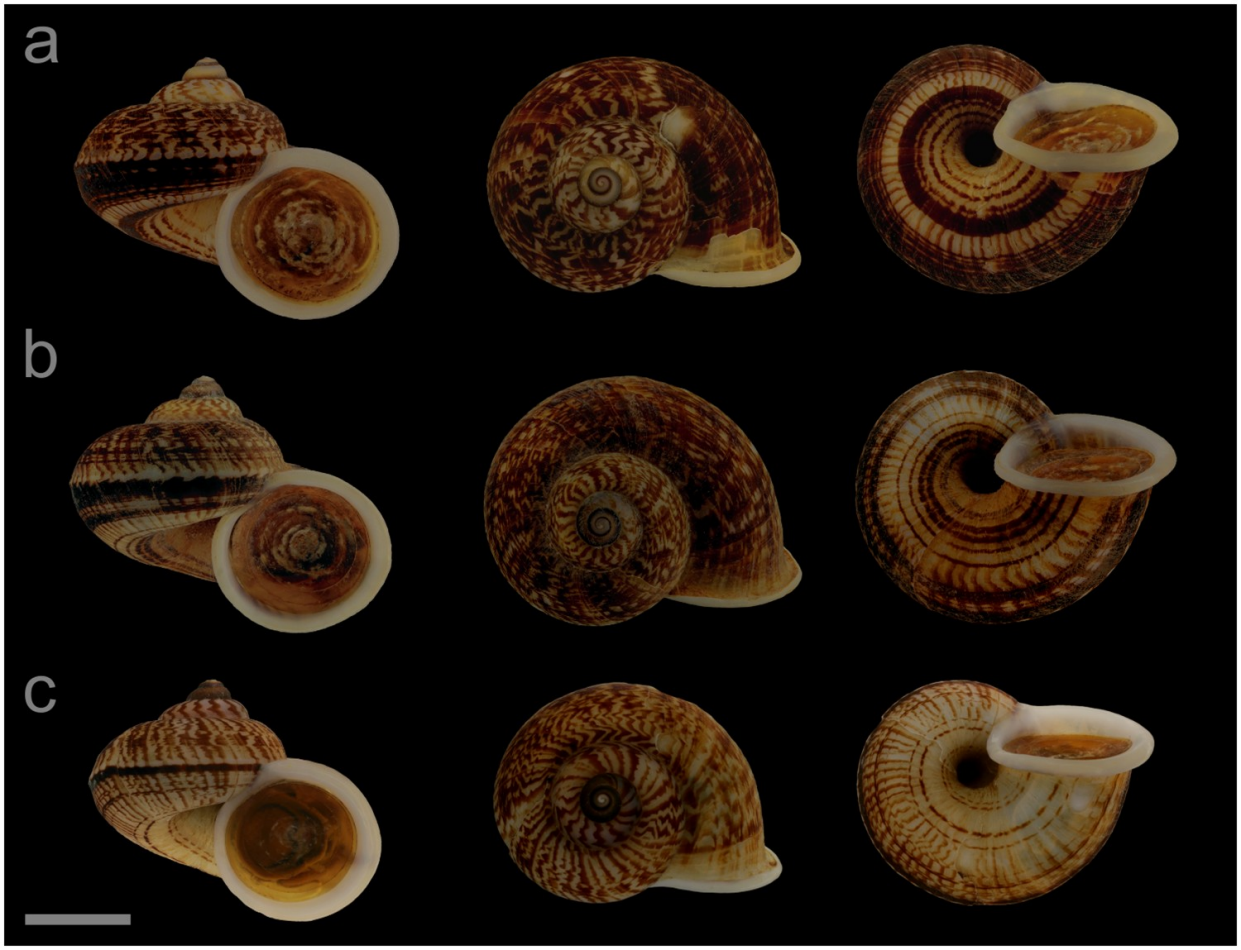
Specimens of *Cyclophorus phongnhakebangensis* Oheimb, sp. nov. Front, top and bottom view of (a) VNM032 (holotype), (b) VNM035 (paratype) and (c) VNM031 (paratype). Scale bar: 10 mm.

**VNM032** (Fig 11a; holotype), specimen in ethanol; NHMUK 20130904/4; GenBank accession numbers MN153314 (COI), MN153363 (16S), MN153410 (28S); female individual; shell height 24.5 mm, shell breadth 29.6 mm, number of whorls 5. **VNM035** (Fig 11b; paratype), specimen in ethanol; NHMUK 20130894/3; GenBank accession numbers MN153315 (COI), MN153364 (16S), MN153411 (28S); female individual; shell height 24.0 mm, shell breadth 29.4 mm, number of whorls 5. **VNM031** (Fig 11c; paratype), specimen in ethanol; NHMUK 20130904/3; GenBank accession numbers MG720883 (COI), MG720933 (16S), MG720983 (28S); male individual; shell height 23.2 mm, shell breadth 27.3 mm, number of whorls 5.

#### Locality and sampling data

**VNM032**, **VNM031**: Vietnam, Quang Binh, Phong Nha-Ke Bang National Park (17.50317°, 106.26045°); 03 March 2012; leg. Jonathan Ablett, Hao Van Luong, Fred Naggs & Sang Van Pham. **VNM035**: Vietnam, Quang Binh, Phong Nha-Ke Bang National Park (17.54147°, 106.23532°); 03 March 2012; leg. Jonathan Ablett, Hao Van Luong, Fred Naggs & Sang Van Pham.

#### Diagnosis

*Cyclophorus phongnhakebangensis* sp. nov. can be differentiated from all herein characterised *Cyclophorus* species based on DNA sequence data. Diagnostic nucleotide substitutions of the cytochrome *c* oxidase subunit I (COI) gene: 46:G, 95:C, 133:A, 178:C, 242:C, 259:C, 277:C, 388:G, 391:A/G, 409:G, 412:C, 415:C, 433:T, 439:A, 473:A, 474:G, 475:C, 544:G, 565:C, 595:C, 637:C (numbers indicate the position in the studied COI fragment).

#### Remarks

This species corresponds to lineage 06 in Oheimb et al. [6]. The studied material of *C*. *phongnhakebangensis* has been found in Phong Nha-Ke Bang National Park in central Quang Binh province (Fig 4). None of the other herein characterised species is known to occur sympatrically with this species.

***Cyclophorus takumisaitoi*** T. Hirano, sp. nov.

urn:lsid:zoobank.org:act:6D497FF9-5849-49BB-8162-665FADA40C34

#### Etymology

The name refers to the malacologist and evolutionary biologist Takumi Saito, who collected individuals of this species together with Tu Van Do and Takahiro Hirano.

#### Conchological description

Medium-sized *Cyclophorus* species, overall shell shape rounded, aperture typically circular. Shell height 22.7 to 25.0 mm (mean = 23.8 mm), shell breadth 27.1 to 29.2 mm (mean = 28.0 mm). Number of whorls 5 to 5.25. Early whorls without bluish colouration. Shape of body whorl uncompressed or slightly compressed; rounded or well-rounded. Lip reflected and simple. Lip colour orange, peach or yellow. Wing-shaped projection at columellar margin absent. Peripheral band not distinguishable from background colouration, or absent. Colour pattern generally variable. Typical elements of pattern above position of peripheral band: zig-zag stripes and flames in lighter shades of brown. Typical elements of pattern below position of peripheral band: spiral bands in darker shades of brown.

#### Type specimens

**Fig 12:**
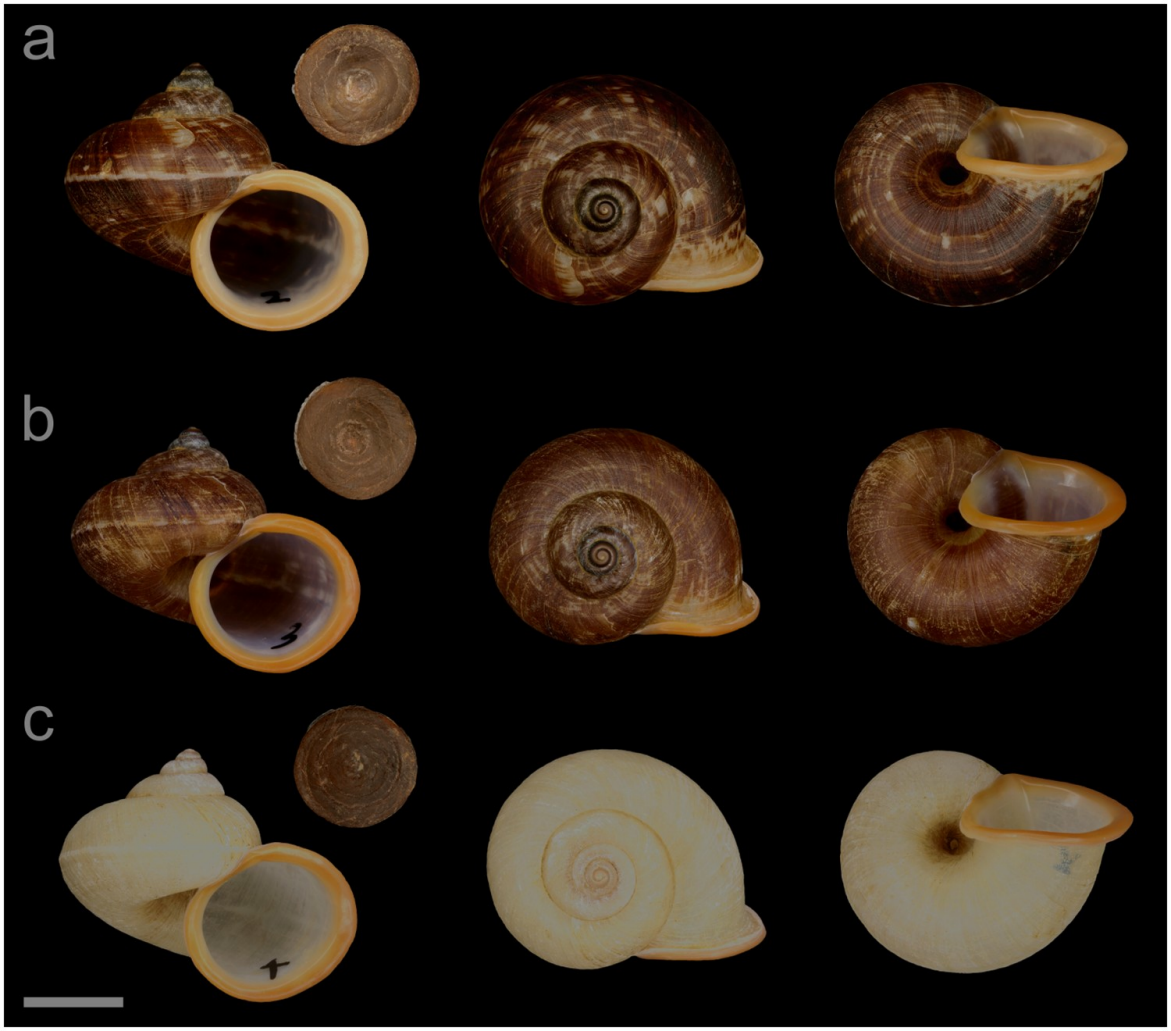
Specimens of *Cyclophorus takumisaitoi* Hirano, sp. nov. Front, top and bottom view of (a) VNM076 (holotype), (b) VNM078 (paratype) and (c) VNM077 (paratype). Scale bar: 10 mm.

**VNM076** (Fig 12a; holotype), soft body in ethanol (previously in formalin) and dry shell; NHMUK 20180455 (ethanol-preserved frozen tissue sample NHMUK Barcode 014041344); GenBank accession numbers MN153344 (COI), MN153393 (16S), MN153440 (28S); male individual; shell height 25.0 mm, shell breadth 28.2 mm, number of whorls 5.25. **VNM078** (Fig 12b; paratype), soft body in ethanol (previously in formalin) and dry shell; NHMUK 20180456 (ethanol-preserved frozen tissue sample NHMUK Barcode 014041345); GenBank accession numbers MN153346 (COI), MN153395 (16S), MN153442 (28S); male individual; shell height 23.2 mm, shell breadth 27.1 mm, number of whorls 5. **VNM077** (Fig 12c; paratype), soft body in ethanol (previously in formalin) and dry shell; NHMUK 20180457 (ethanol-preserved frozen tissue sample NHMUK Barcode 014041346); GenBank accession numbers MN153345 (COI), MN153394 (16S), MN153441 (28S); male individual; shell height 23.4 mm, shell breadth 28.0 mm, number of whorls 5.25.

#### Locality and sampling data

**VNM076**, **VNM078**, **VNM077**: Vietnam, Hoa Binh, Cuc Phuong National Park (20.38493°, 105.52280°); 26 May 2016; leg. Tu Van Do, Takahiro Hirano & Takumi Saito.

#### Diagnosis

*Cyclophorus takumisaitoi* sp. nov. can be differentiated from all herein characterised *Cyclophorus* species based on DNA sequence data. Diagnostic nucleotide substitutions of the cytochrome *c* oxidase subunit I (COI) gene: 241:A, 319:C, 349:T, 430:G, 499:A (numbers indicate the position in the studied COI fragment).

#### Remarks

The studied material of *C*. *takumisaitoi* has been found in northwestern Cuc Phuong National Park in southeastern Hoa Binh province (Fig 4). None of the other herein characterised species is known to occur sympatrically with this species. *Cyclophorus cucphuongensis* occurs in central and eastern Cuc Phuong National Park. The shell shape of *C*. *cucphuongensis* is similar to that of *C*. *takumisaitoi* (Fig 2). The shell of *C*. *cucphuongensis* is slightly larger than that of *C*. *takumisaitoi* (Fig 3a). The lip of *C*. *cucphuongensis* tends to have a more yellowish colour than that of *C*. *takumisaitoi*, which tends to be more reddish. The wing-shaped projection at the columellar margin is present in *C*. *cucphuongensis*, while being absent in *C*. *takumisaitoi*, and the shell colouration of *C*. *cucphuongensis* is more vivid with a more prominent peripheral band. Furthermore, *C*. *paracucphuongensis* occurs in western Cuc Phuong National Park. The shell shape of *C*. *paracucphuongensis* is similar to that of *C*. *takumisaitoi* (Fig 2). The shell of *C*. *paracucphuongensis* is slightly larger than that of *C*. *takumisaitoi* (Fig 3a). The lip of *C*. *paracucphuongensis* tends to have a more yellowish colour than that of *C*. *takumisaitoi*, which tends to be more reddish. The shell colouration of *C*. *paracucphuongensis* is more vivid with a more prominent peripheral band.

***Cyclophorus* sp**. **1**

#### Conchological description

Medium-sized *Cyclophorus* species, overall shell shape rounded, aperture typically circular. Shell height 23.1 to 26.5 mm (mean = 25.0 mm), shell breadth 28.7 to 31.9 mm (mean = 29.9 mm). Number of whorls 5.25. Early whorls with or without bluish colouration. Shape of body whorl uncompressed or slightly compressed; rounded or well-rounded. Lip reflected and simple. Lip colour yellow. Wing-shaped projection at columellar margin present and not pronounced, absent, or present and pronounced. Peripheral band dark brown or absent. Colour pattern generally variable. Typical elements of pattern above position of peripheral band: spiral lines, spiral bands and zig-zag stripes in different shades of brown. Typical elements of pattern below position of peripheral band: spiral lines, spiral bands and transverse lines in darker shades of brown.

#### Displayed specimens

**Fig 13:**
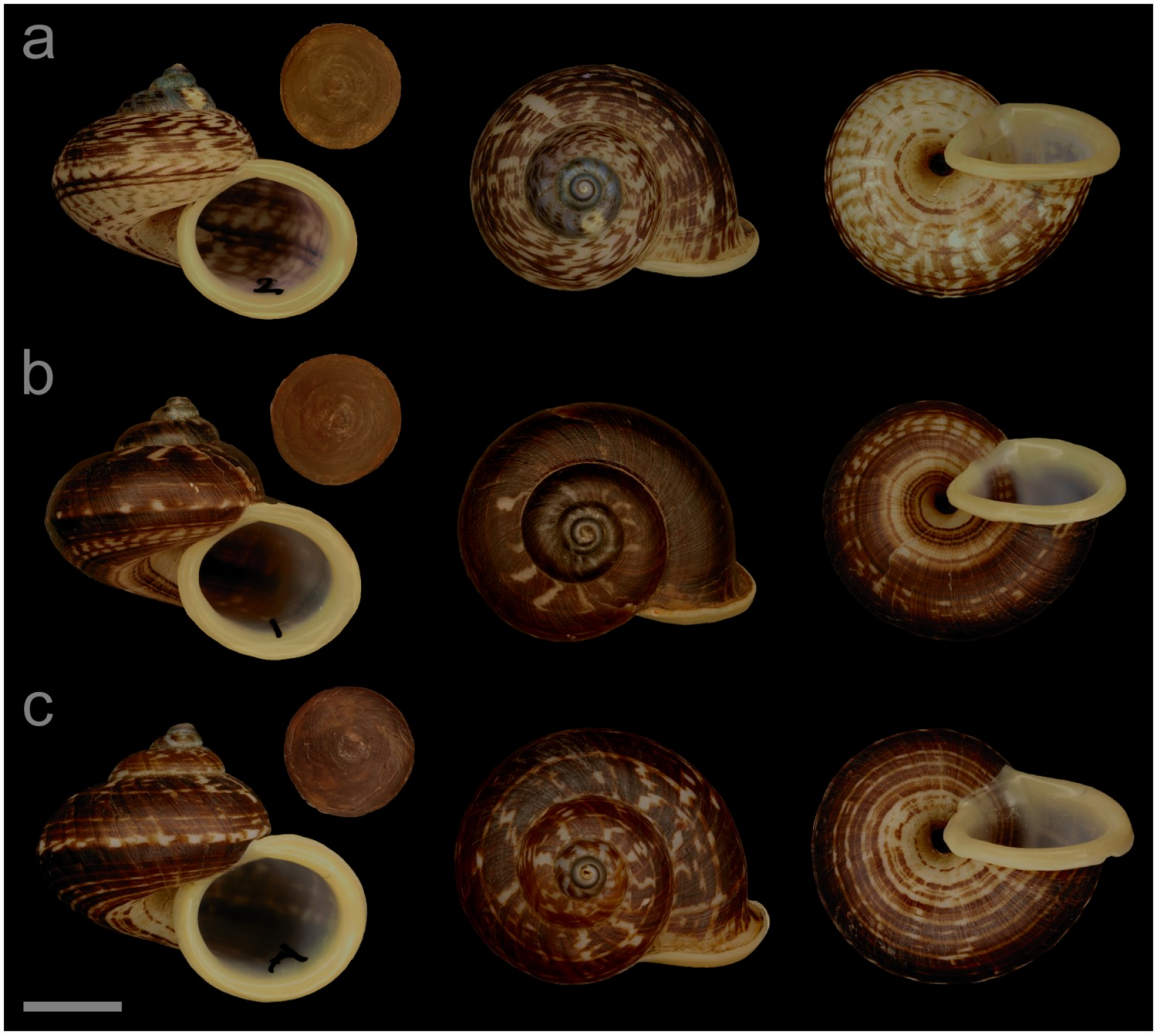
Specimens of *Cyclophorus* sp. 1. Front, top and bottom view of (a) VNM073, (b) VNM074 and (c) VNM075. Scale bar: 10 mm.

**VNM073** (Fig 13a), soft body in ethanol (previously in formalin) and dry shell; NHMUK 20180448 (ethanol-preserved frozen tissue sample NHMUK Barcode 014041342); GenBank accession numbers MN153341 (COI), MN153390 (16S), MN153437 (28S); male individual; shell height 24.6 mm, shell breadth 28.7 mm, number of whorls 5.25. **VNM074** (Fig 13b), soft body in ethanol (previously in formalin) and dry shell; NHMUK 20180447 (ethanol-preserved frozen tissue sample NHMUK Barcode 014041341); GenBank accession numbers MN153342 (COI), MN153391 (16S), MN153438 (28S); male individual; shell height 25.5 mm, shell breadth 29.5 mm, number of whorls approx. 5.25 (pieces of first whorl missing). **VNM075** (Fig 13c), soft body in ethanol (previously in formalin) and dry shell; NHMUK 20180452 (ethanol-preserved frozen tissue sample NHMUK Barcode 014041343); GenBank accession numbers MN153343 (COI), MN153392 (16S), MN153439 (28S); female individual; shell height 26.5 mm, shell breadth 30.7 mm, number of whorls approx. 5.25 (pieces of first whorl missing).

#### Locality and sampling data

**VNM073**, **VNM074**, **VNM075**: Vietnam, Hoa Binh, Cao Duong commune, bought at market stall (20.72542°, 105.64899°), collected nearby according to the merchant; purchased on 26 May 2016; purchased by Tu Van Do, Takahiro Hirano & Takumi Saito.

#### Diagnosis

*Cyclophorus* sp. 1 can be differentiated from all herein characterised *Cyclophorus* species based on DNA sequence data. Diagnostic nucleotide substitutions of the cytochrome *c* oxidase subunit I (COI) gene: 55:A, 94:A, 97:G, 412:G, 511:G (numbers indicate the position in the studied COI fragment).

#### Remarks

This species is so far undescribed. As the studied material of *Cyclophorus* sp. 1 has been bought on a market, the species’ exact distribution remains unknown.

## Supporting information

S1 Table List of studied *Cyclophorus* specimens. Including specimen code and details, locality and sampling information, taxon, mutual group, shell morphology classification and GenBank accession numbers. All material is held at the Natural History Museum, London. Primers used for new COI sequences: ^1^LCO1490/HCO2198, ^2^PF372/HCO2198, ^3^Cyc-F01/HCO2198; primers used for new 28S sequences: ^1^28SF4/28SR5, ^2^28SF4/LSU-4. See S2 Table for VNM001 and VNM029.
(PDF)

S2 Table List of additional samples taken from the literature. Including specimen code, taxon and GenBank accession numbers.
(PDF)

S1 Fig Additional consensus Bayesian phylogeny of *Cyclophorus* spp. The phylogeny is based on sequence data from the COI and 16S genes and was used for the bPTP analysis. Bayesian posterior probabilities are provided at the respective nodes. The scale bar indicates the number of substitutions per site according to the applied model of sequence evolution. Specimen codes (S1 and S2 Tables) refer to outgroups (“OUT”) and respective *Cyclophorus* sampling localities (“VNM”: Vietnam, “THA”: Thailand, “MYS”: Malaysia, “JPN”: Japan). The tree was rooted with the outgroup species *Leptopoma vitreum* (OUT001). Specimen codes of individuals with identical COI, 16S and 28S sequences are given at the same branch tip; specimen codes of individuals with identical COI and 16S sequences that are given at different branch tips are labelled with the same superscript number.
(PDF)
