# Supplementary material for: Cryptic diversity of limestone karst inhabiting land snails (Cyclophorus spp.) in northern Vietnam, their evolutionary history and the description of four new species
Source: PLoS One. 2019 Oct 23;14(10):e0222163. doi: 10.1371/journal.pone.0222163 (PMC6808330; doi:10.1371/journal.pone.0222163)
Supplement: S1 Table — Including specimen code and details, locality and sampling information, taxon, mutual group, shell morphology classification and GenBank accession numbers. All material is held at the Natural History Museum, London. Primers used for new COI sequences: 1LCO1490/HCO2198, 2PF372/HCO2198, 3Cyc-F01/HCO2198; primers used for new 28S sequences: 128SF4/28SR5, 228SF4/LSU-4. See S2 Table for VNM001 and VNM029. (PDF) [file pone.0222163.s001.pdf]

**S1 Table. List of studied *Cyclophorus* specimens.** Including specimen code and details, locality and sampling information, taxon, mutual group, shell morphology classification and GenBank accession numbers. All material is held at the Natural History Museum, London. Primers used for new COI sequences: [1]LCO1490/HCO2198, [2]PF372/HCO2198, [3]Cyc-F01/HCO2198; primers used for new 28S sequences: [1]28SF4/28SR5, [2]28SF4/LSU-4. See S2 Table for VNM001 and VNM029.

| Specimen code | Registration no. (NHMUK) | Original sample no. | Specimen code in [6] | Latitude | Longitude | Locality | Collection date | Collector | Taxon | Mutual group | Shell morphology | COI GenBank accession no. | 16S GenBank accession no. | 28S GenBank accession no. | Reference (sequence data) |
|---|---|---|---|---|---|---|---|---|---|---|---|---|---|---|---|
| VNM002 | 20140329/1 | 149(2013)a | 01/02 | 20.29562° | 105.66362° | Vietnam, Ninh Binh, Cuc Phuong National Park | 09.09.2013 | Jonathan Ablett, Hao Van Luong, Fred Naggs & Sang Van Pham | *Cyclophorus* sp. | 2 | deviating | MG720890 | MG720940 | MG720990 | [6] |
| VNM003 | 20140299 | 119(2013)a | 01/01 | 20.35878° | 105.57733° | Vietnam, Ninh Binh, Cuc Phuong National Park | 08.09.2013 | Hao Van Luong & team | *Cyclophorus* sp. | 2 | deviating | MG720888 | MG720938 | MG720988 | [6] |
| VNM004 | 20140509 | 328(2013)a | 01/03 | 20.29358° | 105.66873° | Vietnam, Ninh Binh, Cuc Phuong National Park | 07.09.2013 | Jonathan Ablett, Hao Van Luong, Fred Naggs & Sang Van Pham | *Cyclophorus* sp. | 2 | deviating | MG720895 | MG720945 | MG720993 | [6] |
| VNM005 | 20110513/3 | B31(2011)c | 03/07 | 22.40923° | 105.62855° | Vietnam, Bac Kan, Ba Be National Park | 18.05.2011 | Jonathan Ablett, Hao Van Luong, Fred Naggs & Sang Van Pham | *Cyclophorus fasciatus* Kobelt, 1908 (sub-group I) | 3 | slightly deviating | MG720873 | MG720923 | MG720973 | [6] |
| VNM006 | 20110488/4 | B6(2011)d | | 22.44617° | 105.61740° | Vietnam, Bac Kan, Ba Be National Park | 17.05.2011 | Jonathan Ablett, Hao Van Luong, Fred Naggs & Sang Van Pham | *Cyclophorus fasciatus* Kobelt, 1908 (sub-group I) | 3 | widespread morphotype | MN153300[1] | MN153349 | MN153398[1] | this study |
| VNM007 | 20110513/2 | B31(2011)b | | 22.40923° | 105.62855° | Vietnam, Bac Kan, Ba Be National Park | 18.05.2011 | Jonathan Ablett, Hao Van Luong, Fred Naggs & Sang Van Pham | *Cyclophorus fasciatus* Kobelt, 1908 (sub-group I) | 3 | slightly deviating | MN153301[1] | MN153350 | MN153399[1] | this study |
| VNM008 | 20110513/4 | B31(2011)d | 03/08 | 22.40923° | 105.62855° | Vietnam, Bac Kan, Ba Be National Park | 18.05.2011 | Jonathan Ablett, Hao Van Luong, Fred Naggs & Sang Van Pham | *Cyclophorus fasciatus* Kobelt, 1908 (sub-group I) | 3 | widespread morphotype | MG720874 | MG720924 | MG720974 | [6] |
| VNM009 | 20110488/2 | B6(2011)b | 03/06 | 22.44617° | 105.61740° | Vietnam, Bac Kan, Ba Be National Park | 17.05.2011 | Jonathan Ablett, Hao Van Luong, Fred Naggs & Sang Van Pham | *Cyclophorus fasciatus* Kobelt, 1908 (sub-group I) | 3 | slightly deviating | MG720871 | MG720921 | MG720971 | [6] |
| VNM010 | 20110488/3 | B6(2011)c | | 22.44617° | 105.61740° | Vietnam, Bac Kan, Ba Be National Park | 17.05.2011 | Jonathan Ablett, Hao Van Luong, Fred Naggs & Sang Van Pham | *Cyclophorus fasciatus* Kobelt, 1908 (sub-group I) | 3 | deviating | MN153302[1] | MN153351 | | this study |
| VNM011 | 20180430 | H11(2009)a | | 21.66360° | 106.15095° | Vietnam, Thai Nguyen, Dan Tien commune | 20.05.2009 | Hao Van Luong | *Cyclophorus fasciatus* Kobelt, 1908 (sub-group I) | 3 | deviating | MN153303[1] | MN153352 | MN153400[1] | this study |
| VNM012 | 20180438 | H24(2009)a | | 22.40013° | 105.63267° | Vietnam, Bac Kan, Ba Be National Park | 16.05.2009 | Hao Van Luong | *Cyclophorus fasciatus* Kobelt, 1908 (sub-group I) | 3 | widespread morphotype | MN153304[1] | MN153353 | MN153401[1] | this study |
| VNM013 | 20110476/7 | A5(2011)g | 03/01 | 21.75523° | 105.70745° | Vietnam, Thai Nguyen, Dong Dat commune | 16.05.2011 | Jonathan Ablett, Hao Van Luong, Fred Naggs & Sang Van Pham | *Cyclophorus fasciatus* Kobelt, 1908 (sub-group II) | 4 | widespread morphotype | MG720863 | MG720913 | MG720963 | [6] |
| VNM014 | 20110476/8 | A5(2011)h | 03/02 | 21.75523° | 105.70745° | Vietnam, Thai Nguyen, Dong Dat commune | 16.05.2011 | Jonathan Ablett, Hao Van Luong, Fred Naggs & Sang Van Pham | *Cyclophorus fasciatus* Kobelt, 1908 (sub-group II) | 4 | widespread morphotype | MG720864 | MG720914 | MG720964 | [6] |
| VNM015 | 20110476/9 | A5(2011)i | 03/03 | 21.75523° | 105.70745° | Vietnam, Thai Nguyen, Dong Dat commune | 16.05.2011 | Jonathan Ablett, Hao Van Luong, Fred Naggs & Sang Van Pham | *Cyclophorus fasciatus* Kobelt, 1908 (sub-group II) | 4 | widespread morphotype | MG720865 | MG720915 | MG720965 | [6] |
| VNM016 | 20170411 | VN037 | 04/01 | 21.87870° | 106.54739° | Vietnam, Lang Son, Vinh Lai commune | 20.05.2016 | Tu Van Do, Takahiro Hirano & Takumi Saito | *Cyclophorus* sp. | 5 | deviating | MG720898 | MG720948 | MG720996 | [6] |
| VNM017 | 20170415 | VN042 | 04/02 | 21.72352° | 106.60944° | Vietnam, Lang Son, Mai Sao commune | 18.05.2016 | Tu Van Do, Takahiro Hirano & Takumi Saito | *Cyclophorus* sp. | 6 | deviating | MG720901 | MG720951 | MG720999 | [6] |
| VNM018 | 20110593/3 | E22+E23(2011)c | | 22.13137° | 106.58715° | Vietnam, Lang Son, Trung Quan commune | 23.05.2011 | Hao Van Luong, Fred Naggs & Sang Van Pham | *Cyclophorus courbeti* Ancey, 1888 (sub-group I) | 7 | deviating | MN153305[1] | MN153354 | MN153402[2] | this study |
| VNM019 | 20110591 | E22(2011)a | | 22.13137° | 106.58715° | Vietnam, Lang Son, Trung Quan commune | 23.05.2011 | Hao Van Luong, Fred Naggs & Sang Van Pham | *Cyclophorus courbeti* Ancey, 1888 (sub-group I) | 7 | deviating | MN153306[1] | MN153355 | MN153403[1] | this study |
| VNM020 | 20110592 | E23(2011)a | | 22.13137° | 106.58715° | Vietnam, Lang Son, Trung Quan commune | 23.05.2011 | Hao Van Luong, Fred Naggs & Sang Van Pham | *Cyclophorus courbeti* Ancey, 1888 (sub-group I) | 7 | deviating | MN153307[1] | MN153356 | MN153404[1] | this study |
| VNM021 | 20110540/1 | C19(2011)a | | 22.47878° | 106.04597° | Vietnam, Bac Kan, Bang Van commune | 19.05.2011 | Jonathan Ablett, Hao Van Luong, Fred Naggs & Sang Van Pham | *Cyclophorus courbeti* Ancey, 1888 (sub-group II) | 7 | widespread morphotype | MN153308[1] | MN153357 | MN153405[2] | this study |
| VNM022 | 20110540/2 | C19(2011)b | | 22.47878° | 106.04597° | Vietnam, Bac Kan, Bang Van commune | 19.05.2011 | Jonathan Ablett, Hao Van Luong, Fred Naggs & Sang Van Pham | *Cyclophorus courbeti* Ancey, 1888 (sub-group II) | 7 | widespread morphotype | MN153309[1] | MN153358 | | this study |
| VNM023 | 20110541 | C20(2011)a | | 22.47878° | 106.04597° | Vietnam, Bac Kan, Bang Van commune | 19.05.2011 | Jonathan Ablett, Hao Van Luong, Fred Naggs & Sang Van Pham | *Cyclophorus courbeti* Ancey, 1888 (sub-group II) | 7 | slightly deviating | MN153310[1] | MN153359 | MN153406[2] | this study |
| VNM024 | 20170420 | VN050 | 02/01 | 22.55250° | 106.39639° | Vietnam, Cao Bang, Hong Nam commune | 25.05.2016 | Tu Van Do, Takahiro Hirano & Takumi Saito | *Cyclophorus* sp. | 8 | deviating | MG720904 | MG720954 | MG721002 | [6] |
| VNM025 | 20170413 | VN040 | 02/02 | 22.70617° | 106.28149° | Vietnam, Cao Bang, Nguyen Hue commune | 22.05.2016 | Tu Van Do, Takahiro Hirano & Takumi Saito | *Cyclophorus* sp. | 8 | deviating | MG720899 | MG720949 | MG720997 | [6] |
| VNM026 | 20170414 | VN041 | 02/03 | 22.58867° | 106.35206° | Vietnam, Cao Bang, Hong Nam commune | 25.05.2016 | Tu Van Do, Takahiro Hirano & Takumi Saito | *Cyclophorus* sp. | 8 | deviating | MG720900 | MG720950 | MG720998 | [6] |
| VNM027 | 20130851 | 35,36(2012)a | | 11.45150° | 107.35950° | Vietnam, Dong Nai, Cat Tien National Park | February 2012 | Hao Van Luong, Fred Naggs & Sang Van Pham | *Cyclophorus* sp. | 15 | deviating | MN153311[1] | MN153360 | MN153407[2] | this study |
| VNM028 | 20130872 | 55,56(2012)b | | 11.38953° | 107.34883° | Vietnam, Dong Nai, Cat Tien National Park | February 2012 | Hao Van Luong, Fred Naggs & Sang Van Pham | *Cyclophorus* sp. | 15 | slightly deviating | MN153312[1] | MN153361 | MN153408[2] | this study |
| VNM030 | 20130883 | 75(2012)a | | 17.50317° | 106.26045° | Vietnam, Quang Binh, Phong Nha-Ke Bang National Park | 02.03.2012 | Jonathan Ablett, Hao Van Luong, Fred Naggs & Sang Van Pham | *Cyclophorus phongnhakebangensis* Oheimb, sp. nov. | 37 | slightly deviating | MN153313[1] | MN153362 | MN153409[1] | this study |
| VNM031 | 20130904/3 | 102(2012)c | 06/02 | 17.50317° | 106.26045° | Vietnam, Quang Binh, Phong Nha-Ke Bang National Park | 03.03.2012 | Jonathan Ablett, Hao Van Luong, Fred Naggs & Sang Van Pham | *Cyclophorus phongnhakebangensis* Oheimb, sp. nov. | 37 | slightly deviating | MG720883 | MG720933 | MG720983 | [6] |
| VNM032 | 20130904/4 | 102(2012)d | | 17.50317° | 106.26045° | Vietnam, Quang Binh, Phong Nha-Ke Bang National Park | 03.03.2012 | Jonathan Ablett, Hao Van Luong, Fred Naggs & Sang Van Pham | *Cyclophorus phongnhakebangensis* Oheimb, sp. nov. | 37 | widespread morphotype | MN153314[1] | MN153363 | MN153410[1] | this study |
| VNM033 | 20130915 | 117(2012)a | 06/03 | 17.52843° | 106.27782° | Vietnam, Quang Binh, Phong Nha-Ke Bang National Park | 04.03.2012 | Jonathan Ablett, Hao Van Luong, Fred Naggs & Sang Van Pham | *Cyclophorus phongnhakebangensis* Oheimb, sp. nov. | 37 | deviating | MG720884 | MG720934 | MG720984 | [6] |
| VNM034 | 20130894/1 | 88(2012)a | 06/01 | 17.54147° | 106.23532° | Vietnam, Quang Binh, Phong Nha-Ke Bang National Park | 03.03.2012 | Jonathan Ablett, Hao Van Luong, Fred Naggs & Sang Van Pham | *Cyclophorus phongnhakebangensis* Oheimb, sp. nov. | 37 | slightly deviating | MG720882 | MG720932 | MG720982 | [6] |

| | | | | | | | | | | | | | | | |
|---|---|---|---|---|---|---|---|---|---|---|---|---|---|---|---|
| VNM035 | 20130894/3 | 88(2012)c | | 17.54147° | 106.23532° | Vietnam, Quang Binh, Phong Nha-Ke Bang National Park | 03.03.2012 | Jonathan Ablett, Hao Van Luong, Fred Naggs & Sang Van Pham | *Cyclophorus phongnhakebangensis* Oheimb, sp. nov. | 37 | widespread morphotype | MN153315[1] | MN153364 | MN153411[1] | this study |
| VNM036 | 20180467 | VN249 | | | | Vietnam, Hoa Binh, Cao Duong commune, bought at market stall (20.72542°, 105.64899°), collected nearby according to the merchant | 26.05.2016 (date of purchase) | Tu Van Do, Takahiro Hirano & Takumi Saito (purchasers) | *Cyclophorus* sp. | 38 | deviating | MN153316[1] | MN153365 | MN153412[1] | this study |
| VNM037 | 20180446 | VN060 | | | | Vietnam, Hoa Binh, Cao Duong commune, bought at market stall (20.72542°, 105.64899°), collected nearby according to the merchant | 26.05.2016 (date of purchase) | Tu Van Do, Takahiro Hirano & Takumi Saito (purchasers) | *Cyclophorus* sp. | 38 | deviating | MN153317[1] | MN153366 | MN153413[1] | this study |
| VNM038 | 20140504/1 | 323(2013)a | 08/01 | 20.25038° | 105.71478° | Vietnam, Ninh Binh, Cuc Phuong National Park | 06.09.2013 | Jonathan Ablett, Hao Van Luong, Fred Naggs & Sang Van Pham | *Cyclophorus* sp. | 39 | deviating | MG720894 | MG720944 | MG720992 | [6] |
| VNM039 | 20140378/1 | 198(2013)a | 08/03 | 20.30358° | 105.65478° | Vietnam, Ninh Binh, Cuc Phuong National Park | 10.09.2013 | Jonathan Ablett, Hao Van Luong, Fred Naggs & Sang Van Pham | *Cyclophorus* sp. | 39 | deviating | MG720892 | MG720942 | | [6] |
| VNM040 | 20140330/5 | 150(2013)e | 08/02 | 20.29562° | 105.66362° | Vietnam, Ninh Binh, Cuc Phuong National Park | 09.09.2013 | Jonathan Ablett, Hao Van Luong, Fred Naggs & Sang Van Pham | *Cyclophorus* sp. | 39 | deviating | MG720891 | MG720941 | MG720991 | [6] |
| VNM041 | 20140503/2 | 322(2013)b | 08/05 | 20.27653° | 105.67990° | Vietnam, Ninh Binh, Cuc Phuong National Park | 07.09.2013 | Jonathan Ablett, Hao Van Luong, Fred Naggs & Sang Van Pham | *Cyclophorus* sp. | 39 | deviating | MN153318[1] | MN153367 | MN153414[2] | this study |
| VNM042 | 20110608/3 | G5(2011)c | 07/03 | 20.96638° | 107.16610° | Vietnam, Quang Ninh, Ha Long, Ha Phong ward | 25.05.2011 | Hao Van Luong, Fred Naggs & Sang Van Pham | *Cyclophorus jourdyi* Morlet, 1886 (sub-group I) | 40 | widespread morphotype | MG720878 | MG720928 | MG720978 | [6] |
| VNM043 | 20110608/4 | G5(2011)d | | 20.96638° | 107.16610° | Vietnam, Quang Ninh, Ha Long, Ha Phong ward | 25.05.2011 | Hao Van Luong, Fred Naggs & Sang Van Pham | *Cyclophorus jourdyi* Morlet, 1886 (sub-group I) | 40 | widespread morphotype | MN153319[1] | MN153368 | MN153415[1] | this study |
| VNM044 | 20110608/2 | G5(2011)b | 07/02 | 20.96638° | 107.16610° | Vietnam, Quang Ninh, Ha Long, Ha Phong ward | 25.05.2011 | Hao Van Luong, Fred Naggs & Sang Van Pham | *Cyclophorus jourdyi* Morlet, 1886 (sub-group I) | 40 | widespread morphotype | MG720877 | MG720927 | MG720977 | [6] |
| VNM045 | 20110603/2 | F4(2011)b | 07/01 | 21.06693° | 106.98883° | Vietnam, Quang Ninh, Son Duong commune | 24.05.2011 | Hao Van Luong | *Cyclophorus jourdyi* Morlet, 1886 (sub-group II) | 41 | deviating | MG720876 | MG720926 | MG720976 | [6] |
| VNM046 | 20110603/1 | F4(2011)a | | 21.06693° | 106.98883° | Vietnam, Quang Ninh, Son Duong commune | 24.05.2011 | Hao Van Luong | *Cyclophorus jourdyi* Morlet, 1886 (sub-group II) | 41 | widespread morphotype | MN153320[1] | MN153369 | MN153416[1] | this study |
| VNM047 | 20140306/1 | 126(2013)a | | 20.25053° | 105.71515° | Vietnam, Ninh Binh, Cuc Phuong National Park | 09.09.2013 | Jonathan Ablett, Hao Van Luong, Fred Naggs & Sang Van Pham | *Cyclophorus cucphuongensis* Oheimb, sp. nov. | 42 | widespread morphotype | MN153321[1] | MN153370 | MN153417[1] | this study |
| VNM048 | 20140276/3 | 96(2013)c | | 20.28845° | 105.66767° | Vietnam, Ninh Binh, Cuc Phuong National Park | 07.09.2013 | Hao Van Luong & team | *Cyclophorus cucphuongensis* Oheimb, sp. nov. | 42 | widespread morphotype | MN153322[1] | MN153371 | MN153418[1] | this study |
| VNM049 | 20140306/3 | 126(2013)c | | 20.25053° | 105.71515° | Vietnam, Ninh Binh, Cuc Phuong National Park | 09.09.2013 | Jonathan Ablett, Hao Van Luong, Fred Naggs & Sang Van Pham | *Cyclophorus cucphuongensis* Oheimb, sp. nov. | 42 | widespread morphotype | MN153323[1] | MN153372 | MN153419[1] | this study |
| VNM050 | 20140495 | 314(2013)a | | 20.359° | 105.577° | Vietnam, Ninh Binh, Cuc Phuong National Park | | Hao Van Luong & team | *Cyclophorus cucphuongensis* Oheimb, sp. nov. | 42 | slightly deviating | MN153324[1] | MN153373 | MN153420[1] | this study |
| VNM051 | 20140306/2 | 126(2013)b | 10/03 | 20.25053° | 105.71515° | Vietnam, Ninh Binh, Cuc Phuong National Park | 09.09.2013 | Jonathan Ablett, Hao Van Luong, Fred Naggs & Sang Van Pham | *Cyclophorus cucphuongensis* Oheimb, sp. nov. | 42 | widespread morphotype | MG720889 | MG720939 | MG720989 | [6] |
| VNM052 | 20140276/4 | 96(2013)d | 10/02 | 20.28845° | 105.66767° | Vietnam, Ninh Binh, Cuc Phuong National Park | 07.09.2013 | Hao Van Luong & team | *Cyclophorus cucphuongensis* Oheimb, sp. nov. | 42 | widespread morphotype | MG720887 | MG720937 | MG720987 | [6] |
| VNM053 | 20140287/2 | 107(2013)b | | 20.35548° | 105.63342° | Vietnam, Hoa Binh, Cuc Phuong National Park | 08.09.2013 | Hao Van Luong & team | *Cyclophorus cucphuongensis* Oheimb, sp. nov. | 42 | widespread morphotype | MN153325[1] | MN153374 | MN153421[1] | this study |
| VNM054 | 20140287/3 | 107(2013)c | | 20.35548° | 105.63342° | Vietnam, Hoa Binh, Cuc Phuong National Park | 08.09.2013 | Hao Van Luong & team | *Cyclophorus cucphuongensis* Oheimb, sp. nov. | 42 | widespread morphotype | MN153326[1] | MN153375 | MN153422[1] | this study |
| VNM055 | 20140287/1 | 107(2013)a | | 20.35548° | 105.63342° | Vietnam, Hoa Binh, Cuc Phuong National Park | 08.09.2013 | Hao Van Luong & team | *Cyclophorus cucphuongensis* Oheimb, sp. nov. | 42 | widespread morphotype | MN153327[1] | MN153376 | MN153423[1] | this study |
| VNM056 | 20140494 | 313(2013)a | 10/01 | 20.359° | 105.577° | Vietnam, Ninh Binh, Cuc Phuong National Park | | Hao Van Luong & team | *Cyclophorus cucphuongensis* Oheimb, sp. nov. | 42 | slightly deviating | MG720893 | MG720943 | | [6] |
| VNM057 | 20140497 | 316(2013)a | | 20.359° | 105.577° | Vietnam, Ninh Binh, Cuc Phuong National Park | | Hao Van Luong & team | *Cyclophorus cucphuongensis* Oheimb, sp. nov. | 42 | widespread morphotype | MN153328[1] | MN153377 | MN153424[1] | this study |
| VNM058 | 20110553/2 | D9(2011)b | | 22.85068° | 106.72625° | Vietnam, Cao Bang, Dam Thuy commune | 21.05.2011 | Jonathan Ablett, Hao Van Luong, Fred Naggs & Sang Van Pham | *Cyclophorus* sp. | 43 | deviating | MN153329[1] | MN153378 | MN153425[2] | this study |
| VNM059 | 20140557/2 | 376(2013)b | | 21.66235°–21.65998° | 106.36633°–106.36462° | Vietnam, Lang Son, Huu Lien commune, Huu Lien Nature Reserve | 17.09.2013 | Jonathan Ablett, Hao Van Luong, Fred Naggs & Sang Van Pham | *Cyclophorus subfloridus* Ancey, 1888 (sub-group I) | 44 | widespread morphotype | MN153330[1] | MN153379 | MN153426[1] | this study |
| VNM060 | 20140584 | 403(2013)a | | 21.65948° | 106.36535° | Vietnam, Lang Son, Yen Thinh commune | 18.09.2013 | Jonathan Ablett, Hao Van Luong, Fred Naggs & Sang Van Pham | *Cyclophorus subfloridus* Ancey, 1888 (sub-group I) | 44 | widespread morphotype | MN153331[1] | MN153380 | MN153427[1] | this study |
| VNM061 | 20140585/1 | 404(2013)a | | 21.65948° | 106.36535° | Vietnam, Lang Son, Yen Thinh commune | 18.09.2013 | Jonathan Ablett, Hao Van Luong, Fred Naggs & Sang Van Pham | *Cyclophorus subfloridus* Ancey, 1888 (sub-group I) | 44 | widespread morphotype | MN153332[1] | MN153381 | MN153428[1] | this study |
| VNM062 | 20140596 | 415(2013)a | | 21.61862° | 106.34760° | Vietnam, Lang Son, Yen Thinh commune | 19.09.2013 | Jonathan Ablett, Hao Van Luong, Fred Naggs & Sang Van Pham | *Cyclophorus subfloridus* Ancey, 1888 (sub-group I) | 44 | widespread morphotype | MN153333[1] | MN153382 | MN153429[1] | this study |
| VNM063 | 20140597 | 416(2013)a | | 21.61862° | 106.34760° | Vietnam, Lang Son, Yen Thinh commune | 19.09.2013 | Jonathan Ablett, Hao Van Luong, Fred Naggs & Sang Van Pham | *Cyclophorus subfloridus* Ancey, 1888 (sub-group I) | 44 | widespread morphotype | MN153334[1] | MN153383 | MN153430[1] | this study |
| VNM064 | 20140595 | 414(2013)a | | 21.61862° | 106.34760° | Vietnam, Lang Son, Yen Thinh commune | 19.09.2013 | Jonathan Ablett, Hao Van Luong, Fred Naggs & Sang Van Pham | *Cyclophorus subfloridus* Ancey, 1888 (sub-group I) | 44 | widespread morphotype | MN153335[1] | MN153384 | MN153431[1] | this study |
| VNM065 | 20110594 | E24(2011)a | | 22.13137° | 106.58715° | Vietnam, Lang Son, Trung Quan commune | 23.05.2011 | Hao Van Luong, Fred Naggs & Sang Van Pham | *Cyclophorus subfloridus* Ancey, 1888 (sub-group II) | 45 | slightly deviating | MN153336[1] | MN153385 | MN153432[1] | this study |
| VNM066 | 20110476/12 | A5(2011)l | 09/02 | 21.75523° | 105.70745° | Vietnam, Thai Nguyen, Dong Dat commune | 16.05.2011 | Jonathan Ablett, Hao Van Luong, Fred Naggs & Sang Van Pham | *Cyclophorus* sp. | 46 | deviating | MG720867 | MG720917 | MG720967 | [6] |
| VNM067 | 20110476/10 | A5(2011)j | 09/03 | 21.75523° | 105.70745° | Vietnam, Thai Nguyen, Dong Dat commune | 16.05.2011 | Jonathan Ablett, Hao Van Luong, Fred Naggs & Sang Van Pham | *Cyclophorus* sp. | 46 | deviating | MG720866 | MG720916 | MG720966 | [6] |
| VNM068 | 20170403 | H9(2009)a | 09/01 | 21.75510° | 105.70730° | Vietnam, Thai Nguyen, Dong Dat commune | 14.05.2009 | Hao Van Luong | *Cyclophorus* sp. | 46 | deviating | MG720861 | MG720911 | MG720961 | [6] |
| VNM069 | 20180432 | H15(2009)j | | 20.35583° | 105.51088° | Vietnam, Thanh Hoa, Cuc Phuong National Park | 02.05.2009 | Hao Van Luong | *Cyclophorus paracucphuongensis* Oheimb, sp. nov. | 47 | widespread morphotype | MN153337[2] | MN153386 | MN153433[1] | this study |

| | | | | | | | | | | | | | | | |
|---|---|---|---|---|---|---|---|---|---|---|---|---|---|---|---|
| VNM070 | 20180434 | H15(2009)l | | 20.35583° | 105.51088° | Vietnam, Thanh Hoa, Cuc Phuong National Park | 02.05.2009 | Hao Van Luong | *Cyclophorus paracucphuongensis* Oheimb, sp. nov. | 47 | widespread morphotype | MN153338[2] | MN153387 | MN153434[1] | this study |
| VNM071 | 20180433 | H15(2009)k | | 20.35583° | 105.51088° | Vietnam, Thanh Hoa, Cuc Phuong National Park | 02.05.2009 | Hao Van Luong | *Cyclophorus paracucphuongensis* Oheimb, sp. nov. | 47 | widespread morphotype | MN153339[3] | MN153388 | MN153435[1] | this study |
| VNM072 | 20180466 | VN248 | | | | Vietnam, Hoa Binh, Cao Duong commune, bought at market stall (20.72542°, 105.64899°), collected nearby according to the merchant | 26.05.2016 (date of purchase) | Tu Van Do, Takahiro Hirano & Takumi Saito (purchasers) | *Cyclophorus* sp. 1 | 48 | widespread morphotype | MN153340[1] | MN153389 | MN153436[1] | this study |
| VNM073 | 20180448 | VN065 | | | | Vietnam, Hoa Binh, Cao Duong commune, bought at market stall (20.72542°, 105.64899°), collected nearby according to the merchant | 26.05.2016 (date of purchase) | Tu Van Do, Takahiro Hirano & Takumi Saito (purchasers) | *Cyclophorus* sp. 1 | 48 | widespread morphotype | MN153341[1] | MN153390 | MN153437[1] | this study |
| VNM074 | 20180447 | VN064 | | | | Vietnam, Hoa Binh, Cao Duong commune, bought at market stall (20.72542°, 105.64899°), collected nearby according to the merchant | 26.05.2016 (date of purchase) | Tu Van Do, Takahiro Hirano & Takumi Saito (purchasers) | *Cyclophorus* sp. 1 | 48 | widespread morphotype | MN153342[1] | MN153391 | MN153438[1] | this study |
| VNM075 | 20180452 | VN069 | | | | Vietnam, Hoa Binh, Cao Duong commune, bought at market stall (20.72542°, 105.64899°), collected nearby according to the merchant | 26.05.2016 (date of purchase) | Tu Van Do, Takahiro Hirano & Takumi Saito (purchasers) | *Cyclophorus* sp. 1 | 48 | widespread morphotype | MN153343[1] | MN153392 | MN153439[1] | this study |
| VNM076 | 20180455 | VN081 | | 20.38493° | 105.52280° | Vietnam, Hoa Binh, Cuc Phuong National Park | 26.05.2016 | Tu Van Do, Takahiro Hirano & Takumi Saito | *Cyclophorus takumisaitoi* Hirano, sp. nov. | 49 | widespread morphotype | MN153344[1] | MN153393 | MN153440[1] | this study |
| VNM077 | 20180457 | VN083 | | 20.38493° | 105.52280° | Vietnam, Hoa Binh, Cuc Phuong National Park | 26.05.2016 | Tu Van Do, Takahiro Hirano & Takumi Saito | *Cyclophorus takumisaitoi* Hirano, sp. nov. | 49 | widespread morphotype | MN153345[1] | MN153394 | MN153441[1] | this study |
| VNM078 | 20180456 | VN082 | | 20.38493° | 105.52280° | Vietnam, Hoa Binh, Cuc Phuong National Park | 26.05.2016 | Tu Van Do, Takahiro Hirano & Takumi Saito | *Cyclophorus takumisaitoi* Hirano, sp. nov. | 49 | slightly deviating | MN153346[1] | MN153395 | MN153442[1] | this study |
| VNM079 | 20170424 | VN074 | 11/02 | 21.97243° | 106.31021° | Vietnam, Lang Son, Mong An commune | 20.05.2016 | Tu Van Do, Takahiro Hirano & Takumi Saito | *Cyclophorus* sp. | 50 | deviating | MG720908 | MG720958 | MG721006 | [6] |
| VNM080 | 20140559/1 | 378(2013)a | | 21.66235°–21.65998° | 106.36633°–106.36462° | Vietnam, Lang Son, Huu Lien commune, Huu Lien Nature Reserve | 17.09.2013 | Jonathan Ablett, Hao Van Luong, Fred Naggs & Sang Van Pham | *Cyclophorus* sp. | 51 | slightly deviating | MN153347[1] | MN153396 | MN153443[1] | this study |
| VNM081 | 20180431 | H12(2009)a | | 22.77595° | 104.97278° | Vietnam, Ha Giang, Phuong Thien commune | 18.05.2009 | Hao Van Luong | *Cyclophorus* sp. | 52 | deviating | MN153348[1] | MN153397 | MN153444[1] | this study |
| VNM082 | 20170423 | VN073 | 11/01 | 21.71884° | 106.61270° | Vietnam, Lang Son, Mai Sao commune | 18.05.2016 | Tu Van Do, Takahiro Hirano & Takumi Saito | *Cyclophorus* sp. | 53 | deviating | MG720907 | MG720957 | MG721005 | [6] |
| VNM083 | 20110609/5 | G6(2011)e | 05/01 | 20.96638° | 107.16610° | Vietnam, Quang Ninh, Ha Long, Ha Phong ward | 25.05.2011 | Hao Van Luong, Fred Naggs & Sang Van Pham | *Cyclophorus* sp. | 56 | deviating | MG720881 | MG720931 | MG720981 | [6] |
| VNM084 | 20110609/1 | G6(2011)a | 05/02 | 20.96638° | 107.16610° | Vietnam, Quang Ninh, Ha Long, Ha Phong ward | 25.05.2011 | Hao Van Luong, Fred Naggs & Sang Van Pham | *Cyclophorus* sp. | 56 | deviating | MG720879 | MG720929 | MG720979 | [6] |
| VNM085 | 20110609/4 | G6(2011)d | 05/03 | 20.96638° | 107.16610° | Vietnam, Quang Ninh, Ha Long, Ha Phong ward | 25.05.2011 | Hao Van Luong, Fred Naggs & Sang Van Pham | *Cyclophorus* sp. | 56 | deviating | MG720880 | MG720930 | MG720980 | [6] |
| VNM086 | 20130927 | 132(2012)a | 14/01 | 17.52843° | 106.27782° | Vietnam, Quang Binh, Phong Nha-Ke Bang National Park | 04.03.2012 | Jonathan Ablett, Hao Van Luong, Fred Naggs & Sang Van Pham | *Cyclophorus* sp. | 57 | deviating | MG720885 | MG720935 | MG720985 | [6] |
| VNM087 | 20130945/3 | 157(2012)c | 14/02 | 17.50317° | 106.26045° | Vietnam, Quang Binh, Phong Nha-Ke Bang National Park | 05.03.2012 | Jonathan Ablett, Hao Van Luong, Fred Naggs & Sang Van Pham | *Cyclophorus* sp. | 57 | deviating | MG720886 | MG720936 | MG720986 | [6] |
| VNM088 | 20110481/2 | A10(2011)b | 13/06 | 21.75523° | 105.70745° | Vietnam, Thai Nguyen, Dong Dat commune | 16.05.2011 | Jonathan Ablett, Hao Van Luong, Fred Naggs & Sang Van Pham | *Cyclophorus* sp. | 58 | deviating | MG720868 | MG720918 | MG720968 | [6] |
| VNM089 | 20110490/2 | B8(2011)b | 13/03 | 22.44617° | 105.61740° | Vietnam, Bac Kan, Ba Be National Park | 17.05.2011 | Jonathan Ablett, Hao Van Luong, Fred Naggs & Sang Van Pham | *Cyclophorus* sp. | 58 | deviating | MG720872 | MG720922 | MG720972 | [6] |
| VNM090 | 20110481/3 | A10(2011)c | 13/07 | 21.75523° | 105.70745° | Vietnam, Thai Nguyen, Dong Dat commune | 16.05.2011 | Jonathan Ablett, Hao Van Luong, Fred Naggs & Sang Van Pham | *Cyclophorus* sp. | 58 | deviating | MG720869 | MG720919 | MG720969 | [6] |
| VNM091 | 20110481/4 | A10(2011)d | 13/08 | 21.75523° | 105.70745° | Vietnam, Thai Nguyen, Dong Dat commune | 16.05.2011 | Jonathan Ablett, Hao Van Luong, Fred Naggs & Sang Van Pham | *Cyclophorus* sp. | 58 | deviating | MG720870 | MG720920 | MG720970 | [6] |
| VNM092 | 20110515/3 | B33(2011)c | 13/02 | 22.40923° | 105.62855° | Vietnam, Bac Kan, Ba Be National Park | 18.05.2011 | Jonathan Ablett, Hao Van Luong, Fred Naggs & Sang Van Pham | *Cyclophorus* sp. | 59 | deviating | MG720875 | MG720925 | MG720975 | [6] |
| VNM093 | 20170404 | H31(2009)a | 13/01 | 22.41638° | 105.63172° | Vietnam, Bac Kan, Ba Be National Park | 16.05.2009 | Hao Van Luong | *Cyclophorus* sp. | 59 | deviating | MG720862 | MG720912 | MG720962 | [6] |
| VNM094 | 20160724 | V159(2008)a | 12/01 | 20.8° | 107.0° | Vietnam, Hai Phong, Cat Ba National Park | 29.05.2008 | Hao Van Luong & Fred Naggs | *Cyclophorus* sp. | 60 | deviating | MG720859 | MG720909 | MG720959 | [6] |
| VNM095 | 20160725 | V159(2008)b | 12/02 | 20.8° | 107.0° | Vietnam, Hai Phong, Cat Ba National Park | 29.05.2008 | Hao Van Luong & Fred Naggs | *Cyclophorus* sp. | 60 | deviating | MG720860 | MG720910 | MG720960 | [6] |
| VNM096 | 20170410 | VN036 | 12/07 | 22.6781° | 106.5228° | Vietnam, Cao Bang, Cai Bo commune | 23.05.2016 | Tu Van Do | *Cyclophorus* sp. | 61 | deviating | MG720897 | MG720947 | MG720995 | [6] |
| VNM097 | 20170407 | VN004 | 12/06 | 22.6781° | 106.5228° | Vietnam, Cao Bang, Cai Bo commune | 23.05.2016 | Tu Van Do | *Cyclophorus* sp. | 61 | deviating | MG720896 | MG720946 | MG720994 | [6] |
| VNM098 | 20170419 | VN046 | 12/05 | 22.67799° | 106.52164° | Vietnam, Cao Bang, Cai Bo commune | 23.05.2016 | Tu Van Do, Takahiro Hirano & Takumi Saito | *Cyclophorus* sp. | 61 | deviating | MG720903 | MG720953 | MG721001 | [6] |
| VNM099 | 20170421 | VN071 | 12/10 | 21.71884° | 106.61270° | Vietnam, Lang Son, Mai Sao commune | 18.05.2016 | Tu Van Do, Takahiro Hirano & Takumi Saito | *Cyclophorus* sp. | 62 | deviating | MG720905 | MG720955 | MG721003 | [6] |
| VNM100 | 20170418 | VN045 | 12/11 | 21.72352° | 106.60944° | Vietnam, Lang Son, Mai Sao commune | 18.05.2016 | Tu Van Do, Takahiro Hirano & Takumi Saito | *Cyclophorus* sp. | 62 | deviating | MG720902 | MG720952 | MG721000 | [6] |
| VNM101 | 20170422 | VN072 | 12/12 | 21.71884° | 106.61270° | Vietnam, Lang Son, Mai Sao commune | 18.05.2016 | Tu Van Do, Takahiro Hirano & Takumi Saito | *Cyclophorus* sp. | 62 | deviating | MG720906 | MG720956 | MG721004 | [6] |
| VNM102 | 20170428 | VN106 | 03/10 | 22.44685° | 105.61720° | Vietnam, Bac Kan, Ba Be National Park | 15.05.2009 | Hao Van Luong | *Cyclophorus fasciatus* Kobelt, 1908 (sub-group I) | | deviating | | | | |

| | | | | | | | | | | | | | | | |
|---|---|---|---|---|---|---|---|---|---|---|---|---|---|---|---|
| VNM103 | 20180461 | VN129 | | 21.66360° | 106.15095° | Vietnam, Thai Nguyen, Dan Tien commune | 20.05.2009 | Hao Van Luong | *Cyclophorus fasciatus* Kobelt, 1908 (sub-group I) | | widespread morphotype | | | | |
| VNM104 | 20170405 | A(2011)h | 03/04 | 21.75523° | 105.70745° | Vietnam, Thai Nguyen, Dong Dat commune | 16.05.2011 | Jonathan Ablett, Hao Van Luong, Fred Naggs & Sang Van Pham | *Cyclophorus fasciatus* Kobelt, 1908 (sub-group II) | | widespread morphotype | | | | |
| VNM105 | 20110476/11 | A5(2011)k | 03/05 | 21.75523° | 105.70745° | Vietnam, Thai Nguyen, Dong Dat commune | 16.05.2011 | Jonathan Ablett, Hao Van Luong, Fred Naggs & Sang Van Pham | *Cyclophorus fasciatus* Kobelt, 1908 (sub-group II) | | widespread morphotype | | | | |
| VNM106 | 20110476/15 | A5(2011)o | | 21.75523° | 105.70745° | Vietnam, Thai Nguyen, Dong Dat commune | 16.05.2011 | Jonathan Ablett, Hao Van Luong, Fred Naggs & Sang Van Pham | *Cyclophorus fasciatus* Kobelt, 1908 (sub-group II) | | widespread morphotype | | | | |
| VNM107 | 20110476/18 | A5(2011)r | | 21.75523° | 105.70745° | Vietnam, Thai Nguyen, Dong Dat commune | 16.05.2011 | Jonathan Ablett, Hao Van Luong, Fred Naggs & Sang Van Pham | *Cyclophorus fasciatus* Kobelt, 1908 (sub-group II) | | widespread morphotype | | | | |
| VNM108 | 20180462 | VN175 | | 21.75510° | 105.70730° | Vietnam, Thai Nguyen, Dong Dat commune | 14.05.2009 | Hao Van Luong | *Cyclophorus fasciatus* Kobelt, 1908 (sub-group II) | | widespread morphotype | | | | |
| VNM109 | 20180463 | VN178 | | 21.75510° | 105.70730° | Vietnam, Thai Nguyen, Dong Dat commune | 14.05.2009 | Hao Van Luong | *Cyclophorus fasciatus* Kobelt, 1908 (sub-group II) | | slightly deviating | | | | |
| VNM110 | 20180464 | VN192 | | 21.75510° | 105.70730° | Vietnam, Thai Nguyen, Dong Dat commune | 14.05.2009 | Hao Van Luong | *Cyclophorus fasciatus* Kobelt, 1908 (sub-group II) | | widespread morphotype | | | | |
| VNM111 | 20110582/31 | EB(2011)ae | | 22.13137° | 106.58715° | Vietnam, Lang Son, Trung Quan commune | 23.05.2011 | Hao Van Luong, Fred Naggs & Sang Van Pham | *Cyclophorus courbeti* Ancey, 1888 (sub-group I) | | deviating | | | | |
| VNM112 | 20110582/32 | EB(2011)af | | 22.13137° | 106.58715° | Vietnam, Lang Son, Trung Quan commune | 23.05.2011 | Hao Van Luong, Fred Naggs & Sang Van Pham | *Cyclophorus courbeti* Ancey, 1888 (sub-group I) | | deviating | | | | |
| VNM113 | 20110582/33 | EB(2011)ag | | 22.13137° | 106.58715° | Vietnam, Lang Son, Trung Quan commune | 23.05.2011 | Hao Van Luong, Fred Naggs & Sang Van Pham | *Cyclophorus courbeti* Ancey, 1888 (sub-group I) | | deviating | | | | |
| VNM114 | 20110582/34 | EB(2011)ah | | 22.13137° | 106.58715° | Vietnam, Lang Son, Trung Quan commune | 23.05.2011 | Hao Van Luong, Fred Naggs & Sang Van Pham | *Cyclophorus courbeti* Ancey, 1888 (sub-group I) | | deviating | | | | |
| VNM115 | 20110593/1 | E22+E23(2011)a | | 22.13137° | 106.58715° | Vietnam, Lang Son, Trung Quan commune | 23.05.2011 | Hao Van Luong, Fred Naggs & Sang Van Pham | *Cyclophorus courbeti* Ancey, 1888 (sub-group I) | | deviating | | | | |
| VNM116 | 20110593/2 | E22+E23(2011)b | | 22.13137° | 106.58715° | Vietnam, Lang Son, Trung Quan commune | 23.05.2011 | Hao Van Luong, Fred Naggs & Sang Van Pham | *Cyclophorus courbeti* Ancey, 1888 (sub-group I) | | slightly deviating | | | | |
| VNM117 | 20110593/4 | E22+E23(2011)d | | 22.13137° | 106.58715° | Vietnam, Lang Son, Trung Quan commune | 23.05.2011 | Hao Van Luong, Fred Naggs & Sang Van Pham | *Cyclophorus courbeti* Ancey, 1888 (sub-group I) | | deviating | | | | |
| VNM118 | 20110593/5 | E22+E23(2011)e | | 22.13137° | 106.58715° | Vietnam, Lang Son, Trung Quan commune | 23.05.2011 | Hao Van Luong, Fred Naggs & Sang Van Pham | *Cyclophorus courbeti* Ancey, 1888 (sub-group I) | | deviating | | | | |
| VNM119 | 20110593/6 | E22+E23(2011)f | | 22.13137° | 106.58715° | Vietnam, Lang Son, Trung Quan commune | 23.05.2011 | Hao Van Luong, Fred Naggs & Sang Van Pham | *Cyclophorus courbeti* Ancey, 1888 (sub-group I) | | deviating | | | | |
| VNM120 | 20110593/7 | E22+E23(2011)g | | 22.13137° | 106.58715° | Vietnam, Lang Son, Trung Quan commune | 23.05.2011 | Hao Van Luong, Fred Naggs & Sang Van Pham | *Cyclophorus courbeti* Ancey, 1888 (sub-group I) | | deviating | | | | |
| VNM121 | 20180459 | VN088 | | 22.47878° | 106.04597° | Vietnam, Bac Kan, Bang Van commune | 19.05.2011 | Jonathan Ablett, Hao Van Luong, Fred Naggs & Sang Van Pham | *Cyclophorus courbeti* Ancey, 1888 (sub-group II) | | slightly deviating | | | | |
| VNM122 | 20180460 | VN089 | | 22.47878° | 106.04597° | Vietnam, Bac Kan, Bang Van commune | 19.05.2011 | Jonathan Ablett, Hao Van Luong, Fred Naggs & Sang Van Pham | *Cyclophorus courbeti* Ancey, 1888 (sub-group II) | | slightly deviating | | | | |
| VNM123 | 20180468 | VN327 | | 22.47878° | 106.04597° | Vietnam, Bac Kan, Bang Van commune | 19.05.2011 | Jonathan Ablett, Hao Van Luong, Fred Naggs & Sang Van Pham | *Cyclophorus courbeti* Ancey, 1888 (sub-group II) | | deviating | | | | |
| VNM124 | 20180469 | VN328 | | 22.47878° | 106.04597° | Vietnam, Bac Kan, Bang Van commune | 19.05.2011 | Jonathan Ablett, Hao Van Luong, Fred Naggs & Sang Van Pham | *Cyclophorus courbeti* Ancey, 1888 (sub-group II) | | widespread morphotype | | | | |
| VNM125 | 20130921/2 | 126(2012)b | 06/04 | 17.50317° | 106.26045° | Vietnam, Quang Binh, Phong Nha-Ke Bang National Park | 04.03.2012 | Jonathan Ablett, Hao Van Luong, Fred Naggs & Sang Van Pham | *Cyclophorus phongnhakebangensis* Oheimb, sp. nov. | | widespread morphotype | | | | |
| VNM126 | 20130942/2 | 154(2012)b | 06/05 | 17.50317° | 106.26045° | Vietnam, Quang Binh, Phong Nha-Ke Bang National Park | 05.03.2012 | Jonathan Ablett, Hao Van Luong, Fred Naggs & Sang Van Pham | *Cyclophorus phongnhakebangensis* Oheimb, sp. nov. | | widespread morphotype | | | | |
| VNM127 | 20130943/1 | 155(2012)a | | 17.50317° | 106.26045° | Vietnam, Quang Binh, Phong Nha-Ke Bang National Park | 05.03.2012 | Jonathan Ablett, Hao Van Luong, Fred Naggs & Sang Van Pham | *Cyclophorus phongnhakebangensis* Oheimb, sp. nov. | | widespread morphotype | | | | |
| VNM128 | 20130945/2 | 157(2012)b | | 17.50317° | 106.26045° | Vietnam, Quang Binh, Phong Nha-Ke Bang National Park | 05.03.2012 | Jonathan Ablett, Hao Van Luong, Fred Naggs & Sang Van Pham | *Cyclophorus phongnhakebangensis* Oheimb, sp. nov. | | widespread morphotype | | | | |
| VNM129 | 20130945/4 | 157(2012)d | | 17.50317° | 106.26045° | Vietnam, Quang Binh, Phong Nha-Ke Bang National Park | 05.03.2012 | Jonathan Ablett, Hao Van Luong, Fred Naggs & Sang Van Pham | *Cyclophorus phongnhakebangensis* Oheimb, sp. nov. | | widespread morphotype | | | | |
| VNM130 | 20130945/5 | 157(2012)e | | 17.50317° | 106.26045° | Vietnam, Quang Binh, Phong Nha-Ke Bang National Park | 05.03.2012 | Jonathan Ablett, Hao Van Luong, Fred Naggs & Sang Van Pham | *Cyclophorus phongnhakebangensis* Oheimb, sp. nov. | | widespread morphotype | | | | |
| VNM131 | 20130945/6 | 157(2012)f | | 17.50317° | 106.26045° | Vietnam, Quang Binh, Phong Nha-Ke Bang National Park | 05.03.2012 | Jonathan Ablett, Hao Van Luong, Fred Naggs & Sang Van Pham | *Cyclophorus phongnhakebangensis* Oheimb, sp. nov. | | widespread morphotype | | | | |
| VNM132 | 20130945/7 | 157(2012)g | | 17.50317° | 106.26045° | Vietnam, Quang Binh, Phong Nha-Ke Bang National Park | 05.03.2012 | Jonathan Ablett, Hao Van Luong, Fred Naggs & Sang Van Pham | *Cyclophorus phongnhakebangensis* Oheimb, sp. nov. | | widespread morphotype | | | | |
| VNM133 | 20180440 | 83(2012)c | | 17.54147° | 106.23532° | Vietnam, Quang Binh, Phong Nha-Ke Bang National Park | 03.03.2012 | Jonathan Ablett, Hao Van Luong, Fred Naggs & Sang Van Pham | *Cyclophorus phongnhakebangensis* Oheimb, sp. nov. | | widespread morphotype | | | | |
| VNM134 | 20180441 | 83(2012)d | | 17.54147° | 106.23532° | Vietnam, Quang Binh, Phong Nha-Ke Bang National Park | 03.03.2012 | Jonathan Ablett, Hao Van Luong, Fred Naggs & Sang Van Pham | *Cyclophorus phongnhakebangensis* Oheimb, sp. nov. | | widespread morphotype | | | | |
| VNM135 | 20180442 | 83(2012)e | | 17.54147° | 106.23532° | Vietnam, Quang Binh, Phong Nha-Ke Bang National Park | 03.03.2012 | Jonathan Ablett, Hao Van Luong, Fred Naggs & Sang Van Pham | *Cyclophorus phongnhakebangensis* Oheimb, sp. nov. | | widespread morphotype | | | | |
| VNM136 | 20180443 | 83(2012)f | | 17.54147° | 106.23532° | Vietnam, Quang Binh, Phong Nha-Ke Bang National Park | 03.03.2012 | Jonathan Ablett, Hao Van Luong, Fred Naggs & Sang Van Pham | *Cyclophorus phongnhakebangensis* Oheimb, sp. nov. | | widespread morphotype | | | | |
| VNM137 | 20180444 | 83(2012)g | | 17.54147° | 106.23532° | Vietnam, Quang Binh, Phong Nha-Ke Bang National Park | 03.03.2012 | Jonathan Ablett, Hao Van Luong, Fred Naggs & Sang Van Pham | *Cyclophorus phongnhakebangensis* Oheimb, sp. nov. | | widespread morphotype | | | | |
| VNM138 | 20180465 | VN198 | | 17.533749° | 106.280220° | Vietnam, Quang Binh, Phong Nha-Ke Bang National Park | 07.05.2008 | Hao Van Luong | *Cyclophorus phongnhakebangensis* Oheimb, sp. nov. | | deviating | | | | |
| VNM139 | 20110621/22 | GA(2011)v | | 20.96638° | 107.16610° | Vietnam, Quang Ninh, Ha Long, Ha Phong ward | 25.05.2011 | Hao Van Luong, Fred Naggs & Sang Van Pham | *Cyclophorus jourdyi* Morlet, 1886 (sub-group I) | | widespread morphotype | | | | |

| | | | | | | | | | | | | | | | |
|---|---|---|---|---|---|---|---|---|---|---|---|---|---|---|---|
| VNM140 | 20110621/23 | GA(2011)w | | 20.96638° | 107.16610° | Vietnam, Quang Ninh, Ha Long, Ha Phong ward | 25.05.2011 | Hao Van Luong, Fred Naggs & Sang Van Pham | *Cyclophorus jourdyi* Morlet, 1886 (sub-group I) | | widespread morphotype | | | | |
| VNM141 | 20110621/24 | GA(2011)x | | 20.96638° | 107.16610° | Vietnam, Quang Ninh, Ha Long, Ha Phong ward | 25.05.2011 | Hao Van Luong, Fred Naggs & Sang Van Pham | *Cyclophorus jourdyi* Morlet, 1886 (sub-group I) | | widespread morphotype | | | | |
| VNM142 | 20110621/25 | GA(2011)y | | 20.96638° | 107.16610° | Vietnam, Quang Ninh, Ha Long, Ha Phong ward | 25.05.2011 | Hao Van Luong, Fred Naggs & Sang Van Pham | *Cyclophorus jourdyi* Morlet, 1886 (sub-group I) | | widespread morphotype | | | | |
| VNM143 | 20110621/26 | GA(2011)z | | 20.96638° | 107.16610° | Vietnam, Quang Ninh, Ha Long, Ha Phong ward | 25.05.2011 | Hao Van Luong, Fred Naggs & Sang Van Pham | *Cyclophorus jourdyi* Morlet, 1886 (sub-group I) | | widespread morphotype | | | | |
| VNM144 | 20110621/27 | GA(2011)aa | | 20.96638° | 107.16610° | Vietnam, Quang Ninh, Ha Long, Ha Phong ward | 25.05.2011 | Hao Van Luong, Fred Naggs & Sang Van Pham | *Cyclophorus jourdyi* Morlet, 1886 (sub-group I) | | widespread morphotype | | | | |
| VNM145 | 20110608/10 | G5(2011)j | | 20.96638° | 107.16610° | Vietnam, Quang Ninh, Ha Long, Ha Phong ward | 25.05.2011 | Hao Van Luong, Fred Naggs & Sang Van Pham | *Cyclophorus jourdyi* Morlet, 1886 (sub-group I) | | widespread morphotype | | | | |
| VNM146 | 20110608/11 | G5(2011)k | | 20.96638° | 107.16610° | Vietnam, Quang Ninh, Ha Long, Ha Phong ward | 25.05.2011 | Hao Van Luong, Fred Naggs & Sang Van Pham | *Cyclophorus jourdyi* Morlet, 1886 (sub-group I) | | widespread morphotype | | | | |
| VNM147 | 20110608/12 | G5(2011)l | | 20.96638° | 107.16610° | Vietnam, Quang Ninh, Ha Long, Ha Phong ward | 25.05.2011 | Hao Van Luong, Fred Naggs & Sang Van Pham | *Cyclophorus jourdyi* Morlet, 1886 (sub-group I) | | slightly deviating | | | | |
| VNM148 | 20110608/5 | G5(2011)e | | 20.96638° | 107.16610° | Vietnam, Quang Ninh, Ha Long, Ha Phong ward | 25.05.2011 | Hao Van Luong, Fred Naggs & Sang Van Pham | *Cyclophorus jourdyi* Morlet, 1886 (sub-group I) | | widespread morphotype | | | | |
| VNM149 | 20110608/6 | G5(2011)f | | 20.96638° | 107.16610° | Vietnam, Quang Ninh, Ha Long, Ha Phong ward | 25.05.2011 | Hao Van Luong, Fred Naggs & Sang Van Pham | *Cyclophorus jourdyi* Morlet, 1886 (sub-group I) | | widespread morphotype | | | | |
| VNM150 | 20110608/7 | G5(2011)g | | 20.96638° | 107.16610° | Vietnam, Quang Ninh, Ha Long, Ha Phong ward | 25.05.2011 | Hao Van Luong, Fred Naggs & Sang Van Pham | *Cyclophorus jourdyi* Morlet, 1886 (sub-group I) | | widespread morphotype | | | | |
| VNM151 | 20110608/8 | G5(2011)h | | 20.96638° | 107.16610° | Vietnam, Quang Ninh, Ha Long, Ha Phong ward | 25.05.2011 | Hao Van Luong, Fred Naggs & Sang Van Pham | *Cyclophorus jourdyi* Morlet, 1886 (sub-group I) | | widespread morphotype | | | | |
| VNM152 | 20110608/9 | G5(2011)i | | 20.96638° | 107.16610° | Vietnam, Quang Ninh, Ha Long, Ha Phong ward | 25.05.2011 | Hao Van Luong, Fred Naggs & Sang Van Pham | *Cyclophorus jourdyi* Morlet, 1886 (sub-group I) | | widespread morphotype | | | | |
| VNM153 | 20180445 | VN033 | | 21.06693° | 106.98883° | Vietnam, Quang Ninh, Son Duong commune | 24.05.2011 | Hao Van Luong | *Cyclophorus jourdyi* Morlet, 1886 (sub-group II) | | slightly deviating | | | | |
| VNM154 | 20140360/1 | 180(2013)a | | 20.35038° | 105.60360° | Vietnam, Ninh Binh, Cuc Phuong National Park | 10.09.2013 | Jonathan Ablett, Hao Van Luong, Fred Naggs & Sang Van Pham | *Cyclophorus cucphuongensis* Oheimb, sp. nov. | | widespread morphotype | | | | |
| VNM155 | 20140377/2 | 197(2013)b | | 20.30358° | 105.65478° | Vietnam, Ninh Binh, Cuc Phuong National Park | 10.09.2013 | Jonathan Ablett, Hao Van Luong, Fred Naggs & Sang Van Pham | *Cyclophorus cucphuongensis* Oheimb, sp. nov. | | widespread morphotype | | | | |
| VNM156 | 20140417 | 236(2013)a | | 20.34545° | 105.60288° | Vietnam, Ninh Binh, Cuc Phuong National Park | 11.09.2013 | Jonathan Ablett, Hao Van Luong, Fred Naggs & Sang Van Pham | *Cyclophorus cucphuongensis* Oheimb, sp. nov. | | slightly deviating | | | | |
| VNM157 | 20140418 | 237(2013)a | | 20.34545° | 105.60288° | Vietnam, Ninh Binh, Cuc Phuong National Park | 11.09.2013 | Jonathan Ablett, Hao Van Luong, Fred Naggs & Sang Van Pham | *Cyclophorus cucphuongensis* Oheimb, sp. nov. | | widespread morphotype | | | | |
| VNM158 | 20140511/2 | 330(2013)b | | 20.33253° | 105.61333° | Vietnam, Ninh Binh, Cuc Phuong National Park | 08.09.2013 | Jonathan Ablett, Hao Van Luong, Fred Naggs & Sang Van Pham | *Cyclophorus cucphuongensis* Oheimb, sp. nov. | | widespread morphotype | | | | |
| VNM159 | 20140511/3 | 330(2013)c | | 20.33253° | 105.61333° | Vietnam, Ninh Binh, Cuc Phuong National Park | 08.09.2013 | Jonathan Ablett, Hao Van Luong, Fred Naggs & Sang Van Pham | *Cyclophorus cucphuongensis* Oheimb, sp. nov. | | widespread morphotype | | | | |
| VNM160 | 20160707 | V146(2008)b | 10/05 | 20.258974° | 105.706492° | Vietnam, Ninh Binh, Cuc Phuong National Park | 20.05.2008 | Hao Van Luong & Fred Naggs | *Cyclophorus cucphuongensis* Oheimb, sp. nov. | | widespread morphotype | | | | |
| VNM161 | 20140331/5 | 151(2013)e | 10/04 | 20.29562° | 105.66362° | Vietnam, Ninh Binh, Cuc Phuong National Park | 09.09.2013 | Jonathan Ablett, Hao Van Luong, Fred Naggs & Sang Van Pham | *Cyclophorus cucphuongensis* Oheimb, sp. nov. | | widespread morphotype | | | | |
| VNM162 | 20180439 | H38(2009)e | | 20.35055° | 105.59356° | Vietnam, Ninh Binh, Cuc Phuong National Park | 21.04.2009 | Hao Van Luong | *Cyclophorus cucphuongensis* Oheimb, sp. nov. | | slightly deviating | | | | |
| VNM163 | 20140557/1 | 376(2013)a | | 21.66235°–21.65998° | 106.36633°–106.36462° | Vietnam, Lang Son, Huu Lien commune, Huu Lien Nature Reserve | 17.09.2013 | Jonathan Ablett, Hao Van Luong, Fred Naggs & Sang Van Pham | *Cyclophorus subfloridus* Ancey, 1888 (sub-group I) | | widespread morphotype | | | | |
| VNM164 | 20140557/5 | 376(2013)e | | 21.66235°–21.65998° | 106.36633°–106.36462° | Vietnam, Lang Son, Huu Lien commune, Huu Lien Nature Reserve | 17.09.2013 | Jonathan Ablett, Hao Van Luong, Fred Naggs & Sang Van Pham | *Cyclophorus subfloridus* Ancey, 1888 (sub-group I) | | widespread morphotype | | | | |
| VNM165 | 20140557/6 | 376(2013)f | | 21.66235°–21.65998° | 106.36633°–106.36462° | Vietnam, Lang Son, Huu Lien commune, Huu Lien Nature Reserve | 17.09.2013 | Jonathan Ablett, Hao Van Luong, Fred Naggs & Sang Van Pham | *Cyclophorus subfloridus* Ancey, 1888 (sub-group I) | | widespread morphotype | | | | |
| VNM166 | 20140576 | 395(2013)a | | 21.65948° | 106.36535° | Vietnam, Lang Son, Yen Thinh commune | 18.09.2013 | Jonathan Ablett, Hao Van Luong, Fred Naggs & Sang Van Pham | *Cyclophorus subfloridus* Ancey, 1888 (sub-group I) | | slightly deviating | | | | |
| VNM167 | 20110582/17 | EB(2011)q | | 22.13137° | 106.58715° | Vietnam, Lang Son, Trung Quan commune | 23.05.2011 | Hao Van Luong, Fred Naggs & Sang Van Pham | *Cyclophorus subfloridus* Ancey, 1888 (sub-group II) | | slightly deviating | | | | |
| VNM168 | 20110582/18 | EB(2011)r | | 22.13137° | 106.58715° | Vietnam, Lang Son, Trung Quan commune | 23.05.2011 | Hao Van Luong, Fred Naggs & Sang Van Pham | *Cyclophorus subfloridus* Ancey, 1888 (sub-group II) | | slightly deviating | | | | |
| VNM169 | 20110582/19 | EB(2011)s | | 22.13137° | 106.58715° | Vietnam, Lang Son, Trung Quan commune | 23.05.2011 | Hao Van Luong, Fred Naggs & Sang Van Pham | *Cyclophorus subfloridus* Ancey, 1888 (sub-group II) | | slightly deviating | | | | |
| VNM170 | 20110582/20 | EB(2011)t | | 22.13137° | 106.58715° | Vietnam, Lang Son, Trung Quan commune | 23.05.2011 | Hao Van Luong, Fred Naggs & Sang Van Pham | *Cyclophorus subfloridus* Ancey, 1888 (sub-group II) | | deviating | | | | |
| VNM171 | 20110582/22 | EB(2011)v | | 22.13137° | 106.58715° | Vietnam, Lang Son, Trung Quan commune | 23.05.2011 | Hao Van Luong, Fred Naggs & Sang Van Pham | *Cyclophorus subfloridus* Ancey, 1888 (sub-group II) | | widespread morphotype | | | | |
| VNM172 | 20110582/24 | EB(2011)x | | 22.13137° | 106.58715° | Vietnam, Lang Son, Trung Quan commune | 23.05.2011 | Hao Van Luong, Fred Naggs & Sang Van Pham | *Cyclophorus subfloridus* Ancey, 1888 (sub-group II) | | slightly deviating | | | | |
| VNM173 | 20110582/25 | EB(2011)y | | 22.13137° | 106.58715° | Vietnam, Lang Son, Trung Quan commune | 23.05.2011 | Hao Van Luong, Fred Naggs & Sang Van Pham | *Cyclophorus subfloridus* Ancey, 1888 (sub-group II) | | slightly deviating | | | | |
| VNM174 | 20110582/26 | EB(2011)z | | 22.13137° | 106.58715° | Vietnam, Lang Son, Trung Quan commune | 23.05.2011 | Hao Van Luong, Fred Naggs & Sang Van Pham | *Cyclophorus subfloridus* Ancey, 1888 (sub-group II) | | widespread morphotype | | | | |
| VNM175 | 20110582/29 | EB(2011)ac | | 22.13137° | 106.58715° | Vietnam, Lang Son, Trung Quan commune | 23.05.2011 | Hao Van Luong, Fred Naggs & Sang Van Pham | *Cyclophorus subfloridus* Ancey, 1888 (sub-group II) | | deviating | | | | |
| VNM176 | 20180435 | H15(2009)m | | 20.35583° | 105.51088° | Vietnam, Thanh Hoa, Cuc Phuong National Park | 02.05.2009 | Hao Van Luong | *Cyclophorus paracucphuongensis* Oheimb, sp. nov. | | widespread morphotype | | | | |

| | | | | | | | | | | | | | | | |
|---|---|---|---|---|---|---|---|---|---|---|---|---|---|---|---|
| VNM177 | 20180436 | H15(2009)n | | 20.35583° | 105.51088° | Vietnam, Thanh Hoa, Cuc Phuong National Park | 02.05.2009 | Hao Van Luong | *Cyclophorus paracucphuongensis* Oheimb, sp. nov. | | widespread morphotype | | | | |
| VNM178 | 20180437 | H15(2009)o | | 20.35583° | 105.51088° | Vietnam, Thanh Hoa, Cuc Phuong National Park | 02.05.2009 | Hao Van Luong | *Cyclophorus paracucphuongensis* Oheimb, sp. nov. | | widespread morphotype | | | | |
| VNM179 | 20180449 | VN066 | | | | Vietnam, Hoa Binh, Cao Duong commune, bought at market stall (20.72542°, 105.64899°), collected nearby according to the merchant | 26.05.2016 (date of purchase) | Tu Van Do, Takahiro Hirano & Takumi Saito (purchasers) | *Cyclophorus* sp. 1 | | widespread morphotype | | | | |
| VNM180 | 20180450 | VN067 | | | | Vietnam, Hoa Binh, Cao Duong commune, bought at market stall (20.72542°, 105.64899°), collected nearby according to the merchant | 26.05.2016 (date of purchase) | Tu Van Do, Takahiro Hirano & Takumi Saito (purchasers) | *Cyclophorus* sp. 1 | | widespread morphotype | | | | |
| VNM181 | 20180451 | VN068 | | | | Vietnam, Hoa Binh, Cao Duong commune, bought at market stall (20.72542°, 105.64899°), collected nearby according to the merchant | 26.05.2016 (date of purchase) | Tu Van Do, Takahiro Hirano & Takumi Saito (purchasers) | *Cyclophorus* sp. 1 | | widespread morphotype | | | | |
| VNM182 | 20180453 | VN070 | | | | Vietnam, Hoa Binh, Cao Duong commune, bought at market stall (20.72542°, 105.64899°), collected nearby according to the merchant | 26.05.2016 (date of purchase) | Tu Van Do, Takahiro Hirano & Takumi Saito (purchasers) | *Cyclophorus* sp. 1 | | widespread morphotype | | | | |
| VNM183 | 20180458 | VN084 | | 20.38493° | 105.52280° | Vietnam, Hoa Binh, Cuc Phuong National Park | 26.05.2016 | Tu Van Do, Takahiro Hirano & Takumi Saito | *Cyclophorus takumisaitoi* Hirano, sp. nov. | | slightly deviating | | | | |
| VNM184 | 20180454 | VN080 | | 20.38493° | 105.52280° | Vietnam, Hoa Binh, Cuc Phuong National Park | 26.05.2016 | Tu Van Do, Takahiro Hirano & Takumi Saito | *Cyclophorus takumisaitoi* Hirano, sp. nov. | | widespread morphotype | | | | |
