# Supplementary material for: Cryptic diversity of limestone karst inhabiting land snails (Cyclophorus spp.) in northern Vietnam, their evolutionary history and the description of four new species
Source: PLoS One. 2019 Oct 23;14(10):e0222163. doi: 10.1371/journal.pone.0222163 (PMC6808330; doi:10.1371/journal.pone.0222163)
Supplement: S2 Table — Including specimen code, taxon and GenBank accession numbers. (PDF) [file pone.0222163.s002.pdf]

S2 Table. List of additional samples taken from the literature. Including specimen code, taxon and GenBank accession numbers.

Specimen code	Taxon	COI GenBank accession no.	16S GenBank accession no.	28S GenBank accession no.	Reference (sequence data)
OUT001	*Leptopoma vitreum*	JX474650	JX474741	KF319214	[47]
OUT002	*Cyclotus* sp.	JX474649	JX474739	KF319213	[47]
OUT003	*Rhiostoma hainesi*	JX474651	JX474740	KF319215	[47]
JPN001	*Cyclophorus herklotsi*	JX474644	JX474734	KF319208	[47]
JPN002	*Cyclophorus turgidus*	JX474643	JX474733	KF319207	[47]
MYS001	*Cyclophorus semisulcatus*	JX474646	JX474736	KF319210	[47]
MYS002	*Cyclophorus perdix tuba*	JX474647	JX474737	KF319211	[47]
THA001	*Cyclophorus pernobilis*	JX474623	JX474722	KF319187	[47]
THA002	*Cyclophorus bensoni*	JX474574	JX474670	KF319138	[47]
THA003	*Cyclophorus malayanus*	JX474571	JX474659	KF319135	[47]
THA004	*Cyclophorus saturnus*	JX474563	JX474677	KF319127	[47]
THA005	*Cyclophorus* cf. *fulguratus* (group 4f *sensu* [47])	JX474577	JX474657	KF319141	[47]
THA006	*Cyclophorus speciosus*	JX474575	JX474655	KF319139	[47]
THA007	*Cyclophorus cryptomphalus*	JX474594	JX474665	KF319158	[47]
THA008	*Cyclophorus abditus*	JX474619	JX474701	KF319183	[47]
THA009	*Cyclophorus consociatus*	JX474621	JX474702	KF319185	[47]
THA010	*Cyclophorus fulguratus*	KJ407262	KJ407184	KJ407223	[55]
THA011	*Cyclophorus* sp. (*Cyclophorus courbeti sensu* [47])	JX474613	JX474695	KF319177	[47]
THA012	*Cyclophorus haughtoni*	JX474616	JX474698	KF319180	[47]
THA013	*Cyclophorus labiosus*	JX474610	JX474692	KF319174	[47]
THA014	*Cyclophorus occultus*	JX474609	JX474691	KF319173	[47]
THA015	*Cyclophorus aurantiacus*	JX474642	JX474723	KF319206	[47]
THA016	*Cyclophorus cantori*	JX474629	JX474718	KF319193	[47]
THA017	*Cyclophorus expansus*	JX474630	JX474719	KF319194	[47]
THA018	*Cyclophorus diplochilus*	JX474624	JX474715	KF319188	[47]
THA019	*Cyclophorus zebrinus*	JX474632	JX474721	KF319196	[47]
THA020	*Cyclophorus rangunensis*	JX474582	JX474708	KF319146	[47]
THA021	*Cyclophorus volvulus*	JX474586	JX474712	KF319150	[47]
THA022	*Cyclophorus affinis*	JX474590	JX474681	KF319154	[47]
THA023	*Cyclophorus pfeifferi*	JX474591	JX474683	KF319155	[47]
THA024	*Cyclophorus amoenus*	JX474595	JX474660	KF319159	[47]
THA025	*Cyclophorus* sp. (*Cyclophorus subfloridus sensu* [47])	JX474600	JX474666	KF319164	[47]
THA026	*Cyclophorus borealis*	JX474602	JX474668	KF319166	[47]
VNM001	*Cyclophorus* sp. (*Cyclophorus songmaensis sensu* [47])	JX474578	JX474658	KF319142	[47]
VNM029	*Cyclophorus* sp. (*Cyclophorus jourdyi sensu* [47])	JX474645	JX474735	KF319209	[47]
