# Supplementary material for: Cryptic diversity of limestone karst inhabiting land snails (Cyclophorus spp.) in northern Vietnam, their evolutionary history and the description of four new species
Source: PLoS One. 2019 Oct 23;14(10):e0222163. doi: 10.1371/journal.pone.0222163 (PMC6808330; doi:10.1371/journal.pone.0222163)
Supplement: S1 Fig — The phylogeny is based on sequence data from the COI and 16S genes and was used for the bPTP analysis. Bayesian posterior probabilities are provided at the respective nodes. The scale bar indicates the number of substitutions per site according to the applied model of sequence evolution. Specimen codes (S1 and S2 Tables) refer to outgroups (“OUT”) and respective Cyclophorus sampling localities (“VNM”: Vietnam, “THA”: Thailand, “MYS”: Malaysia, “JPN”: Japan). The tree was rooted with the outgroup species Leptopoma vitreum (OUT001). Specimen codes of individuals with identical COI, 16S and 28S sequences are given at the same branch tip; specimen codes of individuals with identical COI and 16S sequences that are given at different branch tips are labelled with the same superscript number. (PDF) [file pone.0222163.s003.pdf]

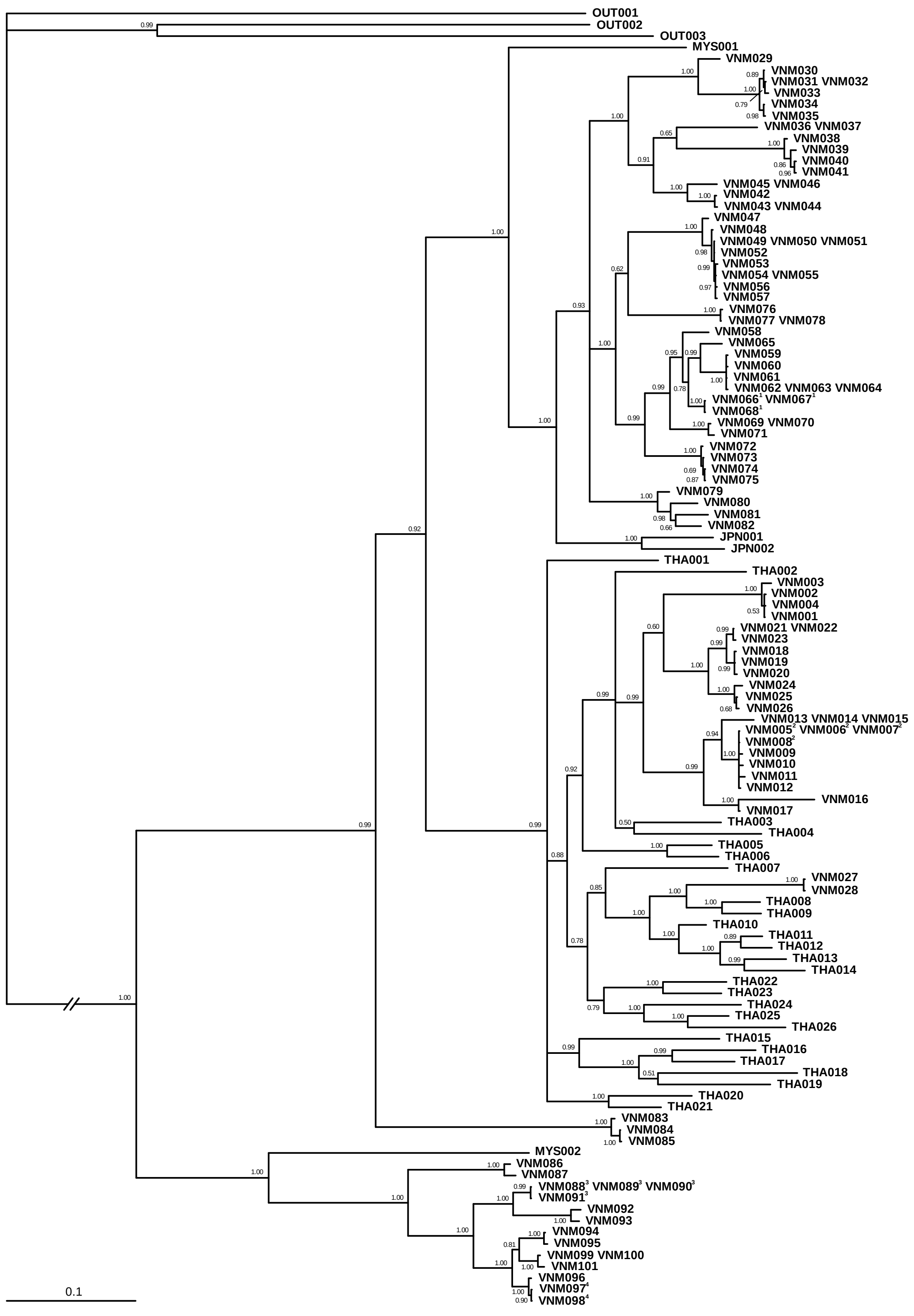

S1 Fig. Additional consensus Bayesian phylogeny of *Cyclophorus* spp. The phylogeny is based on sequence data from the COI and 16S genes and was used for the bPTP analysis. Bayesian posterior probabilities are provided at the respective nodes. The scale bar indicates the number of substitutions per site according to the applied model of sequence evolution. Specimen codes (S1 and S2 Tables) refer to outgroups (“OUT”) and respective *Cyclophorus* sampling localities (“VNM”: Vietnam, “THA”: Thailand, “MYS”: Malaysia, “JPN”: Japan). The tree was rooted with the outgroup species *Leptopoma vitreum* (OUT001). Specimen codes of individuals with identical COI, 16S and 28S sequences are given at the same branch tip; specimen codes of individuals with identical COI and 16S sequences that are given at different branch tips are labelled with the same superscript number.
